# Pain as a predictor of incident frailty in middle-aged and older adults with metabolic dysfunction-associated steatotic liver disease: a prospective cohort study using machine learning and external validation

**DOI:** 10.3389/fendo.2026.1929755

**Published:** 2026-09-28

**Authors:** Huifen Hu, Ling Zhang, Xianbing Zhang, Fuhua Li, Wen Liu

**Affiliations:** Department of Hepatology, No.2 People’s Hospital of Fuyang City, Fuyang Infectious Disease Clinical College of Anhui Medical University, Fuyang, Anhui, China

**Keywords:** CHARLS, depressive symptoms, ELSA, frailty, machine learning, MASLD, pain

## Abstract

**Background:**

Metabolic dysfunction-associated steatotic liver disease (MASLD), defined here by the lipid accumulation product (LAP), is common in middle-aged and older adults and linked to frailty, but factors driving frailty progression remain unclear. We examined the role of pain and depressive symptoms in incident frailty.

**Methods:**

Data came from two prospective national cohorts: the China Health and Retirement Longitudinal Study (CHARLS, 2011–2018) and the English Longitudinal Study of Ageing (ELSA, 2012–2020). Participants with MASLD and no baseline frailty were followed for 7–8 years. Three feature selection methods (LASSO, Boruta, and recursive feature elimination) identified 14 consensus predictors from 31 candidates. Nine machine learning algorithms were trained in CHARLS (70% training, 30% internal test), with ELSA reserved for external validation. Modified Poisson regression (prespecified four-model framework including baseline frailty index) estimated relative risks (RR); g-computation estimated population attributable fractions (PAF); and mediation by depressive symptoms was assessed. Net reclassification improvement (NRI) and integrated discrimination improvement (IDI) quantified the incremental value of pain.

**Results:**

The analysis included 3,622 CHARLS (median 56 years, 70% female) and 2,059 ELSA participants (median 64 years, 53% female). Frailty incidence was 24.49% (CHARLS) and 21.18% (ELSA). Logistic regression performed best (internal test AUC 0.740, 95% CI 0.706–0.774; external validation AUC 0.753, 0.728–0.779). Pain was the strongest predictor (mean absolute SHAP value 0.184). After full adjustment including baseline frailty index, pain remained associated with incident frailty (CHARLS: RR 1.219, 95% CI 1.085–1.368; ELSA: 1.310, 1.114–1.539). Under stated assumptions, the PAF was 8.01% (3.14%–12.66%) in CHARLS and 11.98% (4.76%–19.27%) in ELSA. Adding pain improved reclassification and discrimination (continuous NRI 0.397 and 0.409; IDI 0.024 and 0.023). Depressive symptoms mediated 74.0% (CHARLS) and 46.1% (ELSA) of the total effect; associations were consistent across most subgroups and strongest at baseline FI <0.10 (CHARLS: RR 1.794, 1.289–2.496; ELSA: 1.583, 1.125–2.227).

**Conclusion:**

Pain is a strong predictor of incident frailty in MASLD, with depressive symptoms as the principal mediator. Whether pain reduction modifies frailty risk requires interventional evidence. Routine pain screening could facilitate frailty risk assessment; an online calculator is provided for individualized risk prediction (https://baozhadekele.shinyapps.io/MASLD_Frailty/).

## Graphical Abstract

**Figure:**
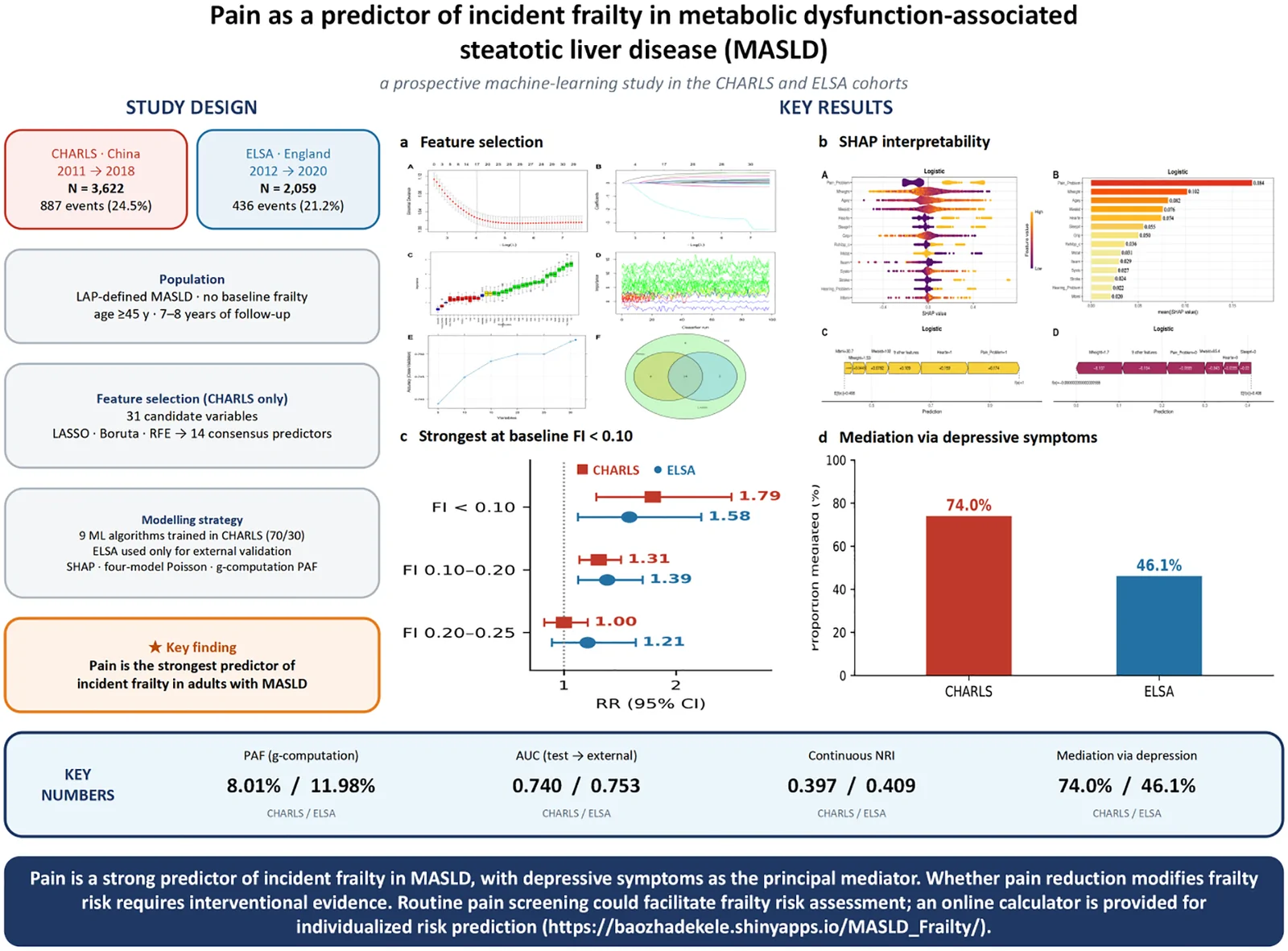

## Introduction

Metabolic dysfunction-associated steatotic liver disease (MASLD), defined here by the lipid accumulation product (LAP), is now recognized as the most prevalent chronic liver condition worldwide, affecting approximately one-third of the global adult population (1, 2). Its rising incidence mirrors the epidemics of obesity and type 2 diabetes, and MASLD is strongly associated with liver-related morbidity and mortality and with cardiometabolic disease (3, 4). In middle-aged and older adults, its clinical impact extends beyond hepatic outcomes: population-based studies have linked MASLD to decreased physical activity, poorer motor function, difficulties in activities of daily living, and greater multimorbidity (1, 2), suggesting that MASLD may also be related to geriatric syndromes such as frailty.

Frailty is a state of heightened vulnerability to stressors resulting from the age-related decline in physiological reserve (5, 6). It can be defined by the frailty phenotype (weight loss, exhaustion, low activity, slowness, and weakness) or by the cumulative-deficit frailty index, which aggregates symptoms, signs, functional limitations, and comorbidities (7, 8). Frailty strongly predicts falls, hospitalization, institutionalization, and mortality (5, 9, 10). In chronic liver disease, frailty is common; among transplant candidates, its prevalence exceeds 30% in alcohol-associated and non-alcoholic fatty liver disease, and worsening frailty predicts waitlist mortality independently of liver disease severity (10, 11). However, the mechanisms driving frailty in MASLD remain unclear.

Pain and depressive symptoms are highly prevalent in middle-aged and older adults and are closely linked to frailty (12–14). Longitudinal data from the Hispanic Established Populations showed that self-reported pain was associated with 71% higher odds of becoming frail over 18 years (13). Similar associations have been reported in Chinese and US community-dwelling populations, where pain, particularly multisite pain, was dose-dependently associated with frailty risk (14, 15). Depressive symptoms independently predict frailty, and synergistic pain–depression interactions may amplify risk beyond their individual effects (8, 14). Mechanistically, chronic pain and depression share pathways involving chronic inflammation, autonomic dysfunction, and hypothalamic–pituitary–adrenal (HPA) axis alterations, all of which can accelerate biological aging (6, 7).

The pain–frailty relationship is particularly relevant in MASLD. Adults with MASLD frequently report abdominal and musculoskeletal pain, and the disease is also associated with depression and reduced quality of life (16). Moreover, hepatic steatosis and insulin resistance promote systemic inflammation, which may lower the threshold for pain sensitization and depressive symptoms and thereby worsen frailty (4). These findings suggest an interconnected pathway, but its directionality and mediation have not been tested in longitudinal, population-based settings.

Current frailty prediction in community-dwelling older adults relies mainly on clinical judgment and deficit counting. More sophisticated data-driven methods, including machine learning, may allow earlier identification and quantification of modifiable risk factors (17). Several studies have shown that machine learning applied to large cohorts can outperform traditional regression in predicting frailty onset and that adding symptom variables such as pain and depression improves discrimination (8, 17). Yet, translation into practice has been limited by a lack of external validation across populations, and few studies have combined LASSO, Boruta, and recursive feature elimination with multicohort external validation.

The present study addressed these gaps. Using data from the China Health and Retirement Longitudinal Study (CHARLS) and the English Longitudinal Study of Ageing (ELSA), we developed and externally validated a parsimonious machine learning model for incident frailty over 7–8 years. Baseline MASLD was identified by lipid accumulation product (LAP) thresholds, and frailty was defined as a cumulative-deficit index ≥0.25. After excluding participants who were frail at baseline, we applied LASSO, Boruta, and recursive feature elimination to 31 candidate variables in the CHARLS training set and retained the 14 consensus features. Nine machine learning models were fitted, and logistic regression performed best in both internal and external validation. In this model, pain was the strongest predictor of new-onset frailty, followed by height, age, and waist circumference. These findings motivated the subsequent modified Poisson regression, population attributable fraction, and mediation analyses and the construction of a nomogram and an online calculator. Our central hypothesis was that bodily pain is a key predictor of incident frailty in middle-aged and older adults with MASLD and that depressive symptoms mediate this pathway.

## Methods

### Study population and design

This study used data from two large-scale, prospective, nationally representative longitudinal surveys: CHARLS and ELSA. Variable definitions were standardized across cohorts using the harmonized data from the Gateway to Global Aging Data.

CHARLS targets adults aged ≥45 years in China. The 2011 baseline covered 150 county-level units and 450 communities across 28 provinces and enrolled 17,707 participants, who are followed biennially. ELSA targets adults aged ≥50 years in England; it began in 2002–2003 and also follows participants biennially.

For CHARLS, 11,847 participants with complete baseline and blood test data in 2011 were initially included. Exclusions were (1) 2,212 without follow-up (2011–2018); (2) 1,482 missing triglycerides (Tg) or waist circumference; (3) 3,861 not meeting the MASLD criteria; and (4) 670 already frail at baseline. The final sample comprised 3,622 participants.

For ELSA, 8,054 participants with complete baseline and blood test data in 2012 were included. Exclusions were (1) 4,135 without follow-up (2012–2020); (2) 54 missing Tg or waist circumference; (3) 1,546 not meeting the MASLD criteria; and (4) 260 already frail at baseline. The final sample comprised 2,059 participants. The participant flowchart is shown in **Figure 1**.

**Figure 1:**
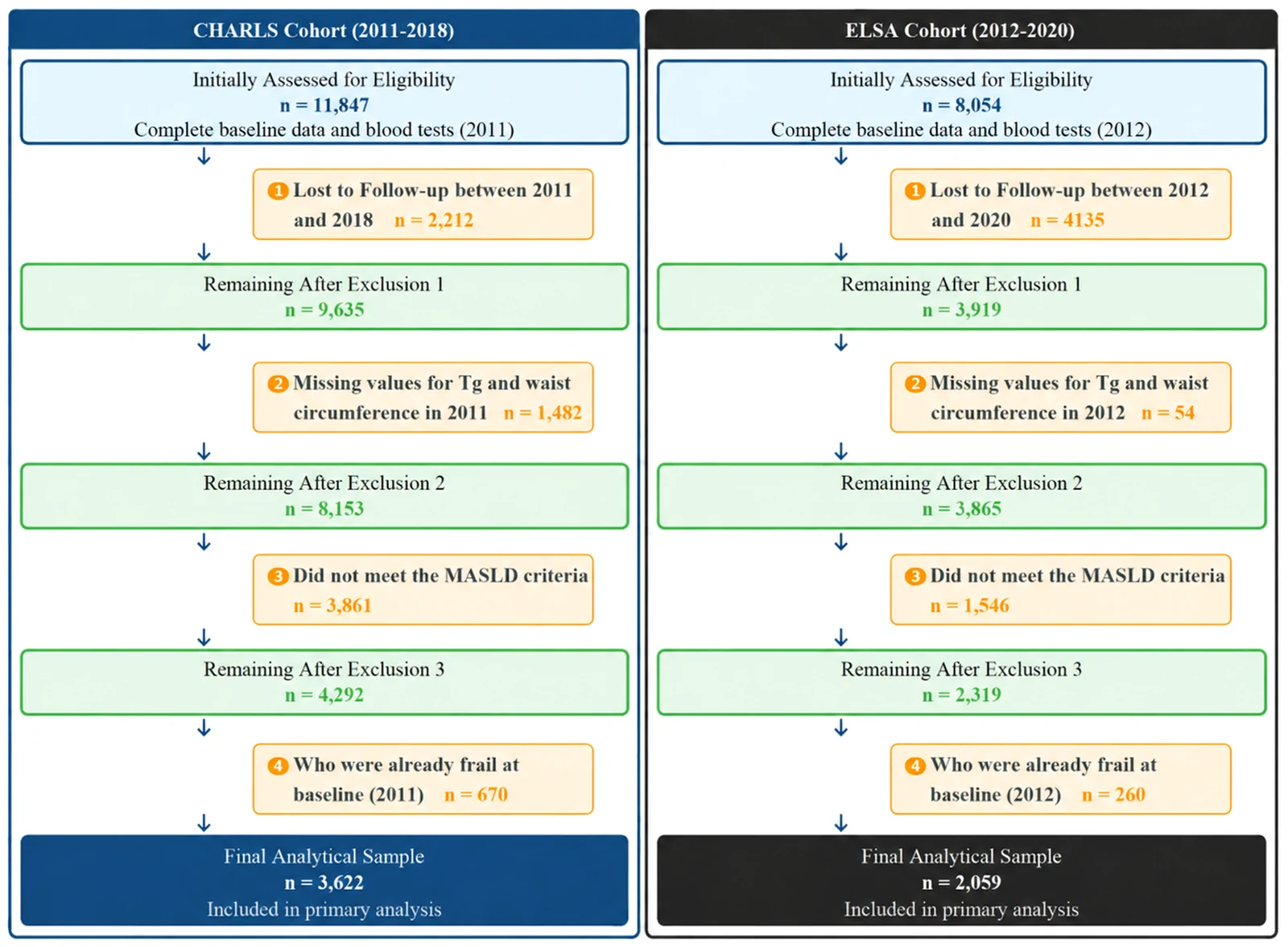
Flowchart of participant enrollment and exclusion for the CHARLS and ELSA cohorts. CHARLS, China health and retirement longitudinal study; ELSA, english longitudinal study of ageing; MASLD, metabolic dysfunction-associated steatotic liver disease; Tg, triglycerides.

Both studies adhered to the principles of the Declaration of Helsinki and received ethical approval from their respective institutional review boards (CHARLS: Institutional Review Board of Peking University, IRB 00001052-11015; ELSA: London Multicentre Research Ethics Committee, MREC/01/2/91). All participants provided written informed consent. As this research is a secondary analysis of anonymized data, no further ethical approval was necessary. This study followed the Strengthening the Reporting of Observational Studies in Epidemiology (STROBE) statement and, for the machine learning analyses, the TRIPOD +AI statement.

### Definition of MASLD

The lipid accumulation product (LAP) was calculated with sex-specific formulas: for men, (waist circumference [cm] − 65) × (Tg [mg/dL]/88.57); for women, (waist circumference [cm] − 58) × (Tg [mg/dL]/88.57). Steatotic liver disease (SLD) was defined as LAP ≥ 30.5 in men or ≥ 23.0 in women, cutoffs validated in Asian populations (18, 19).

MASLD was defined according to the current consensus (20) as the presence of SLD plus at least one cardiometabolic risk factor: body mass index (BMI) ≥25 kg/m², waist circumference ≥94 cm (men) or ≥80 cm (women), fasting glucose (Glu) ≥100 mg/dL, glycated hemoglobin (HbA1c) ≥5.7%, diabetes or use of glucose-lowering drugs, systolic blood pressure ≥130 or diastolic blood pressure ≥85 mmHg, hypertension or use of antihypertensive drugs, Tg ≥150 mg/dL, use of lipid-lowering drugs, or low high-density lipoprotein cholesterol (HDL) (<40 mg/dL in men, <50 mg/dL in women).

### Frailty index and incident frailty

Frailty was assessed with the cumulative-deficit approach (21, 22). In both cohorts, a 33-item frailty index covered chronic diseases, functional limitations (ADL/IADL), self-rated health, and depressive symptoms (CES-D). The CHARLS items are listed in **Supplementary Table S1** and the ELSA items in **Supplementary Table S2**; 29 of the 33 items (≈88%) were identically or closely harmonized. Each deficit was scored from 0 to 1: binary items were scored 0/1; self-rated health (5 levels) was converted to 0, 0.25, 0.5, 0.75, or 1; and CES-D items were recoded as 0, 0.5, or 1 in CHARLS (reverse-coded for “happy”) and as 0/1 in ELSA. The FI was calculated as the sum of the item scores divided by 33. Four items were not harmonized between cohorts (CHARLS-specific: kidney disease, digestive disease, liver disease, and memory problems; ELSA-specific: cataracts, Parkinson’s disease, osteoporosis, and dementia), and the CES-D scoring differed between cohorts; the potential impact of these differences is addressed in the Discussion. For the mediation analyses, a 28-item index excluding the five CES-D items (FI-28) was constructed as the outcome, and a 29-item index restricted to the fully harmonized items (FI-29) was used in the sensitivity analyses.

Frailty was defined as FI ≥0.25 (21–23). Participants who were frail at baseline (CHARLS 2011; ELSA 2012) were excluded. Incident frailty was defined as FI ≥0.25 at follow-up (CHARLS 2018; ELSA 2020). The prediction horizon was therefore approximately 7 years in CHARLS and 8 years in ELSA. Outcomes were adjudicated at the final follow-up wave; intermediate waves were not used, and participants without a final follow-up were excluded (see Discussion).

### Covariates

The following baseline covariates were included for subsequent variable selection: Hearte (self-reported ever had heart problem), Stroke (self-reported ever had stroke), Pain_Problem (harmonized as a binary variable from the CHARLS item “Currently Feel Any Body Pains” and the ELSA item on frequent problems with pain, both yes/no), Physical_Disabilities, Vision_Problem, Hearing_Problem, Agey (age in years), Gender, Mstat (marital status), Smoken (current smoking), Drinkl (drinking in the past year), Rxdiab_c (use of antidiabetic medication), Rxhibp_c (use of antihypertensive medication), Rxdyslip_c (use of lipid-lowering medication), Mheight (measured height, meters), Mweight (measured weight, kilograms), Mwaist (measured waist circumference, centimeters), Mbmi (measured body mass index, kg/m²), Grip (maximum grip strength of left and right hands, kg), Systo (average systolic blood pressure, mmHg), Diasto (average diastolic blood pressure, mmHg), Sleeprl (restless sleep), Hosp1y (hospital stay in the past year), Itearn (post-tax personal annual income), LDL (low-density lipoprotein cholesterol, mg/dL), Hba1c (glycated hemoglobin, %), Wbc (white blood cell count, ×10^9^/L), and Hb (hemoglobin, g/dL).

Based on prior knowledge and literature evidence, the following composite indices associated with adverse outcomes of MASLD were constructed (19, 24, 25): TyG (triglyceride-glucose index, reflecting insulin resistance, calculated as ln[Tg (mg/dL) × Glu (mg/dL)/2]), CHG (cholesterol, high-density lipoprotein, and glucose index, calculated as ln[Total cholesterol (mg/dL) × Glu (mg/dL)/(2 × HDL (mg/dL))]), and CTI (C-reactive protein (CRP)-TyG index, integrating inflammation and insulin resistance, calculated as 0.412 × ln[CRP (mg/L)] + TyG).

After LASSO, Boruta, and RFE, a consensus set of 14 covariates was retained (**Figure 2**): Pain_Problem, Mstat, Rxhibp_c, Sleeprl, Hearte, Stroke, Agey, Mheight, Mwaist, Systo, Grip, Hearing_Problem, Itearn, and Mbmi. The same 14 variables, with identical coding and transformations, were applied in ELSA without any data-dependent modification. The harmonization of the 14 key variables is presented in **Supplementary Table S3**.

**Figure 2:**
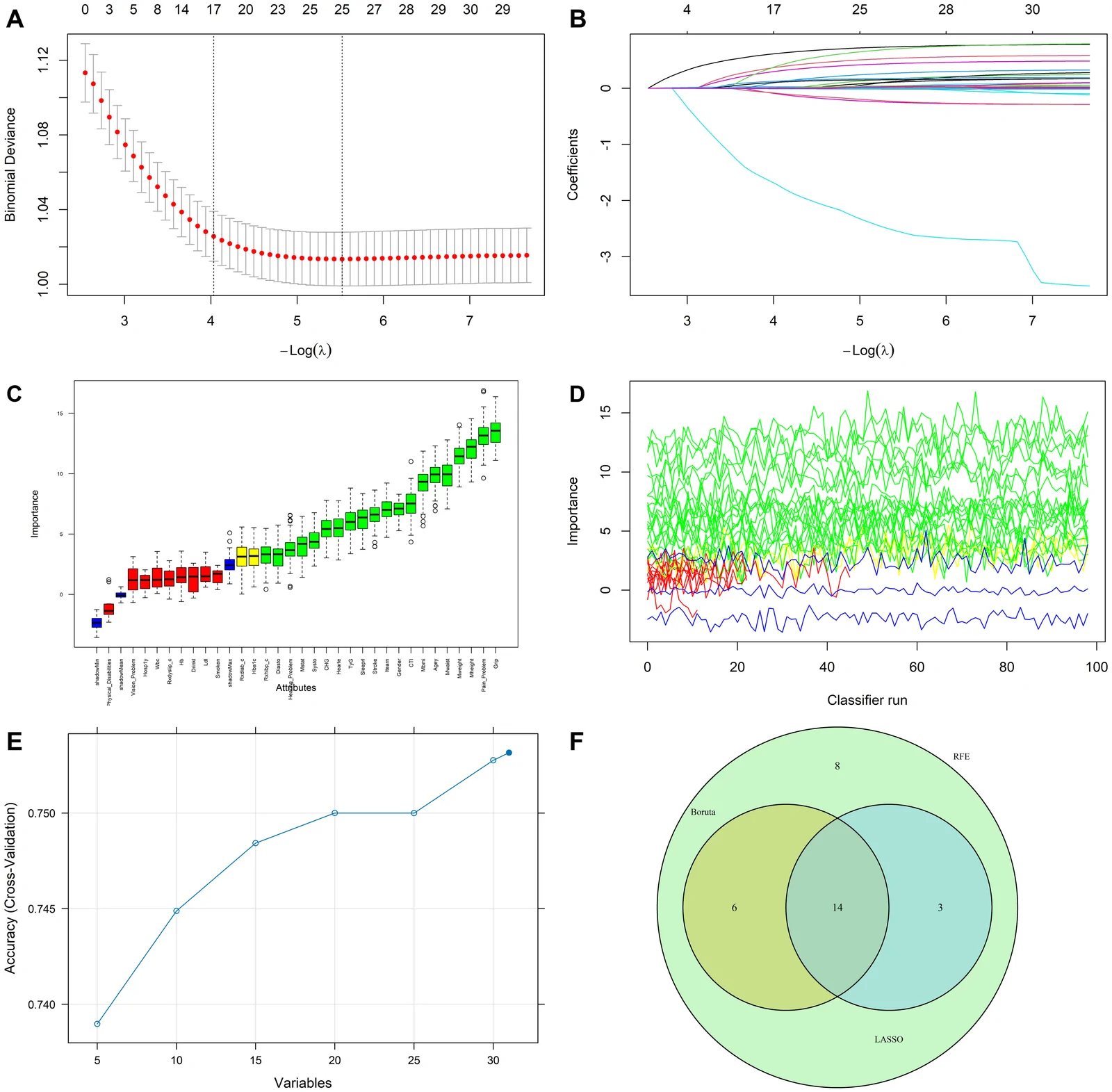
Feature selection workflow and results based on LASSO regression, the Boruta algorithm and RFE. The CHARLS cohort was randomly split (70%/30%), with feature selection performed exclusively within the training set. **(A)** LASSO 10-fold cross-validation curve; λ chosen by the one-standard-error rule. **(B)** LASSO coefficient shrinkage paths; categorical variables were dummy-coded, retaining those with non-zero coefficients. **(C)** Boruta feature importance boxplot (200 iterations) identifying confirmed and rejected variables. **(D)** Convergence trajectories of Boruta importance scores over 200 runs. **(E)** RFE subset selection curve (random forest base learner, 10-fold CV, removing five least important features per step). **(F)** Venn diagram of feature overlap; the intersection of the three methods yielded 14 core predictors for subsequent model development.

### Mediating variables

In CHARLS, seven candidate mediators were measured in 2015 (post-exposure, pre-outcome): depressive symptoms (CES-D-10), grip strength, incident cardiovascular disease and incident heart disease (2013–2015), TyG, CTI, and CHG. In ELSA, depressive symptoms (CES-D-8, 2018) were available for validation of the principal mediator.

### Statistical analysis

Baseline characteristics were compared by frailty status: continuous variables are presented as medians (IQR) and were compared with the Mann–Whitney U test, and categorical variables are presented as n (%) and were compared with the chi-square test. Missing covariates were imputed with the mouse package. For the etiological analyses, five imputed datasets were created and combined with Rubin’s rules; for the machine learning analyses, imputation was performed separately within the training and test sets after the split. Missing-data details are summarized in **Supplementary Table S4** (CHARLS) and **Supplementary Table S5** (ELSA).

CHARLS was split 70%/30% by stratified sampling on frailty status (seed 1234). The complete preprocessing sequence was (1) stratified train/test split; (2) random-forest multiple imputation separately within each set; (3) feature selection within the training set only (LASSO: dummy matrix, 10-fold CV, λ by the 1-SE rule, binomial; Boruta: random forest, 200 iterations; RFE: random forest, 10-fold CV, removing the five least important variables per step, with the subset selected by classification accuracy); (4) class imbalance handled strictly within model development: for the seven algorithms trained in the caret framework, SMOTE (k = 5 nearest neighbors) was applied only inside the training portion of each cross-validation fold, so that validation folds, the held-out test set, and the external cohort were never exposed to synthetic samples; (5) hyperparameter tuning by stratified 10-fold CV using AUC; and (6) model evaluation. After LASSO, Boruta, and RFE, the 14 consensus variables were confirmed (**Figure 2**). The final logistic regression model was refitted on the original, non-synthetic data of the full CHARLS cohort; the nomogram, the online risk calculator, and the full-cohort performance shown in **Figure 3** were derived from this refitted model, so the reported absolute predicted probabilities are not affected by SMOTE.

**Figure 3:**
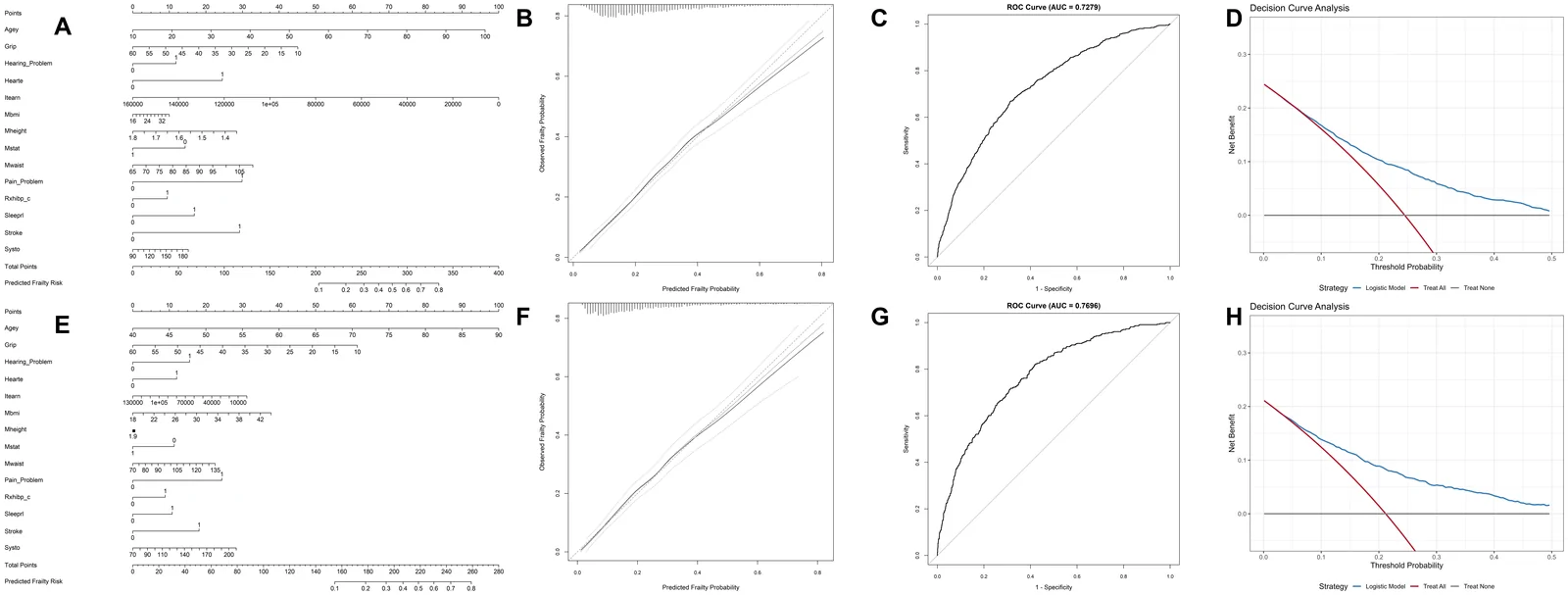
Performance evaluation of the logistic regression model for predicting incident frailty. **(A, E)** Nomograms incorporating the 14 selected predictors. **(B, F)** Calibration curves with 1,000 bootstrap resamples. **(C, G)** Receiver operating characteristic (ROC) curves. **(D, H)** Decision curve analysis (DCA). Panels **(A–D)** display results from the CHARLS cohort, whereas panels E–H display those from the ELSA cohort.

Nine algorithms were trained on the 14 consensus variables: logistic regression, support vector machine (SVM), gradient boosting machine (GBM), neural network (NeuralNetwork), k-nearest neighbors (KNN), XGBoost, AdaBoost, LightGBM, and CatBoost. The final hyperparameters are listed in **Supplementary Table S6**. Feature selection, algorithm selection, hyperparameter tuning, and the probability threshold (the Youden index of the CHARLS training set) were determined exclusively within CHARLS; the final model was frozen before any ELSA application, and ELSA was used only to assess discrimination, calibration, and clinical utility. The final model was selected on the CHARLS internal test set based on a composite of AUC, F1 score, Brier score, calibration, decision-curve net benefit, and the train–test gap. SHAP values (probability scale; training set as background) were used to generate beeswarm, importance bar, and force plots. As a secondary prediction analysis, the final logistic model was refitted with and without the baseline frailty index to quantify the incremental discrimination contributed by the frailty index.

The etiological analysis was specified *a priori* and kept separate from the prediction analysis. Based on the adjustment frameworks of prior pain–frailty cohort studies (13, 26) and excluding frailty-index components, mediating variables, and MASLD-defining variables from the covariate set, four modified Poisson models (log link, HC1 robust standard errors) were fitted: model 1 (crude: pain); model 2 (+ age, gender, marital status, and education); model 3 (+ smoking and drinking); and model 4 (main model: + baseline continuous frailty index). The same model formulas were applied verbatim in both cohorts. Because pain was selected as the exposure on predictive grounds, SHAP importance was interpreted only as the contribution of a variable to the predictions of the fitted model, not as evidence of causal predominance. The adjusted PAF was estimated by g-computation: the predicted frailty risk was averaged under the observed pain distribution and under a counterfactual scenario in which all participants were pain-free, and PAF = (risk observed − risk no pain)/risk observed (27), with 95% CIs from 5,000 bootstrap resamples (1,000 per imputed dataset, pooled across the five imputations).

The incremental value of pain over the baseline model (the other 13 consensus predictors) was quantified with the continuous NRI, the categorical NRI (risk cutoffs 0.2, 0.4, 0.6, and 0.8), and the IDI, all derived from multivariable logistic regression fitted on the complete imputed cohort, with 95% CIs from 1,000 bootstrap resamples; the change in AUC (ΔAUC) with the DeLong test was also reported. The NRI/IDI comparison and the decision-curve threshold ranges are exploratory and should be interpreted together with calibration and clinical judgment.

Subgroup analyses were conducted by sex, age (≥65 vs. <65 years), heart disease, high waist circumference (≥94 cm in men or ≥80 cm in women), BMI (≥28 vs. <28 kg/m²), and baseline frailty index stratum (<0.10, 0.10–0.20, and 0.20–0.25). Within each subgroup, modified Poisson regression with HC1 robust standard errors was adjusted for all model 4 covariates except the subgroup variable. Interactions were tested with product terms (Poisson, log link), and Wald test P values are reported.

The mediation analysis used the FI-28 outcome (the frailty index excluding the five CES-D items) and adjusted for baseline depressive symptoms to address mediator–outcome overlap and preexisting depression. In CHARLS, each candidate mediator was first examined in a single-mediator model; the mediator with the largest indirect effect (depressive symptoms) was then validated in ELSA. Structural equation modeling (lavaan, WLSMV) was fitted in each of the five imputed datasets and pooled with Rubin’s rules; the proportions mediated are reported with their range across imputations. The mediation estimates rely on the usual assumptions of no unmeasured exposure–mediator, mediator–outcome, or exposure–outcome confounding.

A logistic regression nomogram was built from the 14 predictors, converting each to points; total points yield frailty probabilities of 10%–90%. The nomograms and the online calculator were fitted on the full cohort; calibration of the full-cohort model was assessed by bootstrap (1,000 resamples), discrimination by the C-index, and decision curve analysis compared the net benefit against treat-all and treat-none strategies. Held-out test-set and external-validation metrics evaluate the modeling pipeline frozen before any ELSA application. Cohort-specific nomograms were built separately for each cohort for illustration; external validation in ELSA refers to the frozen CHARLS model, and the cohort-specific models are reported separately to distinguish validation from model updating.

As sensitivity analyses, we (1) redefined hepatic steatosis with the triglyceride-glucose index–waist circumference (TyG-WC) product using sex-specific cutoffs (men ≥800, women ≥600; the upper bounds of the sex-specific TyG-WC ranges previously applied in CHARLS (24)), rebuilding the cohort from the full CHARLS pool; (2) excluded participants who reported drinking at baseline; (3) used the FI-29 outcome restricted to the 29 fully harmonized items; and (4) repeated the modified Poisson regression and PAF analyses in complete-case datasets. All model specifications were identical to those of the primary analysis.

## Results

### Baseline characteristics

Of the 3,622 CHARLS participants followed for a median of 7 years, 887 (24.49%) developed incident frailty. Compared with those who remained non-frail (n = 2,735), participants who developed frailty had a higher prevalence of pain (45% vs. 25%), hearing problems (10% vs. 5%), antihypertensive medication use (32% vs. 20%), antidiabetic medication use (7% vs. 3%), and lipid-lowering medication use (8% vs. 5%). They were also older (median 59 vs. 55 years), had a larger waist circumference (91.2 vs. 90.0 cm), and had lower grip strength (28 vs. 32 kg). Laboratory parameters showed modestly higher LDL cholesterol (120.23 vs. 117.53 mg/dL) and white blood cell counts (6.2 vs. 6.1 × 10^9^/L) in the frail group; median HbA1c was 5.2% in both groups, but the distributions differed significantly (P < 0.001). The composite indices TyG (8.94 vs. 8.96, P = 0.91) and CHG (5.47 vs. 5.47, P = 0.061) did not differ significantly between groups, whereas CTI was slightly higher in the frail group (9.10 vs. 9.04, P = 0.006). Baseline characteristics are shown in **Table 1**.

**Table 1:** Baseline characteristics of study participants in the CHARLS cohort.

Variables	Overall	Frailty (2018)		p
	Total (n = 3,622)	No. (n = 2,735)	Yes (n = 887)	
Pain_Problem, n (%)				<0.001
No	2,551 (70)	2,060 (75)	491 (55)	
Yes	1,071 (30)	675 (25)	396 (45)	
Physical_Disabilities, n (%)				0.356
No	3,548 (98)	2,683 (98)	865 (98)	
Yes	74 (2)	52 (2)	22 (2)	
Vision_Problem, n (%)				0.003
No	3,446 (95)	2,619 (96)	827 (93)	
Yes	176 (5)	116 (4)	60 (7)	
Hearing_Problem, n (%)				<0.001
No	3,395 (94)	2,595 (95)	800 (90)	
Yes	227 (6)	140 (5)	87 (10)	
Gender, n (%)				<0.001
Female	2,518 (70)	1,824 (67)	694 (78)	
Male	1,104 (30)	911 (33)	193 (22)	
Mstat, n (%)				<0.001
No	336 (9)	203 (7)	133 (15)	
Yes	3,286 (91)	2,532 (93)	754 (85)	
Smoken, n (%)				0.024
No	2,917 (81)	2,179 (80)	738 (83)	
Yes	705 (19)	556 (20)	149 (17)	
Drinkl, n (%)				<0.001
No	2,682 (74)	1,961 (72)	721 (81)	
Yes	940 (26)	774 (28)	166 (19)	
Rxdiab_c, n (%)				<0.001
No	3,479 (96)	2,650 (97)	829 (93)	
Yes	143 (4)	85 (3)	58 (7)	
Rxhibp_c, n (%)				<0.001
No	2,780 (77)	2,176 (80)	604 (68)	
Yes	842 (23)	559 (20)	283 (32)	
Rxdyslip_c, n (%)				0.001
No	3,421 (94)	2,603 (95)	818 (92)	
Yes	201 (6)	132 (5)	69 (8)	
Sleeprl, n (%)				<0.001
No	3,032 (84)	2,351 (86)	681 (77)	
Yes	590 (16)	384 (14)	206 (23)	
Hosp1y, n (%)				0.004
No	3,346 (92)	2,547 (93)	799 (90)	
Yes	276 (8)	188 (7)	88 (10)	
Hearte, n (%)				<0.001
No	3,231 (89)	2,499 (91)	732 (83)	
Yes	391 (11)	236 (9)	155 (17)	
Stroke, n (%)				<0.001
No	3,569 (99)	2,708 (99)	861 (97)	
Yes	53 (1)	27 (1)	26 (3)	
Agey, median (Q1,Q3)	56 (49, 62)	55 (49, 61)	59 (53.5, 66)	<0.001
Mheight, median (Q1,Q3)	1.57 (1.51, 1.63)	1.57 (1.52, 1.64)	1.54 (1.5, 1.6)	<0.001
Mweight, median (Q1,Q3)	62.2 (55.3, 70.1)	62.6 (55.7, 70.4)	61.1 (54, 69.35)	0.001
Mwaist, median (Q1,Q3)	90.1 (85, 96.6)	90 (85, 96)	91.2 (85.55, 98)	<0.001
Mbmi, median (Q1,Q3)	25.17 (23.05, 27.46)	25.1 (23.05, 27.31)	25.42 (23.06, 28.06)	0.029
Systo, median (Q1,Q3)	128.5 (116.5, 143.5)	127.5 (116, 141.5)	131 (119, 148)	<0.001
Diasto, median (Q1,Q3)	76.5 (69, 85)	76 (69, 84.5)	76.5 (69.5, 85.5)	0.111
Itearn, median (Q1,Q3)	0 (0, 0)	0 (0, 0)	0 (0, 0)	<0.001
Ldl, median (Q1,Q3)	118.69 (95.88, 142.66)	117.53 (95.49, 141.11)	120.23 (98.97, 146.91)	0.005
Hba1c, median (Q1,Q3)	5.2 (4.9, 5.5)	5.2 (4.9, 5.5)	5.2 (4.9, 5.6)	<0.001
Wbc, median (Q1,Q3)	6.1 (5, 7.3)	6.1 (5, 7.3)	6.2 (5.1, 7.6)	0.011
Hb, median (Q1,Q3)	14.2 (13, 15.5)	14.2 (13, 15.5)	14.1 (12.9, 15.4)	0.278
Grip, median (Q1,Q3)	31 (25, 38.8)	32 (26, 40)	28 (23, 35)	<0.001
TyG, median (Q1,Q3)	8.96 (8.62, 9.36)	8.96 (8.62, 9.36)	8.94 (8.61, 9.36)	0.910
CHG, median (Q1,Q3)	5.47 (5.24, 5.73)	5.47 (5.24, 5.71)	5.47 (5.24, 5.78)	0.061
CTI, median (Q1,Q3)	9.05 (8.62, 9.59)	9.04 (8.61, 9.56)	9.1 (8.66, 9.68)	0.006
FI33_base, median (Q1,Q3)	0.11 (0.06, 0.16)	0.09 (0.05, 0.14)	0.16 (0.11, 0.2)	<0.001

Data are presented as medians (interquartile range, IQR) for continuous variables and as frequencies (percentages) for categorical variables. Group comparisons were performed using the Mann-Whitney U test for continuous variables and the Pearson chi-square test for categorical variables.

CHARLS, China Health and Retirement Longitudinal Study; Mstat, marital status; Rxdiab_c, use of antidiabetic medication; Rxhibp_c, use of antihypertensive medication; Rxdyslip_c, use of lipid-lowering medication; Sleeprl, restless sleep; Hosp1y, hospital stay in the past year; Itearn, post-tax personal annual income (in RMB); LDL, low-density lipoprotein cholesterol; Hba1c, glycated hemoglobin; Wbc, white blood cell count; Hb, hemoglobin; Grip, maximum grip strength; Systo, average systolic blood pressure; Diasto, average diastolic blood pressure; Mbmi, body mass index; Mheight, measured height; Mweight, measured weight; Mwaist, measured waist circumference; TyG, triglyceride-glucose index; CHG, cholesterol, high-density lipoprotein, and glucose index; CTI, C-reactive protein-TyG index.

In ELSA (2,059 participants, of whom 436 developed frailty; incidence 21.18% over 8 years), participants were older than in CHARLS (median 64 vs. 56 years), were less often female (53% vs. 70%), and had a higher BMI (28.54 vs. 25.17 kg/m²) and a higher prevalence of pain (35% vs. 30%), restless sleep (29% vs. 16%), antihypertensive use (31% vs. 23%), and unmarried status (22% vs. 9%). Waist circumference was substantially larger in ELSA (98.3 vs. 90.1 cm), whereas systolic blood pressure was comparable (131 vs. 128.5 mmHg) and median grip strength was identical (31 kg). The prevalence of heart disease (11%) and stroke (1%) was similar between cohorts, but HbA1c was higher in ELSA (5.72% vs. 5.2%), as was the white blood cell count (6.23 vs. 6.1 × 10^9^/L). See **Supplementary Table S7**.

### Model performance and interpretability

Nine machine learning models were trained on the 14 consensus variables (training set: 2,536 participants, 621 events; 44 events per predictor). In the CHARLS internal test set (1,086 participants, 266 events), AUCs ranged from 0.576 (KNN) to 0.740 (logistic regression). Logistic regression achieved an AUC of 0.740 (95% CI 0.706–0.774), followed by SVM (0.739, 0.705–0.773), XGBoost (0.735, 0.702–0.768), and CatBoost (0.729, 0.696–0.763). Logistic regression also achieved the highest F1 score (0.531), a Brier score of 0.160, a calibration slope of 1.127, and a train–test AUC gap of 0.020 and was therefore selected as the final model; no ELSA data were used for model selection. Adding the baseline frailty index to the 14 predictors increased the internal test AUC of logistic regression from 0.740 to 0.803 (**Supplementary Table S8**). A TRIPOD +AI summary is provided in **Supplementary Table S9**.

In the ELSA external validation set (n = 2,059; 436 events), logistic regression again achieved the highest AUC (0.753, 95% CI 0.728–0.779), followed by SVM (0.745, 0.719–0.770), XGBoost (0.723, 0.696–0.749), and GBM (0.719, 0.692–0.745). At the frozen CHARLS threshold, logistic regression had a sensitivity of 0.817, a specificity of 0.540, and an F1 score of 0.463. SHAP mean absolute values (probability scale) ranked Pain_Problem highest (0.184), followed by Mheight (0.102), Agey (0.082), Mwaist (0.076), Hearte (0.074), Sleeprl (0.055), Grip (0.050), Rxhibp_c (0.036), Mstat (0.031), Itearn (0.029), Systo (0.027), Stroke (0.024), Hearing_Problem (0.022), and Mbmi (0.020). ROC, calibration, and decision curves are shown in **Figure 4**, SHAP plots in **Figure 5**, and performance metrics in **Table 2**.

**Figure 4:**
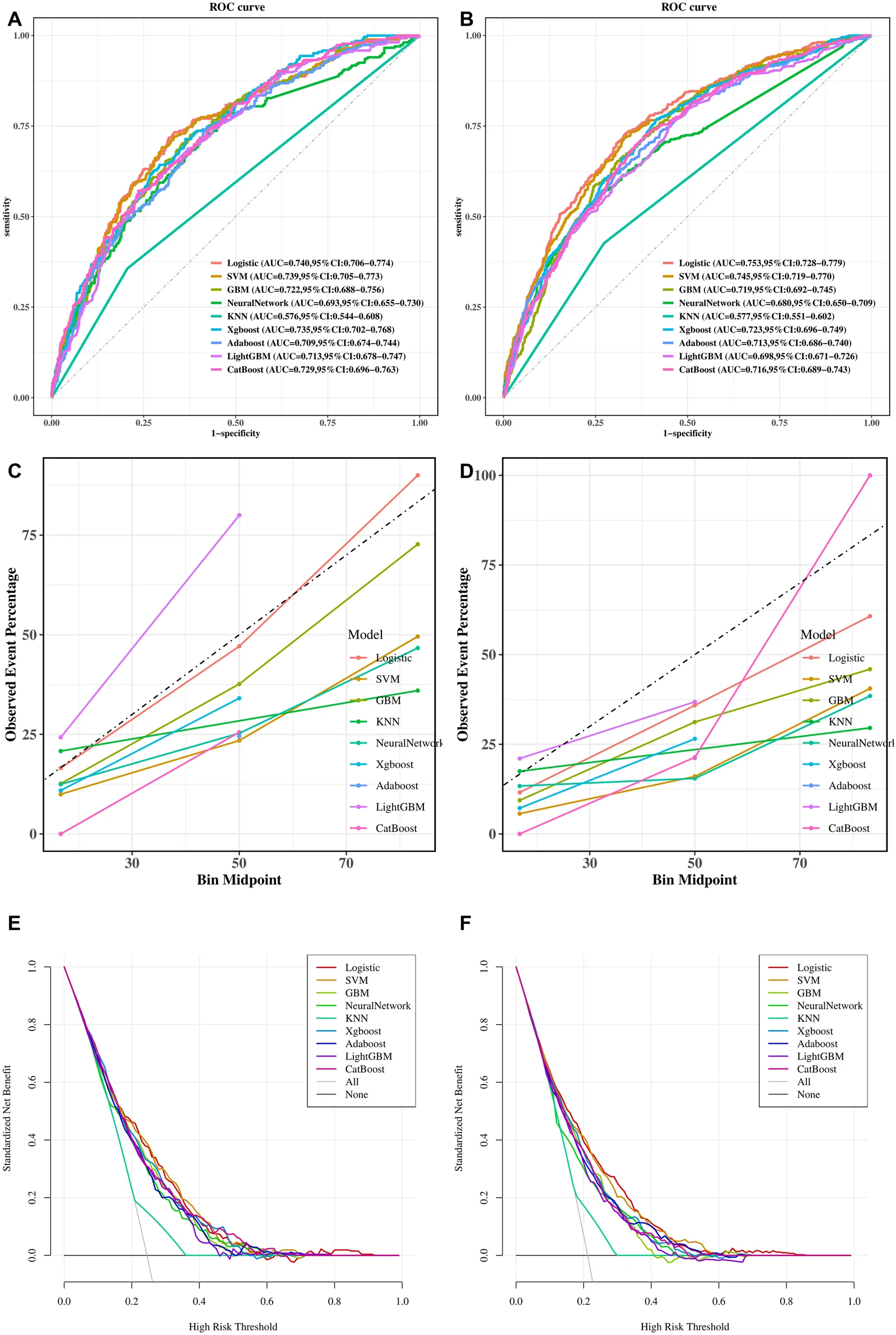
Performance assessment of the nine prediction models. **(A)** ROC curves for the CHARLS internal test set. **(B)** ROC curves for the ELSA external validation set. **(C)** Calibration plots in the CHARLS cohort. **(D)** Calibration plots in the ELSA cohort. **(E)** Decision curve analysis in the CHARLS cohort. **(F)** Decision curve analysis in the ELSA cohort.

**Figure 5:**
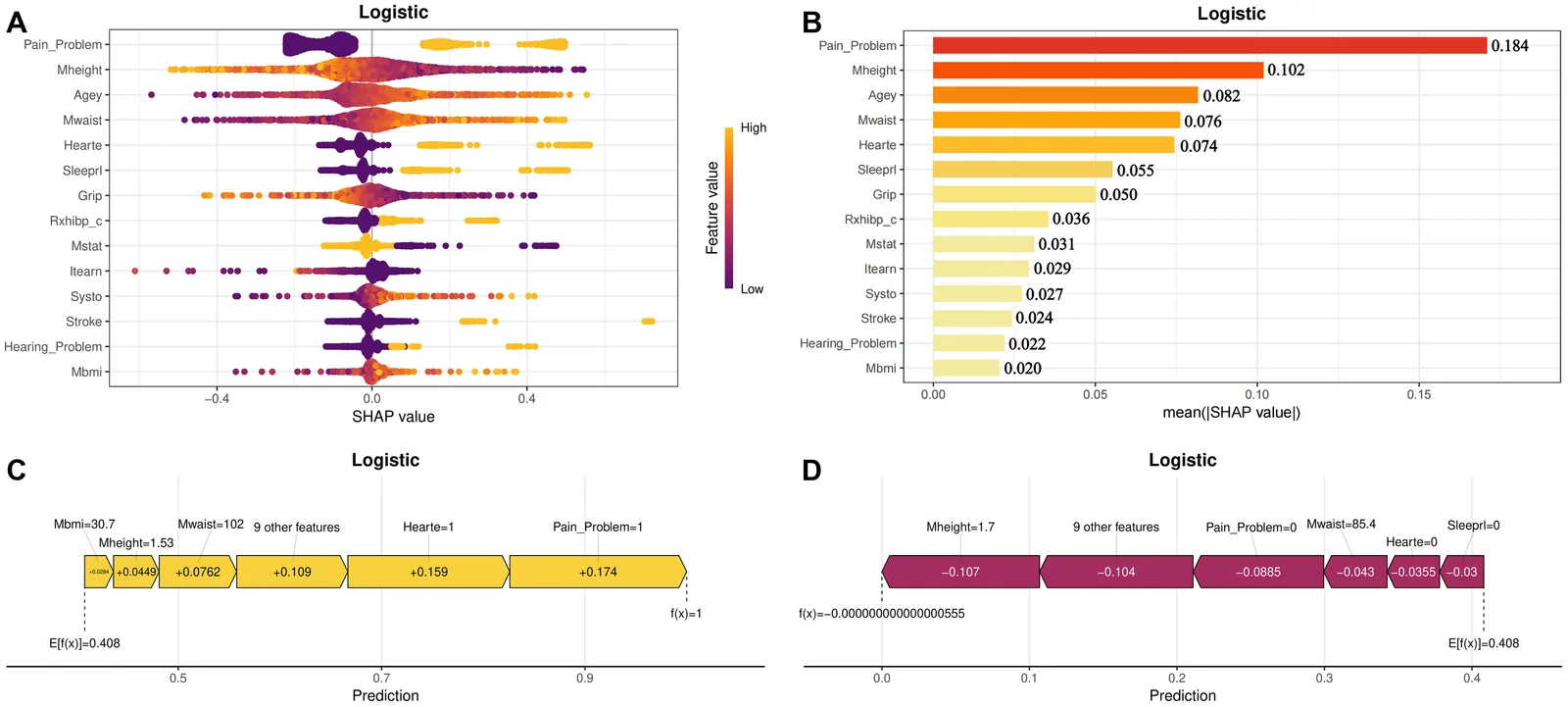
SHAP-based interpretability of the final logistic regression model. **(A)** SHAP beeswarm plot illustrating feature impacts; dots are colored by feature values. **(B)** Global feature importance ranked by the mean absolute SHAP value. **(C, D)** SHAP force plots showing the additive feature contributions to the predicted risk for two representative individuals.

**Table 2:** Performance metrics of each model in the internal test set and the external validation set.

Model	AUC (95% CI)	Threshold	Accuracy	Sensitivity	Specificity	Precision	F1
CHARLS test set							
Logistic	0.740 (0.706-0.774)	0.246	0.690	0.718	0.680	0.422	0.531
SVM	0.739 (0.705-0.773)	0.496	0.680	0.714	0.668	0.411	0.522
GBM	0.722 (0.688-0.756)	0.339	0.653	0.703	0.637	0.386	0.498
NeuralNetwork	0.693 (0.655-0.730)	0.519	0.644	0.650	0.641	0.370	0.472
KNN	0.576 (0.544-0.608)	0.500	0.687	0.357	0.794	0.360	0.358
XGBoost	0.735 (0.702-0.768)	0.374	0.645	0.714	0.622	0.380	0.496
AdaBoost	0.709 (0.674-0.744)	0.480	0.667	0.575	0.696	0.381	0.458
LightGBM	0.713 (0.678-0.747)	0.281	0.634	0.692	0.615	0.368	0.480
CatBoost	0.729 (0.696-0.763)	0.508	0.664	0.643	0.671	0.388	0.484
ELSA external validation set							
Logistic	0.753 (0.728-0.779)	0.246	0.598	0.817	0.540	0.323	0.463
SVM	0.745 (0.719-0.770)	0.496	0.585	0.798	0.527	0.312	0.449
GBM	0.719 (0.692-0.745)	0.339	0.598	0.778	0.550	0.317	0.450
NeuralNetwork	0.680 (0.650-0.709)	0.519	0.623	0.661	0.613	0.314	0.426
KNN	0.577 (0.551-0.602)	0.500	0.663	0.427	0.727	0.296	0.349
XGBoost	0.723 (0.696-0.749)	0.374	0.559	0.819	0.489	0.301	0.440
AdaBoost	0.713 (0.686-0.740)	0.480	0.653	0.656	0.652	0.336	0.444
LightGBM	0.698 (0.671-0.726)	0.281	0.604	0.704	0.577	0.309	0.430
CatBoost	0.716 (0.689-0.743)	0.508	0.614	0.745	0.579	0.322	0.450

AUC, area under the curve; CI, confidence interval; Threshold, probability threshold at which Youden’s index is maximized, frozen in the CHARLS training set and applied identically to the internal test and external validation sets; Accuracy, proportion of correct predictions; Sensitivity, true positive rate (recall); Specificity, true negative rate; Precision, positive predictive value; F1, harmonic mean of precision and sensitivity. Logistic, logistic regression; SVM, support vector machine; GBM, gradient boosting machine; NeuralNetwork, artificial neural network; KNN, k-nearest neighbors; XGBoost, extreme gradient boosting; AdaBoost, adaptive boosting; LightGBM, light gradient boosting machine; CatBoost, categorical boosting.

### Modified poisson regression, PAF, NRI, and IDI

Pain was the exposure in the prespecified four-model framework. In CHARLS, the crude RR was 1.921 (95% CI 1.718–2.148); model 2 RR was 1.882 (1.684–2.102); model 3 RR was 1.871 (1.674–2.090); and the fully adjusted model 4 RR was 1.219 (1.085–1.368, P < 0.001). In ELSA, the corresponding RRs were 1.945 (1.650–2.292), 2.021 (1.726–2.366), 2.014 (1.723–2.355), and 1.310 (1.114–1.539, P = 0.001). By g-computation under model 4, the PAF of pain was 8.01% (95% CI 3.14%–12.66%) in CHARLS and 11.98% (4.76%–19.27%) in ELSA. These fractions are statistically attributable to pain under the stated assumptions and do not imply that treating pain would prevent this proportion of cases. Results are summarized in **Table 3**.

**Table 3:** Modified Poisson regression and population attributable fraction for the association between pain and risk of frailty.

Cohort	Model	RR (95% CI)	P value	PAF (95% CI)
CHARLS	Model 1	1.921 (1.718-2.148)	< 0.001	—
	Model 2	1.882 (1.684-2.102)	< 0.001	—
	Model 3	1.871 (1.674-2.090)	< 0.001	—
	Model 4	1.219 (1.085-1.368)	< 0.001	8.01% (3.14%-12.66%)
ELSA	Model 1	1.945 (1.650-2.292)	< 0.001	—
	Model 2	2.021 (1.726-2.366)	< 0.001	—
	Model 3	2.014 (1.723-2.355)	< 0.001	—
	Model 4	1.310 (1.114-1.539)	0.001	11.98% (4.76%-19.27%)

Model 1: unadjusted. Model 2: adjusted for age, gender, marital status and education. Model 3: additionally adjusted for smoking and drinking. Model 4 (main model): additionally adjusted for baseline frailty index. PAF was estimated by g-computation under Model 4, assuming no unmeasured confounding; 95% CIs were obtained from 5,000 bootstrap resamples (1,000 per imputed dataset, pooled across the five imputations).

CHARLS, China Health and Retirement Longitudinal Study; CI, confidence interval; ELSA, English Longitudinal Study of Ageing; PAF, population attributable fraction; RR, risk ratio.

Adding pain to the other 13 consensus predictors improved discrimination and reclassification: ΔAUC was 0.033 (95% CI 0.015–0.052, DeLong P < 0.001). In CHARLS, continuous NRI = 0.397 (0.328–0.469, P < 0.001), categorical NRI (cutoffs 0.2/0.4/0.6/0.8) = 0.128 (0.063–0.193, P < 0.001), and IDI = 0.024 (0.015–0.035, P < 0.001); in ELSA, continuous NRI = 0.409 (0.305–0.515, P < 0.001), categorical NRI = 0.082 (0.015–0.192, P = 0.016), and IDI = 0.023 (0.011–0.038, P < 0.001) (**Table 4**).

**Table 4:** NRI/IDI data in CHARLS and ELSA.

Cohort	Index	Estimate (95% CI)	P value
CHARLS	Continuous NRI	0.397 (0.328-0.469)	< 0.001
	Categorical NRI	0.128 (0.063-0.193)	< 0.001
	IDI (continuous)	0.024 (0.015-0.035)	< 0.001
ELSA	Continuous NRI	0.409 (0.305-0.515)	< 0.001
	Categorical NRI	0.082 (0.015-0.192)	0.016
	IDI (continuous)	0.023 (0.011-0.038)	< 0.001

Continuous and categorical NRI (cutoffs 0.2/0.4/0.6/0.8) and IDI were estimated using logistic regression comparing the base model (13 predictors without pain) and the extended model (14 predictors including pain) on the complete imputed cohort; 95% CIs from 1,000 bootstrap resamples. CI, confidence interval; IDI, integrated discrimination improvement; NRI, net reclassification improvement.

### Subgroup analyses

In CHARLS, the association between pain and incident frailty was directionally consistent across most predefined subgroups; however, statistically significant interactions were observed for BMI (P = 0.041) and baseline frailty index (overall P = 0.021). Stratification by baseline frailty index revealed a clear gradient: the association was strongest among participants with the lowest baseline frailty burden (FI <0.10: RR = 1.794, 95% CI 1.289–2.496), intermediate at 0.10–0.20 (RR = 1.311, 1.139–1.510), and essentially null at 0.20–0.25 (RR = 1.001, 0.826–1.213). A forest plot is presented in **Figure 6**; detailed subgroup data are provided in **Supplementary Table S10**, and the baseline FI stratification results are shown in **Supplementary Table S11**.

**Figure 6:**
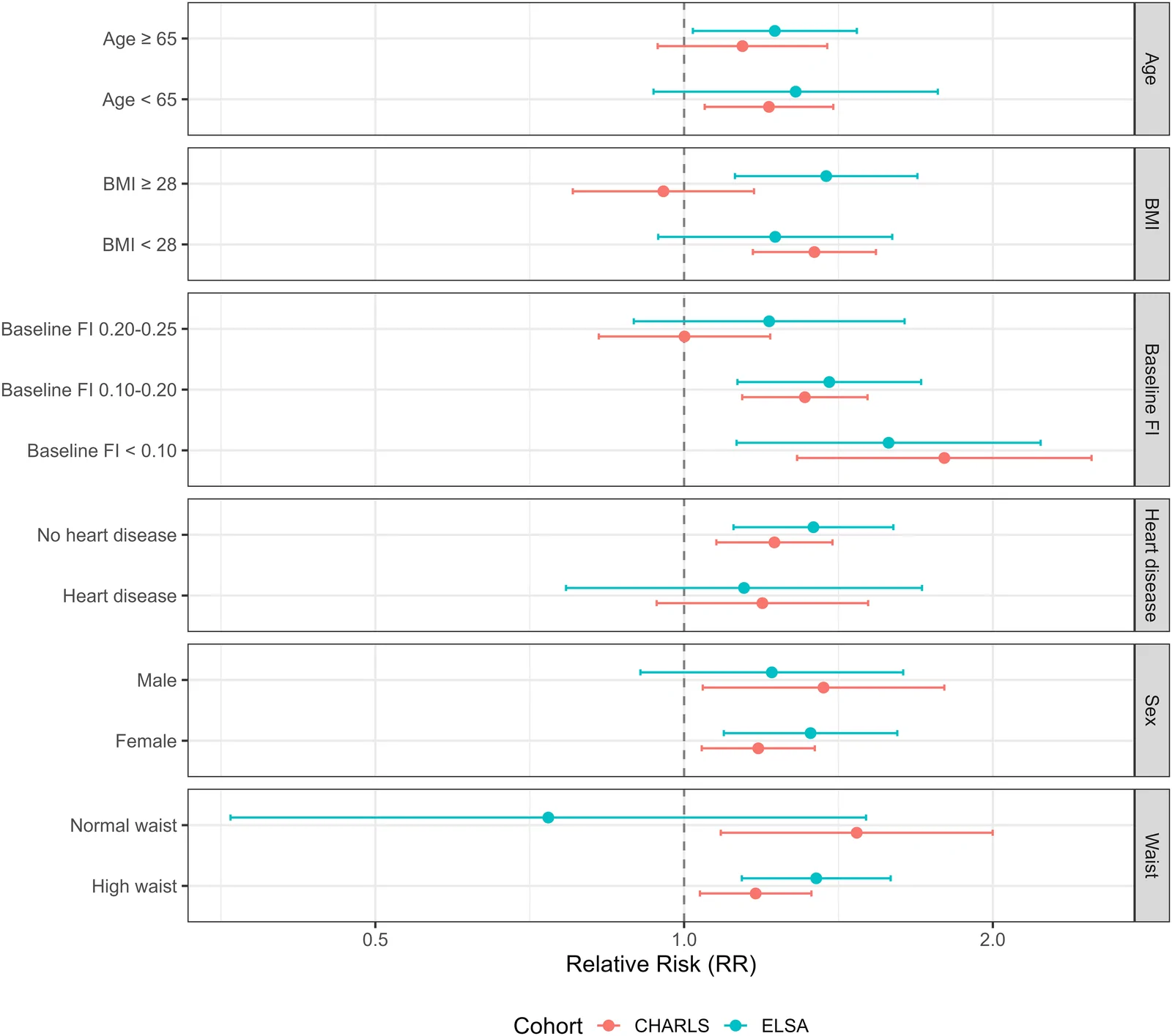
Forest plot of subgroup analysis for pain problem. BMI, body mass index; Interaction P, P-value for interaction; RR, relative risk.

In ELSA, the direction of association was generally consistent across subgroups, with no statistically significant interaction (all P > 0.05). By baseline frailty index, the same gradient was observed, again strongest at FI <0.10: RRs were 1.583 (1.125–2.227) (<0.10), 1.385 (1.127–1.703) (0.10–0.20), and 1.210 (0.893–1.641) (0.20–0.25). In ELSA, neither the overall interaction nor the baseline FI-stratified interaction terms were statistically significant (**Supplementary Tables S11**, **S12**).

### Mediation analysis

Depressive symptoms showed the largest indirect effect (0.237, 95% CI 0.159–0.315, P < 0.001); grip strength showed a smaller but statistically significant indirect effect (0.093, 0.016–0.171, P = 0.019); the remaining five candidates were not statistically significant. In the depression mediation model, the total effect of pain on incident frailty was 0.321 (P < 0.001), the indirect effect via depressive symptoms was 0.237 (P < 0.001) accounting for 74.0% of the total effect (range across imputations 68.8%–80.1%), and the direct effect was 0.084 (P = 0.177). In ELSA (mediator assessed in 2018), the indirect effect via depressive symptoms was 0.246 (P < 0.001), accounting for 46.1% (43.0%–48.1%) of the total effect (0.534, P < 0.001); the direct effect was 0.287 (P < 0.001). Mediation pathway diagrams are shown in **Figure 7** and detailed results in **Supplementary Table S13**.

**Figure 7:**
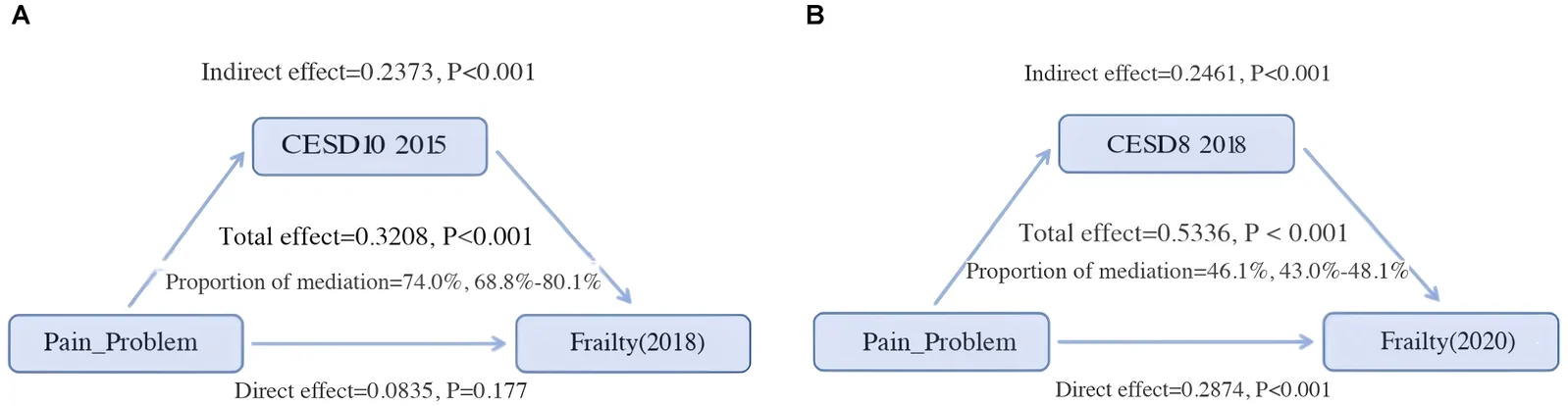
Mediation analysis of the association between pain and incident frailty through depressive symptoms. **(A)** CHARLS cohort **(B)** ELSA cohort.

### Nomogram, calibration, and decision curve analysis

Logistic-based nomograms were constructed from the 14 predictors for both cohorts (coefficients in **Supplementary Tables S14**, **S15**). On the CHARLS cohort, the calibration intercept was 0.095 (95% CI −0.143 to 0.347) and the calibration slope 1.127 (0.937–1.345), with a Brier score of 0.160. DCA showed positive net benefit over “treat-all” and “treat-none” strategies across threshold probabilities up to 0.49 in both cohorts. Within the 0.10–0.30 threshold range (interpreted as exploratory), net benefit ranged from 0.169 to 0.066 in CHARLS and from 0.135 to 0.053 in ELSA (**Supplementary Tables S16**, **S17**). Nomograms, calibration curves, ROC curves, and DCA curves are combined in **Figure 3**.

### Online risk assessment tool

An interactive tool using the final model coefficients was developed and deployed at https://baozhadekele.shinyapps.io/MASLD_Frailty/ for individualized risk prediction. Users can select the target cohort (CHARLS or ELSA) from a drop-down menu and enter the 14 predictors (see **Supplementary Table S3** for variable construction details); the tool applies exactly the coefficients, intercept, variable coding, units, and transformations reported in **Supplementary Tables S14, S15**. For illustrative purposes, the predicted probability is classified into three risk levels (<20%, 20%–40%, ≥40%). These thresholds are research-use examples and have not been clinically validated; the tool is intended for research and health risk communication only and is not a substitute for clinical diagnosis or clinical judgment.

### Sensitivity analysis

Using complete-case datasets with no imputation (participants with all 33 frailty-index items at both waves; CHARLS n = 2,841; ELSA n = 1,579), the fully adjusted RR for pain was 1.190 (95% CI 1.030–1.375, P = 0.018) in CHARLS, with PAF 7.16% (1.20%–13.38%); and in ELSA, RR = 1.377 (1.125–1.686, P = 0.002), with PAF 14.55% (4.75%–24.15%) (**Supplementary Table S18**).

Redefining hepatic steatosis with the TyG-waist circumference product (sex-specific cutoffs, men ≥800, women ≥600; CHARLS full pool, n = 3,829) yielded RR 1.204 (1.078–1.345). Excluding baseline drinkers, the RR was 1.218 (1.074–1.381; n = 2,681) in CHARLS; ELSA was not evaluable for this exclusion because only 149 non-drinkers remained. Using the FI-29 outcome, RRs were 1.224 (1.097–1.366) in CHARLS and 1.236 (1.053–1.451) in ELSA. Detailed results are in **Supplementary Table S19**.

## Discussion

Pain was the strongest predictor of incident frailty in middle-aged and older adults with MASLD in two large prospective cohorts. After adjustment including the baseline frailty index, the relative risk was 1.219 in the China Health and Retirement Longitudinal Study and 1.310 in the English Longitudinal Study of Ageing, and the association remained consistent across most predefined subgroups; it was strongest among participants with the lowest baseline frailty burden (baseline FI <0.10: RR 1.794 in CHARLS and 1.583 in ELSA, both statistically significant) and was attenuated, but not eliminated, after accounting for baseline frailty burden. The addition of pain information significantly improved model discrimination and reclassification beyond the other predictors. Depressive symptoms mediated 74.0% and 46.1% of the total effect in CHARLS and ELSA, respectively. A logistic regression model based on 14 routinely available variables achieved an area under the curve of 0.753 in external validation, with moderate calibration and clinical net benefit. These findings identify pain as a strong predictor of frailty progression in this high-risk metabolic population and point to depressive symptoms as the principal intermediary along this pathway.

Prior investigations have linked pain with frailty in general older populations, with odds ratios exceeding five when pain interferes with daily activities (26). The present study extends this evidence: even after adjustment for demographic characteristics, smoking, drinking, and the baseline frailty index, pain retained a strong independent association with incident frailty. The magnitude of the relative risk (1.219–1.310) aligns with earlier findings linking pain to frailty in other high-risk groups (26, 28). In a systematic review of oral frailty indicators, oral pain was linked only to physical frailty rather than to mortality or disability (29), suggesting that different pain types and severities may affect distinct frailty dimensions. Our work goes further by identifying pain as the strongest predictor among 31 candidate variables in the fitted prediction model, which included age, grip strength, waist circumference, and glycated hemoglobin. This predictive primacy suggests that, in the context of MASLD, the sensory and affective components of pain may accelerate deficit accumulation. However, SHAP importance reflects the contribution of a variable to the predictions of this particular fitted model and depends on variable distributions, correlations, coding, and model specification; it should not be interpreted as evidence that pain is biologically or causally more important than age, grip strength, or other predictors. Adjustment for the baseline frailty index attenuated the association from a crude RR of 1.92 to 1.22, indicating that part of the crude association reflected baseline proximity to the frailty threshold; the residual association supports an independent contribution of pain through mechanisms beyond those captured by standard metabolic risk factors. Possible pathways include pain-induced physical inactivity leading to sarcopenia and obesity, autonomic dysregulation, and increased inflammatory cytokine release, all of which converge on the cumulative-deficit model of frailty. The consistency of the association across both the Chinese and English cohorts and across sex, age, and obesity subgroups strengthens the generalizability of these observations.

Depressive symptoms accounted for a major share (74.0% in CHARLS and 46.1% in ELSA) of the total effect of pain on frailty, a finding consistent with prior evidence on the pain–depression–frailty triad. In the China Health and Retirement Longitudinal Study, depressive symptoms predicted short-term (hazard ratio 2.19) and long-term (1.93) incident frailty, with particular contributions to weakness and exhaustion (30). A recent cross-cohort analysis among older adults showed that depressive symptoms partially mediated the relationship between frailty and cardiovascular disease, with frailty also mediating the effect of depression (31). Beyond cardiovascular outcomes, the joint presence of frailty and depression conferred the highest risk of dementia, and an additive interaction between the two conditions was observed (32). In the digestive disease domain, frailty mediated 30.5% of the effect of depression and depression mediated 45.2% of the effect of frailty, indicating bidirectional mediation (33). Our mediation analysis fits this reciprocal framework: pain may give rise to depressive affect through chronic discomfort, sleep disruption, and activity limitation, which in turn may promote frailty via reduced physical activity, social withdrawal, poor nutrition, and neuroendocrine dysregulation. Depressed individuals also exhibit lower motivation for self-care, higher inflammation, and altered stress hormone profiles, all of which can accelerate deficit accumulation. Screening seven candidate mediators identified depressive symptoms as the predominant pathway (grip strength showed a smaller indirect effect in CHARLS only), suggesting that, in adults with MASLD, the pain–frailty pathway operates primarily through mental health. These estimates were obtained with the FI-28 outcome (the frailty index excluding the CES-D items) and with adjustment for baseline depressive symptoms, so overlap between the mediator and the outcome definition was removed and preexisting depression was accounted for; the mediating pathway was therefore specific to depressive symptoms rather than overt cardiovascular events. This finding has practical implications: screening for depressive symptoms in participants with MASLD who report pain may identify those at the highest frailty risk and provide a target for early psychological or pharmacological intervention.

Integrating pain into a frailty risk prediction model yielded significant improvements in discrimination and net reclassification (continuous NRI 0.397 in CHARLS and 0.409 in ELSA, categorical NRI 0.128 and 0.082, and IDI 0.024 and 0.023), supporting the clinical value of a simple, routinely collected item. The logistic regression model, externally validated in an independent cohort, achieved an AUC of 0.753, outperforming more complex machine learning algorithms including support vector machines, neural networks, and gradient-boosting methods. This performance is comparable with or better than previously published frailty prediction instruments. In a recent systematic review of 22 frailty risk models, AUCs ranged from 0.612 to 0.920, with most studies using logistic regression and common predictors such as age, cognitive function, self-rated health, and functional limitations (34). Another machine learning-based tool derived from the National Health and Nutrition Examination Survey and CHARLS identified eight variables (including age, sex, body mass index, pulse pressure, and two functional items) and achieved an external AUC of 0.850 (35). In Korean community-dwelling older adults, random forest and logistic regression achieved an accuracy of 0.93, but the sample was smaller and the frailty prevalence differed (36). Polygenic risk scores for frailty explained only 1.6–2.1% of the variance (37). For hospitalized patients with decompensated cirrhosis, a random forest model for multidimensional frailty using biomarkers was developed; although it performed well, its applicability to community-dwelling older adults with MASLD remains uncertain (38). Building on these studies, the present work targets the early identification of frailty among community-dwelling older adults with a common chronic liver condition, using pain as a low-cost, readily accessible variable. The SHAP analysis showed that pain made the largest contribution to the model’s predictions of any feature (mean absolute SHAP = 0.184, versus 0.102 for the next-ranked feature).

From a clinical standpoint, these findings suggest that routine pain screening in older adults with MASLD could serve as a simple entry point to frailty risk assessment. Current guidelines for the management of older patients emphasize early frailty evaluation and multimodal care, and our results align with the growing recognition that pain is a geriatric syndrome that warrants systematic assessment. The Pain in Older People with Frailty co-design study concluded that integrating pain management into existing frailty services, rather than creating separate pathways, is the most feasible strategy (39). In the cardiac intensive care unit, the inclusion of frailty (FRAIL scale) improved delirium prediction (40), and preoperative frailty screening has been shown to predict delayed neurocognitive recovery and other poor surgical outcomes (41). These converging lines of evidence point to the value of embedding pain and frailty assessment into routine care for older adults with metabolic liver disease. Under the assumptions of g-computation, an estimated 8.0% (CHARLS) and 12.0% (ELSA) of incident frailty cases were statistically attributable to pain; these estimates describe statistical attribution under the specified assumptions, not the proportion of cases that would be prevented by treating pain. Moreover, the strong mediating role of depressive symptoms implies that combining pain treatment with depression management (e.g., cognitive-behavioral therapy, antidepressants, or exercise) might yield synergistic benefits. Nutritional optimization and physical therapy may also be important, as malnutrition and sarcopenia are highly prevalent in MASLD and interact with both pain and depression (28). The validated logistic regression model, available as an interactive online calculator, can facilitate individualized risk communication and shared decision-making. Within MASLD, several disease-specific mechanisms may further link pain to frailty. Hepatic steatosis and low-grade systemic inflammation may amplify pain sensitization, and visceral pain related to metabolic liver disease may add to musculoskeletal pain. MASLD-associated sarcopenia and reduced physical activity may accelerate deficit accumulation, and the gut–liver–brain axis may connect hepatic inflammation, mood disturbance, and pain perception. These pathways may explain why pain showed strong predictive value in this population beyond the general aging literature.

Several strengths of the present study deserve emphasis. The use of two independent, nationally representative cohorts with harmonized variable definitions and long follow-up (7–8 years) enhances the generalizability and robustness of the findings. The analytical strategy employed multiple variable selection algorithms (LASSO, Boruta, and recursive feature elimination) to identify a parsimonious set of predictors, compared nine machine learning models, and validated the final model externally. Sensitivity analyses on the complete dataset confirmed the stability of the effect estimates, and net reclassification improvement analyses formally quantified the incremental value of adding pain. The mediation analysis with structural equation modeling provided mechanistic insight into the pain–frailty pathway. However, several limitations should be acknowledged. First, frailty was defined using a cumulative-deficit index based on self-reported items; although this is a well-validated approach, it does not capture certain dimensions such as cognitive or social frailty. Second, pain was assessed as a single binary item (presence of any bodily pain), which does not capture intensity, duration, location, or interference. This coarse binary measurement may have attenuated the estimated effect sizes and cannot distinguish musculoskeletal from visceral or neuropathic pain or pain at different sites, so differential associations by pain type remain possible. The pain items also differed between cohorts (current bodily pain in CHARLS versus frequent problems with pain in ELSA), which may contribute to measurement error and cross-cohort heterogeneity. Third, MASLD was diagnosed using the LAP rather than liver biopsy or transient elastography; although LAP is a validated surrogate for hepatic steatosis in large epidemiological studies, misclassification is possible, and the sex-specific LAP thresholds were validated mainly in Asian populations, so their performance in the English population is less certain. The MASLD definition is therefore surrogate-based (LAP-defined), and misclassification cannot be excluded. In addition, four frailty items were not harmonized between cohorts (kidney disease, digestive disease, liver disease, and memory problems in CHARLS; cataracts, Parkinson’s disease, osteoporosis, and dementia in ELSA), and the CES-D items used different scoring (0/0.5/1 versus 0/1), which may affect the cross-cohort comparability of the frailty index; we addressed this with the FI-29 sensitivity analysis restricted to fully harmonized items. Fourth, despite extensive adjustment for confounders, residual confounding, particularly by unmeasured factors such as physical activity level, diet quality, or use of analgesic medications, cannot be excluded. Fifth, although the temporal relationship between pain and frailty was assessed longitudinally, causal inference remains limited; the observed association could be bidirectional, as frailty itself may exacerbate pain perception. Sixth, the mediation analysis relied on the assumption of no unmeasured confounding of the mediator–outcome relationship, which may be violated. Seventh, participants without a final follow-up were excluded and outcomes were assessed only at the final wave; differential attrition and frailty that developed and resolved between waves could not be captured, and we did not apply inverse-probability-of-censoring weighting. Finally, because SHAP importance reflects predictive contribution rather than causal importance, the etiological role of pain rests on the prespecified regression framework rather than on the machine learning ranking.

Future studies should replicate these findings using more granular pain measures (intensity, duration, and location) and validated imaging-based MASLD definitions. Interventional trials evaluating comprehensive pain management and integrated pain–depression approaches are needed. Sex-specific mechanisms, longitudinal biomarker studies (including inflammation, cortisol dysregulation, and gut microbiota), dynamic risk tools that incorporate temporal changes (42), and implementation research embedding pain screening into primary care and hepatology settings are also warranted.

## Conclusions

In two large, independent, prospective cohorts of middle-aged and older adults with MASLD, pain was the strongest predictor of incident frailty. After full adjustment including the baseline frailty index, the relative risks were 1.219 (CHARLS) and 1.310 (ELSA), with g-computation-based PAFs of 8.01% and 11.98% under the specified assumptions. The association remained consistent across most predefined subgroups and was strongest in participants with the lowest baseline frailty burden (baseline FI < 0.10), and adding pain information improved discrimination and reclassification beyond the other predictors. Depressive symptoms mediated 74.0% (CHARLS) and 46.1% (ELSA) of the total effect, supporting the pain–depression–frailty axis as a key pathway in this high-risk population. A logistic regression model based on 14 routinely available variables, frozen in CHARLS and externally validated in ELSA, achieved an AUC of 0.753, moderate calibration, and clinical net benefit.

These findings identify pain as a strong predictor of frailty progression in middle-aged and older adults with MASLD, with an association independent of traditional metabolic and cardiovascular risk factors and of baseline frailty burden, and highlight depressive symptoms as the principal mediator along this pathway. Routine pain screening in this population could serve as a simple gateway to frailty risk assessment. The interactive online risk calculator, constructed from the reported model coefficients, provides a practical, low-cost preliminary screening tool for individualized risk communication and shared decision-making in primary care and hepatology settings; its risk categories are research-use examples and have not been clinically validated. Future interventional trials are needed to test whether comprehensive pain management (pharmacological, psychological, and physical) and integrated pain–depression interventions can reduce frailty incidence in this growing population.

## Data Availability

Publicly available datasets were analyzed in this study. This data can be found here: The data used in this study are publicly available from the China Health and Retirement Longitudinal Study (CHARLS; https://charls.pku.edu.cn/) and the English Longitudinal Study of Ageing (ELSA; https://www.elsa-project.ac.uk/) databases.

## Glossary

AdaBoost: adaptive boosting
CatBoost: categorical boosting
CES-D: Center for Epidemiologic Studies Depression Scale
CHARLS: China Health and Retirement Longitudinal Study
CHG: cholesterol, high-density lipoprotein, and glucose index
CI: confidence interval
CTI: C-reactive protein-TyG index
CVD: cardiovascular disease
DCA: decision curve analysis
ELSA: English Longitudinal Study of Ageing
FI: frailty index
GBM: gradient boosting machine
HbA1c: glycated hemoglobin
HDL: high-density lipoprotein
IADL: instrumental ADL
IDI: integrated discrimination improvement
IQR: interquartile range
KNN: k-nearest neighbors
LAP: lipid accumulation product
LASSO: least absolute shrinkage and selection operator
LDL: low-density lipoprotein
LightGBM: Light Gradient Boosting Machine
MAE: mean absolute error
MASLD: metabolic dysfunction-associated steatotic liver disease
MSE: mean squared error
NeuralNetwork: Neural Network
NRI: net reclassification improvement
PAF: population attributable fraction
RFE: recursive feature elimination
RR: relative risk
SHAP: SHapley Additive exPlanations
SLD: steatotic liver disease
SMOTE: synthetic minority over-sampling technique
SVM: support vector machine
Tg: triglycerides
TyG: triglyceride-glucose index
Wbc: white blood cell
WLSMV: weighted least squares mean and variance adjusted
XGBoost: Extreme Gradient Boosting
