# Supplementary material for: Pain as a predictor of incident frailty in middle-aged and older adults with metabolic dysfunction-associated steatotic liver disease: a prospective cohort study using machine learning and external validation
Source: Front Endocrinol (Lausanne). 2026 Sep 28;17:1929755. doi: 10.3389/fendo.2026.1929755 (PMC13654746; doi:10.3389/fendo.2026.1929755)
Supplement: Supplementary file 1 [file Table1.docx]

| Table S1. Health Variables and Cut-points for the Frailty Index in CHARLS | | |
| --- | --- | --- |
| **No** | **List of 33 Variables included in the frailty index** | **Cut Point** |
| 1 | Some Diff-Bathing, shower | Yes = 1, No = 0 |
| 2 | Some Diff-Dressing | Yes = 1, No = 0 |
| 3 | Some Diff-Eating | Yes = 1, No = 0 |
| 4 | Some Diff-Get in/out bed | Yes = 1, No = 0 |
| 5 | Some Diff-Using the toilet | Yes = 1, No = 0 |
| 6 | Some Diff-controling urination and defecation | Yes = 1, No = 0 |
| 7 | Some Diff-Managing money | Yes = 1, No = 0 |
| 8 | Some Diff-Take medications | Yes = 1, No = 0 |
| 9 | Some Diff-Shop for grocery | Yes = 1, No = 0 |
| 10 | Some Diff-Prepare hot meal | Yes = 1, No = 0 |
| 11 | Some Diff-cleaning house | Yes = 1, No = 0 |
| 12 | Some Diff-Walk 100m | Yes = 1, No = 0 |
| 13 | Some Diff-Get up fr chair | Yes = 1, No = 0 |
| 14 | Some Diff-Lift/carry 10jin | Yes = 1, No = 0 |
| 15 | Some Diff-Clmb sev flt str | Yes = 1, No = 0 |
| 16 | Self-report ever had high blood pressure | Yes = 1, No = 0 |
| 17 | Self-report ever had diabetes | Yes = 1, No = 0 |
| 18 | Self-report ever had cancer | Yes = 1, No = 0 |
| 19 | Self-report ever had lung disease | Yes = 1, No = 0 |
| 20 | Self-report ever had psych problem | Yes = 1, No = 0 |
| 21 | Self-report ever had arthritis | Yes = 1, No = 0 |
| 22 | Self-report ever had dyslipidemia | Yes = 1, No = 0 |
| 23 | Self-report ever had stomach/digestive disease | Yes = 1, No = 0 |
| 24 | Self-report ever had liver disease | Yes = 1, No = 0 |
| 25 | Self-report ever had kidney disease | Yes = 1, No = 0 |
| 26 | Self-report ever had asthma | Yes = 1, No = 0 |
| 27 | Self-report ever had memory problem | Yes = 1, No = 0 |
| 28 | self-report of health | 1.Very good=0 2.Good=0.25 3.Fair=0.5 4.Poor=0.75 5.Very Poor=1 |
| 29 | Everything an effort | 1.Rarely or none of the time < 1 day = 0 2.Some or a little of the time 1-2 days = 0.5 3.Occasionally or a moderate amount of 3 = 0.5 4.Most or all of the time 5-7 days = 1 |
| 30 | Felt depressed | 1.Rarely or none of the time < 1 day = 0 2.Some or a little of the time 1-2 days = 0.5 3.Occasionally or a moderate amount of 3 = 0.5 4.Most or all of the time 5-7 days = 1 |
| 31 | Was happy | 1.Rarely or none of the time < 1 day = 1 2.Some or a little of the time 1-2 days = 0.5 3.Occasionally or a moderate amount of 3 = 0.5 4.Most or all of the time 5-7 days = 0 |
| 32 | Felt lonely | 1.Rarely or none of the time < 1 day = 0 2.Some or a little of the time 1-2 days = 0.5 3.Occasionally or a moderate amount of 3 = 0.5 4.Most or all of the time 5-7 days = 1 |
| 33 | Could not get going | 1.Rarely or none of the time < 1 day = 0 2.Some or a little of the time 1-2 days = 0.5 3.Occasionally or a moderate amount of 3 = 0.5 4.Most or all of the time 5-7 days = 1 |
