# Supplementary material for: Pain as a predictor of incident frailty in middle-aged and older adults with metabolic dysfunction-associated steatotic liver disease: a prospective cohort study using machine learning and external validation
Source: Front Endocrinol (Lausanne). 2026 Sep 28;17:1929755. doi: 10.3389/fendo.2026.1929755 (PMC13654746; doi:10.3389/fendo.2026.1929755)
Supplement: Supplementary file 2 [file Table10.docx]

| **Table S10.** Subgroup analysis for the association between pain and risk of frailty in the CHARLS cohort | | | | | |
| --- | --- | --- | --- | --- | --- |
| **Subgroup** | **N** | **RR** | **CI_lower** | **CI_upper** | **P_interaction** |
| Age < 65 | 2949 | 1.210 | 1.047 | 1.398 | 0.1230 |
| Age >= 65 | 673 | 1.140 | 0.942 | 1.379 | 0.1230 |
| BMI < 28 | 2877 | 1.340 | 1.167 | 1.538 | 0.0410 |
| BMI >= 28 | 745 | 0.955 | 0.779 | 1.170 | 0.0410 |
| No heart disease | 3231 | 1.225 | 1.075 | 1.395 | 0.6830 |
| Heart disease | 391 | 1.192 | 0.940 | 1.512 | 0.6830 |
| Female | 2518 | 1.181 | 1.040 | 1.341 | 0.3580 |
| Male | 1104 | 1.368 | 1.043 | 1.794 | 0.3580 |
| Normal waist | 839 | 1.473 | 1.086 | 1.999 | 0.3360 |
| High waist | 2783 | 1.174 | 1.036 | 1.331 | 0.3360 |
| Baseline FI < 0.10 | 1731 | 1.794 | 1.289 | 2.496 | 0.0210 |
| Baseline FI 0.10-0.20 | 1515 | 1.311 | 1.139 | 1.510 | 0.0210 |
| Baseline FI 0.20-0.25 | 376 | 1.001 | 0.826 | 1.213 | 0.0210 |
| Abbreviations: BMI, body mass index (kg/m²); Baseline FI, baseline frailty index; CI, confidence interval; High waist, waist circumference ≥94 cm in men or ≥80 cm in women; N, number of participants in each subgroup; Normal waist, waist circumference below the sex-specific cut-off; P_interaction, p-value for interaction between the subgroup variable and pain exposure; RR, relative risk estimated by modified Poisson regression with Model 4 adjustment.  Model 4 (main model): adjusted for age, gender, marital status, education, smoking, drinking and baseline frailty index. | | | | | |
