# Supplementary material for: Pain as a predictor of incident frailty in middle-aged and older adults with metabolic dysfunction-associated steatotic liver disease: a prospective cohort study using machine learning and external validation
Source: Front Endocrinol (Lausanne). 2026 Sep 28;17:1929755. doi: 10.3389/fendo.2026.1929755 (PMC13654746; doi:10.3389/fendo.2026.1929755)
Supplement: Supplementary file 3 [file Table11.docx]

| **Table S11.** Association between pain and incident frailty by baseline frailty index stratum. | | | | |
| --- | --- | --- | --- | --- |
| **Cohort** | **Baseline FI stratum** | **N** | **RR (95% CI)** | **P value** |
| CHARLS | <0.10 | 1731 | 1.794 (1.289-2.496) | <0.001 |
|  | 0.10-0.20 | 1515 | 1.311 (1.139-1.510) | <0.001 |
|  | 0.20-0.25 | 376 | 1.001 (0.826-1.213) | 0.993 |
|  | Interaction: Pain × FI (0.10–0.20) |  | 0.737 (0.516-1.052) | 0.093 |
|  | Interaction: Pain × FI (0.20–0.25) |  | 0.594 (0.406-0.869) | 0.007 |
| ELSA | <0.10 | 1307 | 1.583 (1.125-2.227) | 0.008 |
|  | 0.10-0.20 | 639 | 1.385 (1.127-1.703) | 0.002 |
|  | 0.20-0.25 | 113 | 1.210 (0.893-1.641) | 0.218 |
|  | Interaction: Pain × FI (0.10–0.20) |  | 0.943 (0.635-1.399) | 0.771 |
|  | Interaction: Pain × FI (0.20–0.25) |  | 0.966 (0.612-1.526) | 0.882 |
| Stratum-specific relative risks were estimated within each baseline frailty index stratum using Model 3 (age, gender, marital status, education, smoking and drinking; baseline FI omitted within strata); interaction terms were added to Model 3 to test effect modification (reference: FI < 0.10).  CI, confidence interval; FI, frailty index; RR, risk ratio. | | | | |
