# Supplementary material for: Pain as a predictor of incident frailty in middle-aged and older adults with metabolic dysfunction-associated steatotic liver disease: a prospective cohort study using machine learning and external validation
Source: Front Endocrinol (Lausanne). 2026 Sep 28;17:1929755. doi: 10.3389/fendo.2026.1929755 (PMC13654746; doi:10.3389/fendo.2026.1929755)
Supplement: Supplementary file 4 [file Table12.docx]

| **Table S12.** Subgroup analysis for the association between pain and risk of frailty in the ELSA cohort | | | | | |
| --- | --- | --- | --- | --- | --- |
| **Subgroup** | **N** | **RR** | **CI_lower** | **CI_upper** | **P_interaction** |
| Age < 65 | 1098 | 1.285 | 0.934 | 1.768 | 0.1420 |
| Age >= 65 | 961 | 1.226 | 1.020 | 1.474 | 0.1420 |
| BMI < 28 | 932 | 1.227 | 0.944 | 1.596 | 0.8150 |
| BMI >= 28 | 1127 | 1.376 | 1.121 | 1.689 | 0.8150 |
| No heart disease | 1834 | 1.337 | 1.117 | 1.600 | 0.1950 |
| Heart disease | 225 | 1.144 | 0.767 | 1.706 | 0.1950 |
| Female | 1090 | 1.328 | 1.093 | 1.614 | 0.4330 |
| Male | 969 | 1.218 | 0.907 | 1.635 | 0.4330 |
| Normal waist | 198 | 0.737 | 0.361 | 1.505 | 0.1380 |
| High waist | 1861 | 1.345 | 1.138 | 1.590 | 0.1380 |
| Baseline FI < 0.10 | 1307 | 1.583 | 1.125 | 2.227 | 0.9570 |
| Baseline FI 0.10-0.20 | 639 | 1.385 | 1.127 | 1.703 | 0.9570 |
| Baseline FI 0.20-0.25 | 113 | 1.210 | 0.893 | 1.641 | 0.9570 |
| Abbreviations: BMI, body mass index (kg/m²); Baseline FI, baseline frailty index; CI, confidence interval; High waist, waist circumference ≥94 cm in men or ≥80 cm in women; N, number of participants in each subgroup; Normal waist, waist circumference below the sex-specific cut-off; P_interaction, p-value for interaction between the subgroup variable and pain exposure; RR, relative risk estimated by modified Poisson regression with Model 4 adjustment.  Model 4 (main model): adjusted for age, gender, marital status, education, smoking, drinking and baseline frailty index. | | | | | |
