# Supplementary material for: Pain as a predictor of incident frailty in middle-aged and older adults with metabolic dysfunction-associated steatotic liver disease: a prospective cohort study using machine learning and external validation
Source: Front Endocrinol (Lausanne). 2026 Sep 28;17:1929755. doi: 10.3389/fendo.2026.1929755 (PMC13654746; doi:10.3389/fendo.2026.1929755)
Supplement: Supplementary file 5 [file Table13.docx]

| **Table S13.** Mediation analysis of the association between pain and incident frailty. | | | | | | |
| --- | --- | --- | --- | --- | --- | --- |
| **Cohort** | **Analysis** | **Effect** | **Estimate (95% CI)** | **P value** | **Proportion mediated** | **Retained** |
| CHARLS | CHARLS screening (single-mediator) | Indirect via depression (CESD-10, 2015) | 0.2373 (0.1592–0.3154) | <0.001 | 74% (68.8%-80.1%) | Yes |
| CHARLS | CHARLS screening (single-mediator) | Indirect via grip strength (2015) | 0.0933 (0.0155–0.1712) | 0.0188 | 29.1% (26.8%-32%) | Yes |
| CHARLS | CHARLS screening (single-mediator) | Indirect via incident CVD (2013-2015) | 0.0711 (-0.0026–0.1447) | 0.0585 | 22.2% (20.3%-23%) | No |
| CHARLS | CHARLS screening (single-mediator) | Indirect via incident heart disease (2013-2015) | 0.0222 (-0.0516–0.0959) | 0.556 | 6.9% (0.8%-17%) | No |
| CHARLS | CHARLS screening (single-mediator) | Indirect via TyG index (2015) | 0.0426 (-0.0459–0.1311) | 0.345 | 13.3% (1.5%-22.1%) | No |
| CHARLS | CHARLS screening (single-mediator) | Indirect via CTI (2015) | 0.0336 (-0.0482–0.1155) | 0.42 | 10.5% (2.4%-19.6%) | No |
| CHARLS | CHARLS screening (single-mediator) | Indirect via CHG index (2015) | 0.0050 (-0.0541–0.0642) | 0.868 | 1.6% (-0.4%-3.2%) | No |
| ELSA | Final model (depression) | Direct effect (c) | 0.2874 | <0.001 |  |  |
| ELSA | Final model (depression) | Indirect via depression | 0.2461 | <0.001 |  |  |
| ELSA | Final model (depression) | Total effect | 0.5336 | <0.001 |  |  |
| ELSA | Final model (depression) | Proportion mediated by depression | 46.1% (43.0%–48.1%) |  |  |  |
| Candidate mediators were screened in single-mediator models in CHARLS (2015 wave); the mediator with the largest indirect effect (depressive symptoms) was validated in ELSA (2018 wave). Models used the FI-28 outcome (frailty index excluding the five CES-D items) and adjusted for baseline CES-D and the Model 3 covariates (age, gender, marital status, education, smoking, drinking); estimates were pooled across five imputed datasets by Rubin's rules. For screening rows, Estimate is the indirect effect with its 95% CI; for proportion mediated, the range is the minimum–maximum across imputations. CES-D, Center for Epidemiologic Studies Depression scale; CVD, cardiovascular disease; CTI, C-reactive protein–triglyceride–glucose index; CHG, cholesterol, high-density lipoprotein, and glucose index; TyG, triglyceride-glucose index; FI, frailty index. | | | | | | |
