# Supplementary material for: Pain as a predictor of incident frailty in middle-aged and older adults with metabolic dysfunction-associated steatotic liver disease: a prospective cohort study using machine learning and external validation
Source: Front Endocrinol (Lausanne). 2026 Sep 28;17:1929755. doi: 10.3389/fendo.2026.1929755 (PMC13654746; doi:10.3389/fendo.2026.1929755)
Supplement: Supplementary file 6 [file Table14.docx]

| **Table S14.** Logistic regression coefficients for the prediction model in the CHARLS cohort | | | | | |
| --- | --- | --- | --- | --- | --- |
| **Variable** | **Coefficient** | **OR** | **Lower_CI** | **Upper_CI** | **P value** |
| Intercept | -2.2228 | 0.1083 | 0.0122 | 0.9612 | 0.046 |
| Agey | 0.0292 | 1.0296 | 1.0186 | 1.0408 | <0.001 |
| Grip | -0.0246 | 0.9757 | 0.9647 | 0.9868 | <0.001 |
| Hearing_Problem | 0.3232 | 1.3815 | 1.0192 | 1.8726 | 0.0373 |
| Hearte | 0.6683 | 1.9509 | 1.5366 | 2.4769 | <0.001 |
| Itearn | -1.71e-05 | 1.0000 | 1.0000 | 1.0000 | 0.0218 |
| Mbmi | 0.0136 | 1.0137 | 0.9742 | 1.0548 | 0.5021 |
| Mheight | -1.7246 | 0.1783 | 0.0451 | 0.7043 | 0.0139 |
| Mstat | -0.3913 | 0.6762 | 0.5193 | 0.8804 | 0.0037 |
| Mwaist | 0.0199 | 1.0201 | 1.0034 | 1.0371 | 0.0181 |
| Pain_Problem | 0.8157 | 2.2607 | 1.9052 | 2.6824 | <0.001 |
| Rxhibp_c | 0.2578 | 1.2941 | 1.0624 | 1.5763 | 0.0104 |
| Sleeprl | 0.4598 | 1.5838 | 1.2917 | 1.9421 | <0.001 |
| Stroke | 0.7973 | 2.2196 | 1.2374 | 3.9814 | 0.0075 |
| Systo | 0.0041 | 1.0042 | 1.0000 | 1.0083 | 0.0514 |
| Agey, age (years); Grip, grip strength (kg); Hearing_Problem, self-reported hearing problem; Hearte, heart disease; Itearn, individual earnings after tax; Mbmi, body mass index (kg/m²); Mheight, height (metres); Mstat, marital status; Mwaist, waist circumference (cm); Pain_Problem, self-reported bodily pain; Rxhibp_c, antihypertensive medication use; Sleeprl, restless sleep; Stroke, stroke; Systo, systolic blood pressure (mmHg). The final model was refitted on the full CHARLS cohort (n = 3,622); the online calculator and Figure 7 use exactly these coefficients. CI, confidence interval; OR, odds ratio. | | | | | |
