# Supplementary material for: Pain as a predictor of incident frailty in middle-aged and older adults with metabolic dysfunction-associated steatotic liver disease: a prospective cohort study using machine learning and external validation
Source: Front Endocrinol (Lausanne). 2026 Sep 28;17:1929755. doi: 10.3389/fendo.2026.1929755 (PMC13654746; doi:10.3389/fendo.2026.1929755)
Supplement: Supplementary file 7 [file Table2.docx]

| **Table S2.** Health Variables and Cut-points for the Frailty Index in ELSA | | |
| --- | --- | --- |
| **No** | **List of 33 Variables included in the frailty index** | **Cut Point** |
| 1 | Some Diff-Bathing, shower | YES=1 NO=0 |
| 2 | Some Diff-Dressing | YES=1 NO=0 |
| 3 | Some Diff-Eating | YES=1 NO=0 |
| 4 | Some Diff-Get in/out bed | YES=1 NO=0 |
| 5 | Some Diff-Using the toilet | YES=1 NO=0 |
| 6 | Any urinary incontinence | YES=1 NO=0 |
| 7 | Some Diff-Managing money | YES=1 NO=0 |
| 8 | Some Diff-Take medications | YES=1 NO=0 |
| 9 | Some Diff-Shop for grocery | YES=1 NO=0 |
| 10 | Some Diff-Prepare hot meal | YES=1 NO=0 |
| 11 | Some Diff-Doing work around the house or garden | YES=1 NO=0 |
| 12 | Some Diff-Walk 100y | YES=1 NO=0 |
| 13 | Some Diff-Get up fr chair | YES=1 NO=0 |
| 14 | Diff-Lift/carry 10lbs | YES=1 NO=0 |
| 15 | Some Diff-Clmb sev flt str | YES=1 NO=0 |
| 16 | Ever had alzheimer’s/dementia | YES=1 NO=0 |
| 17 | Self-report of health | 1.Excellent=0，2.Very good=0.25，3. good=0.5 4.Fair=0.75 5.Poor=1 |
| 18 | Everything an effort | YES=1 NO=0 |
| 19 | Felt depressed | YES=1 NO=0 |
| 20 | Was happy | YES=0 NO=1 |
| 21 | Felt lonely | YES=1 NO=0 |
| 22 | Could not get going | YES=1 NO=0 |
| 23 | Self-report ever had high blood pressure | YES=1 NO=0 |
| 24 | Self-report ever had diabetes | YES=1 NO=0 |
| 25 | Self-report ever had cancer | YES=1 NO=0 |
| 26 | Self-report ever had lung disease | YES=1 NO=0 |
| 27 | Self-report ever had psych problem | YES=1 NO=0 |
| 28 | Self-report ever had arthritis | YES=1 NO=0 |
| 29 | Self-report ever had asthma | YES=1 NO=0 |
| 30 | Self-report ever had high cholesterol | YES=1 NO=0 |
| 31 | Self-report ever had cataracts | YES=1 NO=0 |
| 32 | Self-report ever had parkinson disease | YES=1 NO=0 |
| 33 | Self-report ever had osteoporosis | YES=1 NO=0 |
