# Supplementary material for: Pain as a predictor of incident frailty in middle-aged and older adults with metabolic dysfunction-associated steatotic liver disease: a prospective cohort study using machine learning and external validation
Source: Front Endocrinol (Lausanne). 2026 Sep 28;17:1929755. doi: 10.3389/fendo.2026.1929755 (PMC13654746; doi:10.3389/fendo.2026.1929755)
Supplement: Supplementary file 8 [file Table3.docx]

| **Table S3.** Harmonization procedure for the 14 consensus predictor variables across CHARLS and ELSA | | | |
| --- | --- | --- | --- |
| **Variables** | **Definition** | **Original coding** | **Harmonized coding** |
| **CHARLS** | | | |
| Pain_Problem | Currently feel any body pains | Yes=1, No=0 | Yes=1, No=0 |
| Mstat | Marital status (married/partnered vs not) | 1 married with spouse present; 3 married but not living with spouse; 4-8 others | Married=1,3 / Unmarried=4,5,7,8 |
| Agey | Age in years | Continuous (years) | Continuous (years) |
| Rxhibp_c | Takes any medication for high blood pressure | Yes=1, No=0 | Yes=1, No=0 |
| Sleeprl | Sleep was restless | 1 rarely; 2 some; 3 often; 4 most of the time | Yes=4 / No=1,2,3 |
| Hearte | Self-report ever had heart disease | Yes=1, No=0 | Yes=1, No=0 |
| Stroke | Self-report ever had stroke | Yes=1, No=0 | Yes=1, No=0 |
| Mheight | Measured height in meters | Continuous (meters) | Continuous (meters) |
| Mwaist | Waist measurement in centimeters | Continuous (centimeters) | Continuous (centimeters) |
| Systo | Average systolic blood pressure (mmHg) | Continuous (mmHg) | Continuous (mmHg) |
| Grip | Maximum grip strength of both hands (kg) | Continuous (kg) | Continuous (kg) |
| Hearing_Problem | Self-reported hearing problem | 1 yes; 2 no | Yes=1, No=0 |
| Itearn | Individual earnings after tax (RMB) | Continuous (RMB) | Continuous (RMB) |
| Mbmi | Measured body mass index (kg/m²) | Continuous (kg/m²) | Continuous (kg/m²) |
| **ELSA** | | | |
| Pain_Problem | Frequent problems with pain | Yes=1, No=0 | Yes=1, No=0 |
| Mstat | Marital status (married/partnered vs not) | 1 married/civil partnership; 3 separated; 4-8 others | Married=1,3 / Unmarried=4,5,7,8 |
| Agey | Age in years | Continuous (years) | Continuous (years) |
| Rxhibp_c | Takes medication for high blood pressure | Yes=1, No=0 | Yes=1, No=0 |
| Sleeprl | Sleep was restless | Yes=1, No=0 | Yes=1, No=0 |
| Hearte | Self-report ever had heart disease | Yes=1, No=0 | Yes=1, No=0 |
| Stroke | Self-report ever had stroke | Yes=1, No=0 | Yes=1, No=0 |
| Mheight | Measured height in meters | Continuous (meters) | Continuous (meters) |
| Mwaist | Waist measurement in centimeters | Continuous (centimeters) | Continuous (centimeters) |
| Systo | Average systolic blood pressure (mmHg) | Continuous (mmHg) | Continuous (mmHg) |
| Grip | Maximum grip strength of both hands (kg) | Continuous (kg) | Continuous (kg) |
| Hearing_Problem | Self-rated hearing (fair/poor vs excellent-very good) | 1 excellent-5 poor | Yes=1 (fair/poor) / No=0 |
| Itearn | Individual employment earnings after tax | Continuous (GBP) | Continuous (GBP) |
| Mbmi | Measured body mass index (kg/m²) | Continuous (kg/m²) | Continuous (kg/m²) |
