# Supplementary material for: Pain as a predictor of incident frailty in middle-aged and older adults with metabolic dysfunction-associated steatotic liver disease: a prospective cohort study using machine learning and external validation
Source: Front Endocrinol (Lausanne). 2026 Sep 28;17:1929755. doi: 10.3389/fendo.2026.1929755 (PMC13654746; doi:10.3389/fendo.2026.1929755)
Supplement: Supplementary file 9 [file Table4.docx]

| **Table S4.** Missing data in CHARLS (N = 3,622). | | |
| --- | --- | --- |
| **Variable** | **Missing_Count** | **Missing_Percent**（%） |
| R4effortl | 323 | 8.9177 |
| R4goingl | 310 | 8.5588 |
| R4depresl | 294 | 8.1171 |
| R4flonel | 280 | 7.7305 |
| R4whappyl | 266 | 7.344 |
| R4shlta | 174 | 4.804 |
| R1walk100a | 121 | 3.3407 |
| R1rxdyslip_c | 88 | 2.4296 |
| R1dyslipe | 84 | 2.3192 |
| R1wbc | 72 | 1.9879 |
| R1hb | 71 | 1.9602 |
| R1depresl | 59 | 1.6289 |
| R1effortl | 54 | 1.4909 |
| R1goingl | 53 | 1.4633 |
| R1flonel | 48 | 1.3252 |
| R1rxdiab_c | 45 | 1.2424 |
| R1diabe | 45 | 1.2424 |
| R1beda | 43 | 1.1872 |
| R1batha | 42 | 1.1596 |
| R1urina | 42 | 1.1596 |
| R1dressa | 42 | 1.1596 |
| R1eata | 42 | 1.1596 |
| R1toilta | 42 | 1.1596 |
| R1whappyl | 41 | 1.132 |
| R1grip | 35 | 0.9663 |
| R1livere | 34 | 0.9387 |
| R1climsa | 34 | 0.9387 |
| R1sleeprl | 32 | 0.8835 |
| R1systo | 31 | 0.8559 |
| R1diasto | 31 | 0.8559 |
| R1hba1c | 26 | 0.7178 |
| R1hibpe | 22 | 0.6074 |
| R1rxhibp_c | 22 | 0.6074 |
| R1mbmi | 21 | 0.5798 |
| R1hearte | 21 | 0.5798 |
| R1kidneye | 21 | 0.5798 |
| R1cancre | 19 | 0.5246 |
| R1ldl | 19 | 0.5246 |
| R1psyche | 18 | 0.497 |
| R1lunge | 17 | 0.4694 |
| R1stroke | 17 | 0.4694 |
| R1asthmae | 17 | 0.4694 |
| R4batha | 15 | 0.4141 |
| R4eata | 15 | 0.4141 |
| R4beda | 15 | 0.4141 |
| R4toilta | 15 | 0.4141 |
| R4urina | 15 | 0.4141 |
| R4dressa | 15 | 0.4141 |
| R4walk100a | 15 | 0.4141 |
| R4chaira | 15 | 0.4141 |
| R4lifta | 15 | 0.4141 |
| R4climsa | 15 | 0.4141 |
| R1digeste | 14 | 0.3865 |
| R1moneya | 13 | 0.3589 |
| R1mheight | 12 | 0.3313 |
| R4moneya | 11 | 0.3037 |
| R4medsa | 11 | 0.3037 |
| R4shopa | 11 | 0.3037 |
| R4mealsa | 11 | 0.3037 |
| R4housewka | 11 | 0.3037 |
| R1memrye | 10 | 0.2761 |
| R1mweight | 9 | 0.2485 |
| Hearing_Problem | 9 | 0.2485 |
| Speech_Impediment | 8 | 0.2209 |
| R1shopa | 7 | 0.1933 |
| R1arthre | 7 | 0.1933 |
| Pain_problem | 7 | 0.1933 |
| R4hearte | 6 | 0.1657 |
| Vision_Problem | 6 | 0.1657 |
| R1glu | 6 | 0.1657 |
| R1smoken | 5 | 0.138 |
| R1itearn | 5 | 0.138 |
| R1medsa | 5 | 0.138 |
| R4dyslipe | 5 | 0.138 |
| Physical_Disabilities | 5 | 0.138 |
| R1hosp1y | 4 | 0.1104 |
| R1lifta | 4 | 0.1104 |
| R4hibpe | 4 | 0.1104 |
| R4arthre | 4 | 0.1104 |
| R4digeste | 4 | 0.1104 |
| R1housewka | 4 | 0.1104 |
| R1hdl | 4 | 0.1104 |
| R1crp | 4 | 0.1104 |
| R1agey | 3 | 0.0828 |
| R4diabe | 3 | 0.0828 |
| R1shlta | 2 | 0.0552 |
| R4lunge | 2 | 0.0552 |
| R4livere | 2 | 0.0552 |
| R1drinkl | 2 | 0.0552 |
| R1mealsa | 2 | 0.0552 |
| R1chaira | 2 | 0.0552 |
| R4stroke | 1 | 0.0276 |
| R4kidneye | 1 | 0.0276 |
