# Supplementary material for: Pain as a predictor of incident frailty in middle-aged and older adults with metabolic dysfunction-associated steatotic liver disease: a prospective cohort study using machine learning and external validation
Source: Front Endocrinol (Lausanne). 2026 Sep 28;17:1929755. doi: 10.3389/fendo.2026.1929755 (PMC13654746; doi:10.3389/fendo.2026.1929755)
Supplement: Supplementary file 10 [file Table5.docx]

| **Table S5.** Missing data in ELSA (N = 2,059). | | |
| --- | --- | --- |
| **Variable** | **Missing_Count** | **Missing_Rate(%)** |
| r10urinai | 228 | 11.0733 |
| r10whappy | 65 | 3.1569 |
| r10going | 61 | 2.9626 |
| r10effort | 60 | 2.914 |
| r10flone | 60 | 2.914 |
| r10depres | 59 | 2.8655 |
| r10shlt | 41 | 1.9913 |
| r6rxhibp | 39 | 1.8941 |
| r6wbc | 34 | 1.6513 |
| r6hba1c | 34 | 1.6513 |
| r6gripsum | 27 | 1.3113 |
| r6mbmi | 21 | 1.0199 |
| r6systo | 15 | 0.7285 |
| r6mheight | 12 | 0.5828 |
| r6whappy | 4 | 0.1943 |
| r6depres | 4 | 0.1943 |
| r6going | 3 | 0.1457 |
| r6sleepr | 2 | 0.0971 |
| r6effort | 2 | 0.0971 |
| r6flone | 2 | 0.0971 |
| r6painfr | 1 | 0.0486 |
| r6mstat | 1 | 0.0486 |
| r6hearte | 0 | 0 |
| r6stroke | 0 | 0 |
| r6agey | 0 | 0 |
| r6mwaist | 0 | 0 |
| r6hibpe | 0 | 0 |
| r6diabe | 0 | 0 |
| r6cancre | 0 | 0 |
| r6lunge | 0 | 0 |
| r6psyche | 0 | 0 |
| r6arthre | 0 | 0 |
| r6asthmae | 0 | 0 |
| r6hchole | 0 | 0 |
| r6catracte | 0 | 0 |
| r6parkine | 0 | 0 |
| r6osteoe | 0 | 0 |
| r6batha | 0 | 0 |
| r6dressa | 0 | 0 |
| r6eata | 0 | 0 |
| r6beda | 0 | 0 |
| r6toilta | 0 | 0 |
| r6urinai | 0 | 0 |
| r6moneya | 0 | 0 |
| r6medsa | 0 | 0 |
| r6shopa | 0 | 0 |
| r6mealsa | 0 | 0 |
| r6housewka | 0 | 0 |
| r6walk100a | 0 | 0 |
| r6chaira | 0 | 0 |
| r6lifta | 0 | 0 |
| r6climsa | 0 | 0 |
| r6alzdeme | 0 | 0 |
| r6shlt | 0 | 0 |
| r10hibpe | 0 | 0 |
| r10diabe | 0 | 0 |
| r10cancre | 0 | 0 |
| r10lunge | 0 | 0 |
| r10psyche | 0 | 0 |
| r10arthre | 0 | 0 |
| r10asthmae | 0 | 0 |
| r10hchole | 0 | 0 |
| r10catracte | 0 | 0 |
| r10parkine | 0 | 0 |
| r10osteoe | 0 | 0 |
| r10batha | 0 | 0 |
| r10dressa | 0 | 0 |
| r10eata | 0 | 0 |
| r10beda | 0 | 0 |
| r10toilta | 0 | 0 |
| r10moneya | 0 | 0 |
| r10medsa | 0 | 0 |
| r10shopa | 0 | 0 |
| r10mealsa | 0 | 0 |
| r10housewka | 0 | 0 |
| r10walk100a | 0 | 0 |
| r10chaira | 0 | 0 |
| r10lifta | 0 | 0 |
| r10climsa | 0 | 0 |
| r10alzdeme | 0 | 0 |
| ragender | 0 | 0 |
