# Supplementary material for: Pain as a predictor of incident frailty in middle-aged and older adults with metabolic dysfunction-associated steatotic liver disease: a prospective cohort study using machine learning and external validation
Source: Front Endocrinol (Lausanne). 2026 Sep 28;17:1929755. doi: 10.3389/fendo.2026.1929755 (PMC13654746; doi:10.3389/fendo.2026.1929755)
Supplement: Supplementary file 11 [file Table6.docx]

| **Table S6.** Final hyperparameters of machine learning models | | |
| --- | --- | --- |
| Model | Hyperparameters | Value |
| Logistic | (none, default) | — |
| SVM (RBF) | sigma | 0.001 |
|  | C | 1 |
| GBM | n.trees | 100 |
|  | interaction.depth | 3 |
|  | shrinkage | 0.1 |
|  | n.minobsinnode | 20 |
| KNN | kmax | 13 |
|  | distance | 1 |
|  | kernel | optimal |
| Neural Network | size | 6 |
|  | decay | 0.6 |
| XGBoost | nrounds | 200 |
|  | max_depth | 3 |
|  | eta | 0.01 |
|  | gamma | 0 |
|  | colsample_bytree | 0.6 |
|  | min_child_weight | 1 |
|  | subsample | 0.6 |
| AdaBoost | mfinal | 200 |
|  | maxdepth | 2 |
|  | coeflearn | Zhu |
| LightGBM | learning_rate | 0.05 |
|  | num_leaves | 31 |
|  | min_data_in_leaf | 20 |
|  | max_depth | 5 |
|  | feature_fraction | 0.8 |
|  | bagging_fraction | 0.8 |
|  | lambda_l1 | 0 |
|  | lambda_l2 | 0 |
|  | nrounds (early stop) | 48 |
| CatBoost | depth | 4 |
|  | learning_rate | 0.03 |
|  | l2_leaf_reg | 3 |
|  | border_count | 64 |
|  | iterations | 50 |
| Logistic, logistic regression; SVM, support vector machine; GBM, gradient boosting machine; NeuralNetwork, artificial neural network; KNN, k-nearest neighbours; Xgboost, extreme gradient boosting; Adaboost, adaptive boosting; LightGBM, light gradient boosting machine; CatBoost, categorical boosting. | | |
