# Supplementary material for: Pain as a predictor of incident frailty in middle-aged and older adults with metabolic dysfunction-associated steatotic liver disease: a prospective cohort study using machine learning and external validation
Source: Front Endocrinol (Lausanne). 2026 Sep 28;17:1929755. doi: 10.3389/fendo.2026.1929755 (PMC13654746; doi:10.3389/fendo.2026.1929755)
Supplement: Supplementary file 12 [file Table7.docx]

| **Table S7.** Baseline characteristics of study participants in the ELSA cohort | | | | |
| --- | --- | --- | --- | --- |
| **Variables** | **Overall** | **Frailty (2020)** | | **p** |
|  | Total (n = 2059) | No (n = 1623) | Yes (n = 436) |  |
| Pain_Problem, n (%) |  |  |  | < 0.001 |
| No | 1347 (65) | 1132 (70) | 215 (49) |  |
| Yes | 712 (35) | 491 (30) | 221 (51) |  |
| Mstat, n (%) |  |  |  | < 0.001 |
| No | 453 (22) | 321 (20) | 132 (30) |  |
| Yes | 1606 (78) | 1302 (80) | 304 (70) |  |
| Rxhibp_c, n (%) |  |  |  | < 0.001 |
| No | 1426 (69) | 1171 (72) | 255 (58) |  |
| Yes | 633 (31) | 452 (28) | 181 (42) |  |
| Sleeprl, n (%) |  |  |  | < 0.001 |
| No | 1455 (71) | 1186 (73) | 269 (62) |  |
| Yes | 604 (29) | 437 (27) | 167 (38) |  |
| Hearte, n (%) |  |  |  | < 0.001 |
| No | 1834 (89) | 1469 (91) | 365 (84) |  |
| Yes | 225 (11) | 154 (9) | 71 (16) |  |
| Stroke, n (%) |  |  |  | < 0.001 |
| No | 2031 (99) | 1609 (99) | 422 (97) |  |
| Yes | 28 (1) | 14 (1) | 14 (3) |  |
| Gender, n (%) |  |  |  | < 0.001 |
| Female | 1090 (53) | 815 (50) | 275 (63) |  |
| Male | 969 (47) | 808 (50) | 161 (37) |  |
| Agey, Median (Q1,Q3) | 64 (59, 69) | 63 (58, 68) | 68 (62, 74) | < 0.001 |
| Mheight, Median (Q1,Q3) | 1.67 (1.6, 1.74) | 1.67 (1.61, 1.75) | 1.64 (1.58, 1.7) | < 0.001 |
| Mwaist, Median (Q1,Q3) | 98.3 (90.8, 105.9) | 98 (90.6, 105.5) | 100.1 (92, 107.55) | 0.004 |
| Systo, Median (Q1,Q3) | 131 (120.5, 142.5) | 130 (120, 142) | 133 (123, 144.5) | 0.001 |
| Grip, Median (Q1,Q3) | 31 (25, 41) | 32 (26, 43) | 28 (22, 34.25) | < 0.001 |
| Mbmi, Median (Q1,Q3) | 28.54 (26.17, 31.36) | 28.28 (26.06, 30.98) | 29.38 (26.82, 33.16) | < 0.001 |
| Wbc, Median (Q1,Q3) | 6.23 (5.21, 7.34) | 6.18 (5.17, 7.27) | 6.4 (5.38, 7.6) | 0.004 |
| Hba1c, Median (Q1,Q3) | 5.72 (5.54, 5.99) | 5.72 (5.54, 5.99) | 5.81 (5.63, 6.18) | < 0.001 |
| FI, Median (Q1,Q3) | 0.08 (0.04, 0.13) | 0.07 (0.04, 0.11) | 0.14 (0.1, 0.19) | < 0.001 |
| Data are presented as medians (interquartile range, IQR) for continuous variables and as frequencies (percentages) for categorical variables. Group comparisons were performed using the Mann‑Whitney U test for continuous variables and the Pearson chi‑square test for categorical variables.  ELSA, The English Longitudinal Study of Ageing; Agey, age in years; Grip, maximum grip strength of left and right hands, kg; Hba1c, glycated hemoglobin, %; Hearte, self‑reported ever had heart problem; Mbmi, body mass index, kg/m²; Mheight, measured height, meters; Mstat, marital status; Mwaist, measured waist circumference, centimeters; Pain_Problem, frequent problems with pain; Rxhibp_c, takes medication for high blood pressure; Sleeprl, restless sleep; Stroke, self‑reported ever had stroke; Systo, average systolic blood pressure, mmHg; Wbc, white blood cell count, ×10⁹/L. | | | | |
