# Supplementary material for: Pain as a predictor of incident frailty in middle-aged and older adults with metabolic dysfunction-associated steatotic liver disease: a prospective cohort study using machine learning and external validation
Source: Front Endocrinol (Lausanne). 2026 Sep 28;17:1929755. doi: 10.3389/fendo.2026.1929755 (PMC13654746; doi:10.3389/fendo.2026.1929755)
Supplement: Supplementary file 13 [file Table8.docx]

| **Table S8.** Discrimination of the logistic regression model with and without baseline frailty index (CHARLS internal test set) | | |
| --- | --- | --- |
| **Model** | **Test AUC** | **Description** |
| Without baseline FI | 0.7403 | consensus 14 predictors |
| With baseline FI | 0.8031 | consensus 14 predictors + baseline FI |
| AUC, area under the receiver operating characteristic curve, estimated in the CHARLS internal test set (n = 1086, 266 events). Without baseline FI: logistic regression with the 14 consensus predictors. With baseline FI: the same model plus the baseline frailty index. | | |
