# Supplementary material for: Pain as a predictor of incident frailty in middle-aged and older adults with metabolic dysfunction-associated steatotic liver disease: a prospective cohort study using machine learning and external validation
Source: Front Endocrinol (Lausanne). 2026 Sep 28;17:1929755. doi: 10.3389/fendo.2026.1929755 (PMC13654746; doi:10.3389/fendo.2026.1929755)
Supplement: Supplementary file 14 [file Table9.docx]

| **Table S9.** TRIPOD summary of prediction model development and internal validation performance (CHARLS) | |
| --- | --- |
| **Metric** | **Value** |
| Events (CHARLS train) | 621 |
| Total (CHARLS train) | 2536 |
| Events (CHARLS test) | 266 |
| Total (CHARLS test) | 1086 |
| Events per variable (EPV, training) | 44.36 |
| predictors | 14 |
| Calibration intercept | 0.0948 |
| Calibration intercept lower | -0.1428 |
| Calibration intercept upper | 0.347 |
| Calibration slope | 1.1267 |
| Calibration slope lower | 0.9368 |
| Calibration slope upper | 1.3453 |
| Brier | 0.1595 |
| AUC | 0.7403 |
| AUC lower | 0.7063 |
| AUC upper | 0.7744 |
| Delta AUC (full-base) | 0.0329 |
| Delta AUC lower | 0.0147 |
| Delta AUC upper | 0.0518 |
| DeLong P | 0.0007 |
| TRIPOD, Transparent Reporting of a multivariable prediction model for Individual Prognosis Or Diagnosis. Metrics were estimated in the CHARLS internal test set (n = 1,086, 266 events) with the logistic regression model using the 14 consensus predictors, fitted on the original (non-synthetic) training set and frozen before evaluation; Events per variable (EPV) were calculated on the training set (621 events / 14 predictors). The comparator model for ΔAUC omitted Pain_Problem. CIs for ΔAUC and calibration intercept/slope were obtained from 1,000 bootstrap resamples; the AUC CI was obtained by the DeLong method. AUC, area under the receiver operating characteristic curve; CI, confidence interval; EPV, events per variable. | |
