# Supplementary material for: Pain as a predictor of incident frailty in middle-aged and older adults with metabolic dysfunction-associated steatotic liver disease: a prospective cohort study using machine learning and external validation
Source: Front Endocrinol (Lausanne). 2026 Sep 28;17:1929755. doi: 10.3389/fendo.2026.1929755 (PMC13654746; doi:10.3389/fendo.2026.1929755)
Supplement: Supplementary file 15 [file DataSheet1.zip › Table S16.docx]

| **Table S16.** Net benefit of the logistic regression model across threshold probabilities in the CHARLS cohort | | | | | | | |
| --- | --- | --- | --- | --- | --- | --- | --- |
| **threshold** | **curve** | **NB** | **NB_lower** | **NB_upper** | **sNB** | **sNB_lower** | **sNB_upper** |
| 0 | model | 0.244935543 | 0.220994475 | 0.270718232 | 1.000000000 | 1.000000000 | 1.000000000 |
| 0 | treat_all | 0.244935543 | 0.220994475 | 0.270718232 | 1.000000000 | 1.000000000 | 1.000000000 |
| 0 | treat_none | 0.000000000 | 0.000000000 | 0.000000000 | 0.000000000 | 0.000000000 | 0.000000000 |
| 0.01 | model | 0.237308630 | 0.213125732 | 0.263351750 | 0.968861548 | 0.964393939 | 0.972789116 |
| 0.01 | treat_all | 0.237308630 | 0.213125732 | 0.263351750 | 0.968861548 | 0.964393939 | 0.972789116 |
| 0.01 | treat_none | 0.000000000 | 0.000000000 | 0.000000000 | 0.000000000 | 0.000000000 | 0.000000000 |
| 0.02 | model | 0.229526065 | 0.205096403 | 0.255834931 | 0.937087617 | 0.928061224 | 0.945022907 |
| 0.02 | treat_all | 0.229526065 | 0.205096403 | 0.255834931 | 0.937087617 | 0.928061224 | 0.945022907 |
| 0.02 | treat_none | 0.000000000 | 0.000000000 | 0.000000000 | 0.000000000 | 0.000000000 | 0.000000000 |
| 0.03 | model | 0.221583034 | 0.196901521 | 0.248163126 | 0.904658554 | 0.890979381 | 0.916684199 |
| 0.03 | treat_all | 0.221583034 | 0.196901521 | 0.248163126 | 0.904658554 | 0.890979381 | 0.916684199 |
| 0.03 | treat_none | 0.000000000 | 0.000000000 | 0.000000000 | 0.000000000 | 0.000000000 | 0.000000000 |
| 0.04 | model | 0.213474524 | 0.188535912 | 0.240331492 | 0.871553885 | 0.853125000 | 0.887755102 |
| 0.04 | treat_all | 0.213474524 | 0.188535912 | 0.240331492 | 0.871553885 | 0.853125000 | 0.887755102 |
| 0.04 | treat_none | 0.000000000 | 0.000000000 | 0.000000000 | 0.000000000 | 0.000000000 | 0.000000000 |
| 0.05 | model | 0.205195309 | 0.180091112 | 0.232334981 | 0.837752275 | 0.814912281 | 0.858216971 |
| 0.05 | treat_all | 0.205195309 | 0.179994184 | 0.232334981 | 0.837752275 | 0.814473684 | 0.858216971 |
| 0.05 | treat_none | 0.000000000 | 0.000000000 | 0.000000000 | 0.000000000 | 0.000000000 | 0.000000000 |
| 0.06 | model | 0.196739940 | 0.172857647 | 0.224168332 | 0.803231483 | 0.777387023 | 0.828050369 |
| 0.06 | treat_all | 0.196739940 | 0.171270718 | 0.224168332 | 0.803231483 | 0.775000000 | 0.828050369 |
| 0.06 | treat_none | 0.000000000 | 0.000000000 | 0.000000000 | 0.000000000 | 0.000000000 | 0.000000000 |
| 0.07 | model | 0.188172043 | 0.164339888 | 0.215826056 | 0.768251273 | 0.738440860 | 0.797235023 |
| 0.07 | treat_all | 0.188102735 | 0.162359651 | 0.215826056 | 0.767968308 | 0.734677419 | 0.797235023 |
| 0.07 | treat_none | 0.000000000 | 0.000000000 | 0.000000000 | 0.000000000 | 0.000000000 | 0.000000000 |
| 0.08 | model | 0.179718152 | 0.156337577 | 0.207302426 | 0.733736515 | 0.700285103 | 0.765749778 |
| 0.08 | treat_all | 0.179277764 | 0.153254864 | 0.207302426 | 0.731938542 | 0.693478261 | 0.765749778 |
| 0.08 | treat_none | 0.000000000 | 0.000000000 | 0.000000000 | 0.000000000 | 0.000000000 | 0.000000000 |
| 0.09 | model | 0.172970676 | 0.149636735 | 0.198682533 | 0.706188548 | 0.666006125 | 0.733908948 |
| 0.09 | treat_all | 0.170258839 | 0.143949973 | 0.198591464 | 0.695116913 | 0.651373626 | 0.733572550 |
| 0.09 | treat_none | 0.000000000 | 0.000000000 | 0.000000000 | 0.000000000 | 0.000000000 | 0.000000000 |
| 0.1 | model | 0.168508287 | 0.140577041 | 0.191630857 | 0.687969925 | 0.633424408 | 0.713196951 |
| 0.1 | treat_all | 0.161039493 | 0.134438306 | 0.189686924 | 0.657477026 | 0.608333333 | 0.700680272 |
| 0.1 | treat_none | 0.000000000 | 0.000000000 | 0.000000000 | 0.000000000 | 0.000000000 | 0.000000000 |
| 0.11 | model | 0.160034763 | 0.129596292 | 0.185931260 | 0.653375011 | 0.586423221 | 0.695047256 |
| 0.11 | treat_all | 0.151612970 | 0.124712893 | 0.180582283 | 0.618991298 | 0.564325843 | 0.667048842 |
| 0.11 | treat_none | 0.000000000 | 0.000000000 | 0.000000000 | 0.000000000 | 0.000000000 | 0.000000000 |
| 0.12 | model | 0.150552486 | 0.122132932 | 0.179013896 | 0.614661654 | 0.552651515 | 0.666247248 |
| 0.12 | treat_all | 0.141972208 | 0.114766449 | 0.171270718 | 0.579630895 | 0.519318182 | 0.632653061 |
| 0.12 | treat_none | 0.000000000 | 0.000000000 | 0.000000000 | 0.000000000 | 0.000000000 | 0.000000000 |
| 0.13 | model | 0.141360259 | 0.114804936 | 0.172583138 | 0.577132486 | 0.505134728 | 0.633193538 |
| 0.13 | treat_all | 0.132109820 | 0.104591351 | 0.161745094 | 0.539365656 | 0.473275862 | 0.597466573 |
| 0.13 | treat_none | 0.000000000 | 0.000000000 | 0.000000000 | 0.000000000 | 0.000000000 | 0.000000000 |
| 0.14 | model | 0.137157908 | 0.107820463 | 0.163026254 | 0.559975520 | 0.466506069 | 0.604572061 |
| 0.14 | treat_all | 0.122018074 | 0.094179622 | 0.151997944 | 0.498164015 | 0.426162791 | 0.561461794 |
| 0.14 | treat_none | 0.000000000 | 0.000000000 | 0.000000000 | 0.000000000 | 0.000000000 | 0.000000000 |
| 0.15 | model | 0.131784205 | 0.104051565 | 0.151067057 | 0.538036267 | 0.463114754 | 0.585850556 |
| 0.15 | treat_all | 0.111688874 | 0.083522912 | 0.142021449 | 0.455992923 | 0.377941176 | 0.524609844 |
| 0.15 | treat_none | 0.000000000 | 0.000000000 | 0.000000000 | 0.000000000 | 0.000000000 | 0.000000000 |
| 0.16 | model | 0.127159519 | 0.100149084 | 0.148469701 | 0.519155030 | 0.434073231 | 0.575726319 |
| 0.16 | treat_all | 0.101113742 | 0.072612470 | 0.131807419 | 0.412817759 | 0.328571429 | 0.486880466 |
| 0.16 | treat_none | 0.000000000 | 0.000000000 | 0.000000000 | 0.000000000 | 0.000000000 | 0.000000000 |
| 0.17 | model | 0.124231734 | 0.095187379 | 0.145277242 | 0.507201739 | 0.415494648 | 0.566743508 |
| 0.17 | treat_all | 0.090283787 | 0.061439127 | 0.121347268 | 0.368602228 | 0.278012048 | 0.448241947 |
| 0.17 | treat_none | 0.000000000 | 0.000000000 | 0.000000000 | 0.000000000 | 0.000000000 | 0.000000000 |
| 0.18 | model | 0.117953555 | 0.093653146 | 0.142276423 | 0.481569778 | 0.402904487 | 0.538480554 |
| 0.18 | treat_all | 0.079189687 | 0.049993262 | 0.110631990 | 0.323308271 | 0.226219512 | 0.408661025 |
| 0.18 | treat_none | 0.000000000 | 0.000000000 | 0.000000000 | 0.000000000 | 0.000000000 | 0.000000000 |
| 0.19 | model | 0.116840598 | 0.090819180 | 0.139019621 | 0.477025898 | 0.389850233 | 0.525054466 |
| 0.19 | treat_all | 0.067821658 | 0.038264784 | 0.099652138 | 0.276895944 | 0.173148148 | 0.368102797 |
| 0.19 | treat_none | 0.000000000 | 0.000000000 | 0.000000000 | 0.000000000 | 0.000000000 | 0.000000000 |
| 0.2 | model | 0.113259669 | 0.086325967 | 0.137661142 | 0.462406015 | 0.375954198 | 0.514097744 |
| 0.2 | treat_all | 0.056169429 | 0.026243094 | 0.088397790 | 0.229323308 | 0.118750000 | 0.326530612 |
| 0.2 | treat_none | 0.000000000 | 0.000000000 | 0.000000000 | 0.000000000 | 0.000000000 | 0.000000000 |
| 0.21 | model | 0.112059118 | 0.077184885 | 0.135802037 | 0.457504521 | 0.338306209 | 0.511247007 |
| 0.21 | treat_all | 0.044222207 | 0.013917057 | 0.076858522 | 0.180546302 | 0.062974684 | 0.283905967 |
| 0.21 | treat_none | 0.000000000 | 0.000000000 | 0.000000000 | 0.000000000 | 0.000000000 | 0.000000000 |
| 0.22 | model | 0.104783492 | 0.070359352 | 0.133517495 | 0.427800270 | 0.304423332 | 0.501126126 |
| 0.22 | treat_all | 0.031968645 | 0.001274968 | 0.065023374 | 0.130518604 | 0.005769231 | 0.240188383 |
| 0.22 | treat_none | 0.000000000 | 0.000000000 | 0.000000000 | 0.000000000 | 0.000000000 | 0.000000000 |
| 0.23 | model | 0.096697041 | 0.067601827 | 0.129463538 | 0.394785665 | 0.292492368 | 0.490391365 |
| 0.23 | treat_all | 0.019396809 | -0.011695487 | 0.052880821 | 0.079191485 | -0.052922078 | 0.195335277 |
| 0.23 | treat_none | 0.000000000 | 0.000000000 | 0.000000000 | 0.000000000 | 0.000000000 | 0.000000000 |
| 0.24 | model | 0.092904914 | 0.067461471 | 0.122031598 | 0.379303522 | 0.291885091 | 0.460297084 |
| 0.24 | treat_all | 0.006494136 | -0.025007270 | 0.040418726 | 0.026513653 | -0.113157895 | 0.149301826 |
| 0.24 | treat_none | 0.000000000 | 0.000000000 | 0.000000000 | 0.000000000 | 0.000000000 | 0.000000000 |
| 0.25 | model | 0.091774095 | 0.063228975 | 0.116635973 | 0.374686717 | 0.277556441 | 0.437862950 |
| 0.25 | treat_all | -0.006752609 | -0.038674033 | 0.027624309 | -0.027568922 | -0.175000000 | 0.102040816 |
| 0.25 | treat_none | 0.000000000 | 0.000000000 | 0.000000000 | 0.000000000 | 0.000000000 | 0.000000000 |
| 0.26 | model | 0.083370663 | 0.058707879 | 0.112164651 | 0.340377972 | 0.256051232 | 0.421052632 |
| 0.26 | treat_all | -0.020357374 | -0.052710169 | 0.014484097 | -0.083113188 | -0.238513514 | 0.053502482 |
| 0.26 | treat_none | 0.000000000 | 0.000000000 | 0.000000000 | 0.000000000 | 0.000000000 | 0.000000000 |
| 0.27 | model | 0.083024294 | 0.055765786 | 0.110963950 | 0.338963848 | 0.245592971 | 0.418426560 |
| 0.27 | treat_all | -0.034334872 | -0.067130856 | 0.000983880 | -0.140179215 | -0.303767123 | 0.003634330 |
| 0.27 | treat_none | 0.000000000 | 0.000000000 | 0.000000000 | 0.000000000 | 0.000000000 | 0.000000000 |
| 0.28 | model | 0.076683037 | 0.044761612 | 0.102568038 | 0.313074353 | 0.195225346 | 0.407282282 |
| 0.28 | treat_all | -0.048700634 | -0.081952118 | -0.012891344 | -0.198830409 | -0.370833333 | -0.047619048 |
| 0.28 | treat_none | 0.000000000 | 0.000000000 | 0.000000000 | 0.000000000 | 0.000000000 | 0.000000000 |
| 0.29 | model | 0.075013618 | 0.042318367 | 0.101756024 | 0.306258604 | 0.183098592 | 0.392129806 |
| 0.29 | treat_all | -0.063471066 | -0.097190880 | -0.027157420 | -0.259133750 | -0.439788732 | -0.100316183 |
| 0.29 | treat_none | 0.000000000 | 0.000000000 | 0.000000000 | 0.000000000 | 0.000000000 | 0.000000000 |
| 0.3 | model | 0.065640621 | 0.039463299 | 0.092870297 | 0.267991407 | 0.172117040 | 0.370798319 |
| 0.3 | treat_all | -0.078663510 | -0.112865036 | -0.041831097 | -0.321160043 | -0.510714286 | -0.154518950 |
| 0.3 | treat_none | 0.000000000 | 0.000000000 | 0.000000000 | 0.000000000 | 0.000000000 | 0.000000000 |
| 0.31 | model | 0.063215630 | 0.035951637 | 0.088397790 | 0.258090879 | 0.156801118 | 0.358450474 |
| 0.31 | treat_all | -0.094296314 | -0.128993514 | -0.056930098 | -0.384984200 | -0.583695652 | -0.210292813 |
| 0.31 | treat_none | 0.000000000 | 0.000000000 | 0.000000000 | 0.000000000 | 0.000000000 | 0.000000000 |
| 0.32 | model | 0.061531795 | 0.031957534 | 0.085364533 | 0.251216276 | 0.142237223 | 0.342011129 |
| 0.32 | treat_all | -0.110388907 | -0.145596360 | -0.072473188 | -0.450685537 | -0.658823529 | -0.267707083 |
| 0.32 | treat_none | 0.000000000 | 0.000000000 | 0.000000000 | 0.000000000 | 0.000000000 | 0.000000000 |
| 0.33 | model | 0.057021522 | 0.026126275 | 0.082062065 | 0.232802155 | 0.113948331 | 0.324879363 |
| 0.33 | treat_all | -0.126961876 | -0.162694813 | -0.088480251 | -0.518348109 | -0.736194030 | -0.326835212 |
| 0.33 | treat_none | 0.000000000 | 0.000000000 | 0.000000000 | 0.000000000 | 0.000000000 | 0.000000000 |
| 0.34 | model | 0.052681511 | 0.023383001 | 0.078436297 | 0.215083162 | 0.101171073 | 0.313168449 |
| 0.34 | treat_all | -0.144037056 | -0.180311401 | -0.104972376 | -0.588061062 | -0.815909091 | -0.387755102 |
| 0.34 | treat_none | 0.000000000 | 0.000000000 | 0.000000000 | 0.000000000 | 0.000000000 | 0.000000000 |
| 0.35 | model | 0.047527978 | 0.017920385 | 0.074656467 | 0.194042799 | 0.077536010 | 0.292076345 |
| 0.35 | treat_all | -0.161637626 | -0.198470038 | -0.121971951 | -0.659919028 | -0.898076923 | -0.450549451 |
| 0.35 | treat_none | 0.000000000 | 0.000000000 | 0.000000000 | 0.000000000 | 0.000000000 | 0.000000000 |
| 0.36 | model | 0.041666667 | 0.015653775 | 0.069463628 | 0.170112782 | 0.068273092 | 0.272804054 |
| 0.36 | treat_all | -0.179788214 | -0.217196133 | -0.139502762 | -0.734022556 | -0.982812500 | -0.515306122 |
| 0.36 | treat_none | 0.000000000 | 0.000000000 | 0.000000000 | 0.000000000 | 0.000000000 | 0.000000000 |
| 0.37 | model | 0.038381712 | 0.013344442 | 0.063521296 | 0.156701277 | 0.057737305 | 0.252734878 |
| 0.37 | treat_all | -0.198515011 | -0.236516706 | -0.157590108 | -0.810478577 | -1.070238095 | -0.582118562 |
| 0.37 | treat_none | 0.000000000 | 0.000000000 | 0.000000000 | 0.000000000 | 0.000000000 | 0.000000000 |
| 0.38 | model | 0.034574942 | 0.012267570 | 0.059020971 | 0.141159350 | 0.052867384 | 0.235649905 |
| 0.38 | treat_all | -0.217845898 | -0.256460524 | -0.176260916 | -0.889400922 | -1.160483871 | -0.651086241 |
| 0.38 | treat_none | 0.000000000 | 0.000000000 | 0.000000000 | 0.000000000 | 0.000000000 | 0.000000000 |
| 0.39 | model | 0.032983124 | 0.010430818 | 0.053316427 | 0.134660422 | 0.046809375 | 0.212873674 |
| 0.39 | treat_all | -0.237810585 | -0.277058237 | -0.195543882 | -0.970910884 | -1.253688525 | -0.722315156 |
| 0.39 | treat_none | 0.000000000 | 0.000000000 | 0.000000000 | 0.000000000 | 0.000000000 | 0.000000000 |
| 0.4 | model | 0.029158993 | 0.009821977 | 0.051872314 | 0.119047619 | 0.043715847 | 0.203828829 |
| 0.4 | treat_all | -0.258440761 | -0.298342541 | -0.215469613 | -1.055137845 | -1.350000000 | -0.795918367 |
| 0.4 | treat_none | 0.000000000 | 0.000000000 | 0.000000000 | 0.000000000 | 0.000000000 | 0.000000000 |
| 0.41 | model | 0.025439336 | 0.009816774 | 0.050785030 | 0.103861348 | 0.043162012 | 0.186326157 |
| 0.41 | treat_all | -0.279770266 | -0.320348347 | -0.236070793 | -1.142219957 | -1.449576271 | -0.872016603 |
| 0.41 | treat_none | 0.000000000 | 0.000000000 | 0.000000000 | 0.000000000 | 0.000000000 | 0.000000000 |
| 0.42 | model | 0.024830126 | 0.008255541 | 0.050295294 | 0.101374125 | 0.036006093 | 0.184529357 |
| 0.42 | treat_all | -0.301835270 | -0.343112974 | -0.257382359 | -1.232304900 | -1.552586207 | -0.950738916 |
| 0.42 | treat_none | 0.000000000 | 0.000000000 | 0.000000000 | 0.000000000 | 0.000000000 | 0.000000000 |
| 0.43 | model | 0.019498562 | 0.004991761 | 0.045022778 | 0.079606912 | 0.021771296 | 0.166903746 |
| 0.43 | treat_all | -0.324674485 | -0.366676359 | -0.279441698 | -1.325550719 | -1.659210526 | -1.032223416 |
| 0.43 | treat_none | 0.000000000 | 0.000000000 | 0.000000000 | 0.000000000 | 0.000000000 | 0.000000000 |
| 0.44 | model | 0.018613523 | 0.006577217 | 0.040055249 | 0.075993555 | 0.028686173 | 0.153598281 |
| 0.44 | treat_all | -0.348329387 | -0.391081294 | -0.302288871 | -1.422126745 | -1.769642857 | -1.116618076 |
| 0.44 | treat_none | 0.000000000 | 0.000000000 | 0.000000000 | 0.000000000 | 0.000000000 | 0.000000000 |
| 0.45 | model | 0.018248786 | 0.002762431 | 0.032228361 | 0.074504443 | 0.012048193 | 0.130553657 |
| 0.45 | treat_all | -0.372844467 | -0.416373682 | -0.325966851 | -1.522214627 | -1.884090909 | -1.204081633 |
| 0.45 | treat_none | 0.000000000 | 0.000000000 | 0.000000000 | 0.000000000 | 0.000000000 | 0.000000000 |
| 0.46 | model | 0.018041061 | 0.003035264 | 0.033728941 | 0.073656363 | 0.012678063 | 0.137705375 |
| 0.46 | treat_all | -0.398267512 | -0.442602824 | -0.350521793 | -1.626009468 | -2.002777778 | -1.294784580 |
| 0.46 | treat_none | 0.000000000 | 0.000000000 | 0.000000000 | 0.000000000 | 0.000000000 | 0.000000000 |
| 0.47 | model | 0.014124883 | 0.000608082 | 0.030716842 | 0.057667754 | 0.002539913 | 0.120371684 |
| 0.47 | treat_all | -0.424649918 | -0.469821745 | -0.376003336 | -1.733721095 | -2.125943396 | -1.388910281 |
| 0.47 | treat_none | 0.000000000 | 0.000000000 | 0.000000000 | 0.000000000 | 0.000000000 | 0.000000000 |
| 0.48 | model | 0.015512112 | -0.002479105 | 0.031945035 | 0.063331405 | -0.010812481 | 0.127545249 |
| 0.48 | treat_all | -0.452047032 | -0.498087548 | -0.402464938 | -1.845575477 | -2.253846154 | -1.486656201 |
| 0.48 | treat_none | 0.000000000 | 0.000000000 | 0.000000000 | 0.000000000 | 0.000000000 | 0.000000000 |
| 0.49 | model | 0.011898314 | -0.004640162 | 0.028472899 | 0.048577326 | -0.019381599 | 0.116246499 |
| 0.49 | treat_all | -0.480518543 | -0.527461813 | -0.429964251 | -1.961816305 | -2.386764706 | -1.588235294 |
| 0.49 | treat_none | 0.000000000 | 0.000000000 | 0.000000000 | 0.000000000 | 0.000000000 | 0.000000000 |
| 0.5 | model | 0.009208103 | -0.004604052 | 0.028545120 | 0.037593985 | -0.019230769 | 0.116541353 |
| 0.5 | treat_all | -0.510128913 | -0.558011050 | -0.458563536 | -2.082706767 | -2.525000000 | -1.693877551 |
| 0.5 | treat_none | 0.000000000 | 0.000000000 | 0.000000000 | 0.000000000 | 0.000000000 | 0.000000000 |
| 0.51 | model | 0.010316834 | -0.005863119 | 0.029804187 | 0.042120608 | -0.024302851 | 0.121681755 |
| 0.51 | treat_all | -0.540947871 | -0.589807194 | -0.488330139 | -2.208531533 | -2.668877551 | -1.803831737 |
| 0.51 | treat_none | 0.000000000 | 0.000000000 | 0.000000000 | 0.000000000 | 0.000000000 | 0.000000000 |
| 0.52 | model | 0.008901166 | -0.006062001 | 0.024554942 | 0.036340852 | -0.026439090 | 0.100250627 |
| 0.52 | treat_all | -0.573050952 | -0.622928177 | -0.519337017 | -2.339598997 | -2.818750000 | -1.918367347 |
| 0.52 | treat_none | 0.000000000 | 0.000000000 | 0.000000000 | 0.000000000 | 0.000000000 | 0.000000000 |
| 0.53 | model | 0.007503624 | -0.009854630 | 0.027643901 | 0.030635098 | -0.043861179 | 0.104240544 |
| 0.53 | treat_all | -0.606520121 | -0.657458564 | -0.551663336 | -2.476243801 | -2.975000000 | -2.037776813 |
| 0.53 | treat_none | 0.000000000 | 0.000000000 | 0.000000000 | 0.000000000 | 0.000000000 | 0.000000000 |
| 0.54 | model | 0.006045320 | -0.010609336 | 0.026303147 | 0.024681268 | -0.047220242 | 0.099184783 |
| 0.54 | treat_all | -0.641444471 | -0.693490271 | -0.585395148 | -2.618829683 | -3.138043478 | -2.162377995 |
| 0.54 | treat_none | 0.000000000 | 0.000000000 | 0.000000000 | 0.000000000 | 0.000000000 | 0.000000000 |
| 0.55 | model | 0.003171680 | -0.009515040 | 0.022815633 | 0.012949039 | -0.042349727 | 0.086883876 |
| 0.55 | treat_all | -0.677921015 | -0.731123389 | -0.620626151 | -2.767752715 | -3.308333333 | -2.292517007 |
| 0.55 | treat_none | 0.000000000 | 0.000000000 | 0.000000000 | 0.000000000 | 0.000000000 | 0.000000000 |
| 0.56 | model | 0.002762431 | -0.011384564 | 0.021513477 | 0.011278195 | -0.049653158 | 0.085895722 |
| 0.56 | treat_all | -0.716055583 | -0.770467102 | -0.657458564 | -2.923444976 | -3.486363636 | -2.428571429 |
| 0.56 | treat_none | 0.000000000 | 0.000000000 | 0.000000000 | 0.000000000 | 0.000000000 | 0.000000000 |
| 0.57 | model | 0.002976573 | -0.009914772 | 0.018373378 | 0.012152474 | -0.041413238 | 0.073358413 |
| 0.57 | treat_all | -0.755963853 | -0.811640755 | -0.696004112 | -3.086378738 | -3.672674419 | -2.570953963 |
| 0.57 | treat_none | 0.000000000 | 0.000000000 | 0.000000000 | 0.000000000 | 0.000000000 | 0.000000000 |
| 0.58 | model | 0.002850127 | -0.013373674 | 0.019643953 | 0.011636233 | -0.057875458 | 0.074074074 |
| 0.58 | treat_all | -0.797772516 | -0.854775059 | -0.736385162 | -3.257071250 | -3.867857143 | -2.720116618 |
| 0.58 | treat_none | 0.000000000 | 0.000000000 | 0.000000000 | 0.000000000 | 0.000000000 | 0.000000000 |
| 0.59 | model | -0.001302610 | -0.013497732 | 0.018775547 | -0.005318173 | -0.058869625 | 0.074964132 |
| 0.59 | treat_all | -0.841620626 | -0.900013475 | -0.778736019 | -3.436090226 | -4.072560976 | -2.876555500 |
| 0.59 | treat_none | 0.000000000 | 0.000000000 | 0.000000000 | 0.000000000 | 0.000000000 | 0.000000000 |
| 0.6 | model | -0.003683241 | -0.014272560 | 0.015653775 | -0.015037594 | -0.056776557 | 0.062500000 |
| 0.6 | treat_all | -0.887661142 | -0.947513812 | -0.823204420 | -3.624060150 | -4.287500000 | -3.040816327 |
| 0.6 | treat_none | 0.000000000 | 0.000000000 | 0.000000000 | 0.000000000 | 0.000000000 | 0.000000000 |
| 0.61 | model | -0.002620768 | -0.010908061 | 0.012962176 | -0.010699826 | -0.045562130 | 0.050213675 |
| 0.61 | treat_all | -0.936062710 | -0.997450064 | -0.869953251 | -3.821669559 | -4.513461538 | -3.213500785 |
| 0.61 | treat_none | 0.000000000 | 0.000000000 | 0.000000000 | 0.000000000 | 0.000000000 | 0.000000000 |
| 0.62 | model | -0.001260056 | -0.011146651 | 0.010758942 | -0.005144440 | -0.048036759 | 0.042956656 |
| 0.62 | treat_all | -0.987011728 | -1.050014539 | -0.919162547 | -4.029679462 | -4.751315789 | -3.395273899 |
| 0.62 | treat_none | 0.000000000 | 0.000000000 | 0.000000000 | 0.000000000 | 0.000000000 | 0.000000000 |
| 0.63 | model | 0.002264696 | -0.012194515 | 0.006744313 | 0.009246088 | -0.052170638 | 0.026927663 |
| 0.63 | treat_all | -1.040714748 | -1.105420337 | -0.971031805 | -4.248933144 | -5.002027027 | -3.586872587 |
| 0.63 | treat_none | 0.000000000 | 0.000000000 | 0.000000000 | 0.000000000 | 0.000000000 | 0.000000000 |
| 0.64 | model | 0.001943933 | -0.012175159 | 0.009105791 | 0.007936508 | -0.049707602 | 0.039397964 |
| 0.64 | treat_all | -1.097401269 | -1.163904236 | -1.025782689 | -4.480367586 | -5.266666667 | -3.789115646 |
| 0.64 | treat_none | 0.000000000 | 0.000000000 | 0.000000000 | 0.000000000 | 0.000000000 | 0.000000000 |
| 0.65 | model | -0.000000000 | -0.015259142 | 0.007892660 | -0.000000000 | -0.057432432 | 0.034149118 |
| 0.65 | treat_all | -1.157327019 | -1.225730071 | -1.083662194 | -4.725026853 | -5.546428571 | -4.002915452 |
| 0.65 | treat_none | 0.000000000 | 0.000000000 | 0.000000000 | 0.000000000 | 0.000000000 | 0.000000000 |
| 0.66 | model | -0.001191637 | -0.015220453 | 0.006554003 | -0.004865104 | -0.055842607 | 0.029411765 |
| 0.66 | treat_all | -1.220777814 | -1.291192720 | -1.144946376 | -4.984077842 | -5.842647059 | -4.229291717 |
| 0.66 | treat_none | 0.000000000 | 0.000000000 | 0.000000000 | 0.000000000 | 0.000000000 | 0.000000000 |
| 0.67 | model | -0.004910988 | -0.017216363 | 0.008259389 | -0.020050125 | -0.065146236 | 0.036781609 |
| 0.67 | treat_all | -1.288074111 | -1.360622803 | -1.209944751 | -5.258828890 | -6.156818182 | -4.469387755 |
| 0.67 | treat_none | 0.000000000 | 0.000000000 | 0.000000000 | 0.000000000 | 0.000000000 | 0.000000000 |
| 0.68 | model | -0.005870166 | -0.015308471 | 0.010013812 | -0.023966165 | -0.062980769 | 0.040129151 |
| 0.68 | treat_all | -1.359576427 | -1.434392265 | -1.279005525 | -5.550751880 | -6.490625000 | -4.724489796 |
| 0.68 | treat_none | 0.000000000 | 0.000000000 | 0.000000000 | 0.000000000 | 0.000000000 | 0.000000000 |
| 0.69 | model | -0.002584210 | -0.018267688 | 0.008376403 | -0.010550570 | -0.067478604 | 0.034327450 |
| 0.69 | treat_all | -1.435691796 | -1.512921048 | -1.352521832 | -5.861508610 | -6.845967742 | -4.996050033 |
| 0.69 | treat_none | 0.000000000 | 0.000000000 | 0.000000000 | 0.000000000 | 0.000000000 | 0.000000000 |
| 0.7 | model | -0.002148557 | -0.016574586 | 0.008287293 | -0.008771930 | -0.065454545 | 0.031980320 |
| 0.7 | treat_all | -1.516881522 | -1.596685083 | -1.430939227 | -6.192982456 | -7.225000000 | -5.285714286 |
| 0.7 | treat_none | 0.000000000 | 0.000000000 | 0.000000000 | 0.000000000 | 0.000000000 | 0.000000000 |
| 0.71 | model | -0.000635042 | -0.019400521 | 0.010636947 | -0.002592689 | -0.071178938 | 0.044659962 |
| 0.71 | treat_all | -1.603670540 | -1.686225948 | -1.514764717 | -6.547316567 | -7.630172414 | -5.595355384 |
| 0.71 | treat_none | 0.000000000 | 0.000000000 | 0.000000000 | 0.000000000 | 0.000000000 | 0.000000000 |
| 0.72 | model | 0.002499342 | -0.018547751 | 0.008681926 | 0.010204082 | -0.068050193 | 0.038961039 |
| 0.72 | treat_all | -1.696658774 | -1.782162589 | -1.604577743 | -6.926960258 | -8.064285714 | -5.927113703 |
| 0.72 | treat_none | 0.000000000 | 0.000000000 | 0.000000000 | 0.000000000 | 0.000000000 | 0.000000000 |
| 0.73 | model | 0.003580929 | -0.011731805 | 0.011322556 | 0.014619883 | -0.047897522 | 0.044551798 |
| 0.73 | treat_all | -1.796535025 | -1.885205648 | -1.701043585 | -7.334725703 | -8.530555556 | -6.283446712 |
| 0.73 | treat_none | 0.000000000 | 0.000000000 | 0.000000000 | 0.000000000 | 0.000000000 | 0.000000000 |
| 0.74 | model | 0.001345800 | -0.011474713 | 0.012112197 | 0.005494505 | -0.046847889 | 0.050397878 |
| 0.74 | treat_all | -1.904094064 | -1.996175096 | -1.804929877 | -7.773857721 | -9.032692308 | -6.667189953 |
| 0.74 | treat_none | 0.000000000 | 0.000000000 | 0.000000000 | 0.000000000 | 0.000000000 | 0.000000000 |
| 0.75 | model | 0.000000000 | -0.008287293 | 0.011049724 | 0.000000000 | -0.036437247 | 0.045977011 |
| 0.75 | treat_all | -2.020257827 | -2.116022099 | -1.917127072 | -8.248120301 | -9.575000000 | -7.081632653 |
| 0.75 | treat_none | 0.000000000 | 0.000000000 | 0.000000000 | 0.000000000 | 0.000000000 | 0.000000000 |
| 0.76 | model | 0.005371393 | -0.008901166 | 0.011049724 | 0.021929825 | -0.039136302 | 0.044252874 |
| 0.76 | treat_all | -2.146101903 | -2.245856354 | -2.038674033 | -8.761904762 | -10.162500000 | -7.530612245 |
| 0.76 | treat_none | 0.000000000 | 0.000000000 | 0.000000000 | 0.000000000 | 0.000000000 | 0.000000000 |
| 0.77 | model | 0.003362959 | -0.005604932 | 0.009808632 | 0.013729977 | -0.022883295 | 0.038455501 |
| 0.77 | treat_all | -2.282888942 | -2.386980543 | -2.170790295 | -9.320366133 | -10.801086957 | -8.018633540 |
| 0.77 | treat_none | 0.000000000 | 0.000000000 | 0.000000000 | 0.000000000 | 0.000000000 | 0.000000000 |
| 0.78 | model | 0.002260171 | -0.005190022 | 0.007366483 | 0.009227614 | -0.021349862 | 0.030651341 |
| 0.78 | treat_all | -2.432111167 | -2.540934204 | -2.314917127 | -9.929596719 | -11.497727273 | -8.551020408 |
| 0.78 | treat_none | 0.000000000 | 0.000000000 | 0.000000000 | 0.000000000 | 0.000000000 | 0.000000000 |
| 0.79 | model | 0.001140051 | -0.002323950 | 0.007366483 | 0.004654493 | -0.008793761 | 0.030651341 |
| 0.79 | treat_all | -2.595545032 | -2.709550118 | -2.472770324 | -10.596849266 | -12.260714286 | -9.134110787 |
| 0.79 | treat_none | 0.000000000 | 0.000000000 | 0.000000000 | 0.000000000 | 0.000000000 | 0.000000000 |
| 0.8 | model | 0.004604052 | -0.002762431 | 0.006445672 | 0.018796992 | -0.011363636 | 0.028688525 |
| 0.8 | treat_all | -2.775322284 | -2.895027624 | -2.646408840 | -11.330827068 | -13.100000000 | -9.775510204 |
| 0.8 | treat_none | 0.000000000 | 0.000000000 | 0.000000000 | 0.000000000 | 0.000000000 | 0.000000000 |
| 0.81 | model | 0.004604052 | -0.003247068 | 0.006445672 | 0.018796992 | -0.014246142 | 0.028688525 |
| 0.81 | treat_all | -2.974023456 | -3.100029078 | -2.838325095 | -12.142065691 | -14.027631579 | -10.484425349 |
| 0.81 | treat_none | 0.000000000 | 0.000000000 | 0.000000000 | 0.000000000 | 0.000000000 | 0.000000000 |
| 0.82 | model | 0.004604052 | -0.001534684 | 0.006445672 | 0.018796992 | -0.006127451 | 0.024137931 |
| 0.82 | treat_all | -3.194802537 | -3.327808471 | -3.051565378 | -13.043441938 | -15.058333333 | -11.272108844 |
| 0.82 | treat_none | 0.000000000 | 0.000000000 | 0.000000000 | 0.000000000 | 0.000000000 | 0.000000000 |
| 0.83 | model | 0.004604052 | -0.003466580 | 0.006445672 | 0.018796992 | -0.014153030 | 0.024137931 |
| 0.83 | treat_all | -3.441555628 | -3.582385440 | -3.289892753 | -14.050862450 | -16.210294118 | -12.152460984 |
| 0.83 | treat_none | 0.000000000 | 0.000000000 | 0.000000000 | 0.000000000 | 0.000000000 | 0.000000000 |
| 0.84 | model | 0.004604052 | -0.005064457 | 0.006445672 | 0.018796992 | -0.020676692 | 0.024137931 |
| 0.84 | treat_all | -3.719152855 | -3.868784530 | -3.558011050 | -15.184210526 | -17.506250000 | -13.142857143 |
| 0.84 | treat_none | 0.000000000 | 0.000000000 | 0.000000000 | 0.000000000 | 0.000000000 | 0.000000000 |
| 0.85 | model | 0.003683241 | 0.000000000 | 0.006445672 | 0.015037594 | 0.000000000 | 0.024137931 |
| 0.85 | treat_all | -4.033763045 | -4.193370166 | -3.861878453 | -16.468671679 | -18.975000000 | -14.265306122 |
| 0.85 | treat_none | 0.000000000 | 0.000000000 | 0.000000000 | 0.000000000 | 0.000000000 | 0.000000000 |
| 0.86 | model | 0.003683241 | 0.000000000 | 0.006445672 | 0.015037594 | 0.000000000 | 0.025270758 |
| 0.86 | treat_all | -4.393317548 | -4.564325178 | -4.209155485 | -17.936627282 | -20.653571429 | -15.548104956 |
| 0.86 | treat_none | 0.000000000 | 0.000000000 | 0.000000000 | 0.000000000 | 0.000000000 | 0.000000000 |
| 0.87 | model | 0.003683241 | 0.000000000 | 0.006445672 | 0.015037594 | 0.000000000 | 0.024137931 |
| 0.87 | treat_all | -4.808188129 | -4.992350191 | -4.609859754 | -19.630422209 | -22.590384615 | -17.028257457 |
| 0.87 | treat_none | 0.000000000 | 0.000000000 | 0.000000000 | 0.000000000 | 0.000000000 | 0.000000000 |
| 0.88 | model | 0.000920810 | 0.000000000 | 0.005524862 | 0.003759398 | 0.000000000 | 0.022988506 |
| 0.88 | treat_all | -5.292203806 | -5.491712707 | -5.077348066 | -21.606516291 | -24.850000000 | -18.755102041 |
| 0.88 | treat_none | 0.000000000 | 0.000000000 | 0.000000000 | 0.000000000 | 0.000000000 | 0.000000000 |
| 0.89 | model | 0.000920810 | 0.000000000 | 0.005524862 | 0.003759398 | 0.000000000 | 0.020689655 |
| 0.89 | treat_all | -5.864222334 | -6.081868408 | -5.629834254 | -23.941900205 | -27.520454545 | -20.795918367 |
| 0.89 | treat_none | 0.000000000 | 0.000000000 | 0.000000000 | 0.000000000 | 0.000000000 | 0.000000000 |
| 0.9 | model | 0.000920810 | 0.000000000 | 0.004604052 | 0.003759398 | 0.000000000 | 0.018796992 |
| 0.9 | treat_all | -6.550644567 | -6.790055249 | -6.292817680 | -26.744360902 | -30.725000000 | -23.244897959 |
| 0.9 | treat_none | 0.000000000 | 0.000000000 | 0.000000000 | 0.000000000 | 0.000000000 | 0.000000000 |
| 0.91 | model | 0.000920810 | 0.000000000 | 0.004604052 | 0.003759398 | 0.000000000 | 0.018050542 |
| 0.91 | treat_all | -7.389605075 | -7.655616943 | -7.103130755 | -30.169590643 | -34.641666667 | -26.238095238 |
| 0.91 | treat_none | 0.000000000 | 0.000000000 | 0.000000000 | 0.000000000 | 0.000000000 | 0.000000000 |
| 0.92 | model | 0.000000000 | 0.000000000 | 0.002762431 | 0.000000000 | 0.000000000 | 0.012295082 |
| 0.92 | treat_all | -8.438305709 | -8.737569061 | -8.116022099 | -34.451127820 | -39.537500000 | -29.979591837 |
| 0.92 | treat_none | 0.000000000 | 0.000000000 | 0.000000000 | 0.000000000 | 0.000000000 | 0.000000000 |
| 0.93 | model | 0.000000000 | 0.000000000 | 0.001841621 | 0.000000000 | 0.000000000 | 0.007352941 |
| 0.93 | treat_all | -9.786635096 | -10.128650355 | -9.418310971 | -39.955961332 | -45.832142857 | -34.790087464 |
| 0.93 | treat_none | 0.000000000 | 0.000000000 | 0.000000000 | 0.000000000 | 0.000000000 | 0.000000000 |
| 0.94 | model | 0.000000000 | 0.000000000 | 0.000920810 | 0.000000000 | 0.000000000 | 0.003610108 |
| 0.94 | treat_all | -11.584407612 | -11.983425414 | -11.154696133 | -47.295739348 | -54.225000000 | -41.204081633 |
| 0.94 | treat_none | 0.000000000 | 0.000000000 | 0.000000000 | 0.000000000 | 0.000000000 | 0.000000000 |
| 0.95 | model | 0.000000000 | 0.000000000 | 0.000000000 | 0.000000000 | 0.000000000 | 0.000000000 |
| 0.95 | treat_all | -14.101289134 | -14.580110497 | -13.585635359 | -57.571428571 | -65.975000000 | -50.183673469 |
| 0.95 | treat_none | 0.000000000 | 0.000000000 | 0.000000000 | 0.000000000 | 0.000000000 | 0.000000000 |
| 0.96 | model | 0.000000000 | 0.000000000 | 0.000000000 | 0.000000000 | 0.000000000 | 0.000000000 |
| 0.96 | treat_all | -17.876611418 | -18.475138122 | -17.232044199 | -72.984962406 | -83.600000000 | -63.653061224 |
| 0.96 | treat_none | 0.000000000 | 0.000000000 | 0.000000000 | 0.000000000 | 0.000000000 | 0.000000000 |
| 0.97 | model | 0.000000000 | 0.000000000 | 0.000000000 | 0.000000000 | 0.000000000 | 0.000000000 |
| 0.97 | treat_all | -24.168815224 | -24.966850829 | -23.309392265 | -98.674185464 | -112.975000000 | -86.102040816 |
| 0.97 | treat_none | 0.000000000 | 0.000000000 | 0.000000000 | 0.000000000 | 0.000000000 | 0.000000000 |
| 0.98 | model | 0.000000000 | 0.000000000 | 0.000000000 | 0.000000000 | 0.000000000 | 0.000000000 |
| 0.98 | treat_all | -36.753222836 | -37.950276243 | -35.464088398 | -150.052631579 | -171.725000000 | -131.000000000 |
| 0.98 | treat_none | 0.000000000 | 0.000000000 | 0.000000000 | 0.000000000 | 0.000000000 | 0.000000000 |
| 0.99 | model | 0.000000000 | 0.000000000 | 0.000000000 | 0.000000000 | 0.000000000 | 0.000000000 |
| 0.99 | treat_all | -74.506445672 | -76.900552486 | -71.928176796 | -304.187969925 | -347.975000000 | -265.693877551 |
| 0.99 | treat_none | 0.000000000 | 0.000000000 | 0.000000000 | 0.000000000 | 0.000000000 | 0.000000000 |
| 1 | model | 0.000000000 | 0.000000000 | 0.000000000 | 0.000000000 | 0.000000000 | 0.000000000 |
| 1 | treat_all | 0.000000000 | 0.000000000 | 0.000000000 | 0.000000000 | 0.000000000 | 0.000000000 |
| 1 | treat_none | 0.000000000 | 0.000000000 | 0.000000000 | 0.000000000 | 0.000000000 | 0.000000000 |
