# Supplementary material for: Pain as a predictor of incident frailty in middle-aged and older adults with metabolic dysfunction-associated steatotic liver disease: a prospective cohort study using machine learning and external validation
Source: Front Endocrinol (Lausanne). 2026 Sep 28;17:1929755. doi: 10.3389/fendo.2026.1929755 (PMC13654746; doi:10.3389/fendo.2026.1929755)
Supplement: Supplementary file 15 [file DataSheet1.zip › Table S17.docx]

| **Table S17.** Net benefit of the logistic regression model across threshold probabilities in the ELSA cohort | | | | | | | |
| --- | --- | --- | --- | --- | --- | --- | --- |
| **threshold** | **curve** | **NB** | **NB_lower** | **NB_upper** | **sNB** | **sNB_lower** | **sNB_upper** |
| 0 | model | 0.211753278 | 0.192326372 | 0.224866440 | 1.000000000 | 1.000000000 | 1.000000000 |
| 0 | treat_all | 0.211753278 | 0.192326372 | 0.224866440 | 1.000000000 | 1.000000000 | 1.000000000 |
| 0 | treat_none | 0.000000000 | 0.000000000 | 0.000000000 | 0.000000000 | 0.000000000 | 0.000000000 |
| 0.01 | model | 0.203791190 | 0.184168053 | 0.217036808 | 0.962399222 | 0.957580859 | 0.965180967 |
| 0.01 | treat_all | 0.203791190 | 0.184168053 | 0.217036808 | 0.962399222 | 0.957580859 | 0.965180967 |
| 0.01 | treat_none | 0.000000000 | 0.000000000 | 0.000000000 | 0.000000000 | 0.000000000 | 0.000000000 |
| 0.02 | model | 0.195666611 | 0.175843237 | 0.209047388 | 0.924031080 | 0.914296021 | 0.929651342 |
| 0.02 | treat_all | 0.195666611 | 0.175843237 | 0.209047388 | 0.924031080 | 0.914296021 | 0.929651342 |
| 0.02 | treat_none | 0.000000000 | 0.000000000 | 0.000000000 | 0.000000000 | 0.000000000 | 0.000000000 |
| 0.03 | model | 0.187374514 | 0.167346775 | 0.200893237 | 0.884871843 | 0.870118713 | 0.893389147 |
| 0.03 | treat_all | 0.187374514 | 0.167346775 | 0.200893237 | 0.884871843 | 0.870118713 | 0.893389147 |
| 0.03 | treat_none | 0.000000000 | 0.000000000 | 0.000000000 | 0.000000000 | 0.000000000 | 0.000000000 |
| 0.04 | model | 0.178909665 | 0.158673304 | 0.192569208 | 0.844896789 | 0.825021044 | 0.856371490 |
| 0.04 | treat_all | 0.178909665 | 0.158673304 | 0.192569208 | 0.844896789 | 0.825021044 | 0.856371490 |
| 0.04 | treat_none | 0.000000000 | 0.000000000 | 0.000000000 | 0.000000000 | 0.000000000 | 0.000000000 |
| 0.05 | model | 0.170394417 | 0.150711894 | 0.184274431 | 0.804683728 | 0.782105263 | 0.819483915 |
| 0.05 | treat_all | 0.170266609 | 0.149817234 | 0.184069937 | 0.804080155 | 0.778973950 | 0.818574514 |
| 0.05 | treat_none | 0.000000000 | 0.000000000 | 0.000000000 | 0.000000000 | 0.000000000 | 0.000000000 |
| 0.06 | model | 0.163000010 | 0.144203445 | 0.177601190 | 0.769763810 | 0.742008175 | 0.788395429 |
| 0.06 | treat_all | 0.161439658 | 0.140772736 | 0.175389830 | 0.762395081 | 0.731947131 | 0.779973347 |
| 0.06 | treat_none | 0.000000000 | 0.000000000 | 0.000000000 | 0.000000000 | 0.000000000 | 0.000000000 |
| 0.07 | model | 0.155906145 | 0.136515795 | 0.170444991 | 0.736263194 | 0.703736559 | 0.757983232 |
| 0.07 | treat_all | 0.152422880 | 0.131533733 | 0.166523054 | 0.719813554 | 0.683908982 | 0.740542047 |
| 0.07 | treat_none | 0.000000000 | 0.000000000 | 0.000000000 | 0.000000000 | 0.000000000 | 0.000000000 |
| 0.08 | model | 0.148594717 | 0.127879722 | 0.162995967 | 0.701735142 | 0.660670934 | 0.726456984 |
| 0.08 | treat_all | 0.143210085 | 0.122093883 | 0.157463522 | 0.676306342 | 0.634826526 | 0.700253545 |
| 0.08 | treat_none | 0.000000000 | 0.000000000 | 0.000000000 | 0.000000000 | 0.000000000 | 0.000000000 |
| 0.09 | model | 0.141304058 | 0.119795697 | 0.157075076 | 0.667305172 | 0.621660066 | 0.695814298 |
| 0.09 | treat_all | 0.133794811 | 0.112446563 | 0.148204879 | 0.631842928 | 0.584665335 | 0.659079581 |
| 0.09 | treat_none | 0.000000000 | 0.000000000 | 0.000000000 | 0.000000000 | 0.000000000 | 0.000000000 |
| 0.1 | model | 0.135232853 | 0.111812638 | 0.151961578 | 0.638634047 | 0.575555556 | 0.674320988 |
| 0.1 | treat_all | 0.124170309 | 0.102584858 | 0.138740489 | 0.586391437 | 0.533389450 | 0.616990641 |
| 0.1 | treat_none | 0.000000000 | 0.000000000 | 0.000000000 | 0.000000000 | 0.000000000 | 0.000000000 |
| 0.11 | model | 0.128064785 | 0.107360942 | 0.141690905 | 0.604783012 | 0.552640449 | 0.636990334 |
| 0.11 | treat_all | 0.114329526 | 0.092501542 | 0.129063416 | 0.539918565 | 0.480961298 | 0.573955881 |
| 0.11 | treat_none | 0.000000000 | 0.000000000 | 0.000000000 | 0.000000000 | 0.000000000 | 0.000000000 |
| 0.12 | model | 0.122433662 | 0.104816990 | 0.137401210 | 0.578190158 | 0.531571876 | 0.612407862 |
| 0.12 | treat_all | 0.104265089 | 0.082189059 | 0.119166409 | 0.492389491 | 0.427341598 | 0.529943059 |
| 0.12 | treat_none | 0.000000000 | 0.000000000 | 0.000000000 | 0.000000000 | 0.000000000 | 0.000000000 |
| 0.13 | model | 0.118325490 | 0.100400261 | 0.129780666 | 0.558789413 | 0.516810345 | 0.596778446 |
| 0.13 | treat_all | 0.093969285 | 0.071639508 | 0.109041885 | 0.443767795 | 0.372489260 | 0.484918448 |
| 0.13 | treat_none | 0.000000000 | 0.000000000 | 0.000000000 | 0.000000000 | 0.000000000 | 0.000000000 |
| 0.14 | model | 0.113376329 | 0.097642793 | 0.126715385 | 0.535417111 | 0.495188452 | 0.578174473 |
| 0.14 | treat_all | 0.083434045 | 0.060844619 | 0.098681907 | 0.394015362 | 0.316361287 | 0.438846753 |
| 0.14 | treat_none | 0.000000000 | 0.000000000 | 0.000000000 | 0.000000000 | 0.000000000 | 0.000000000 |
| 0.15 | model | 0.107390795 | 0.092792046 | 0.122275234 | 0.507150567 | 0.455815203 | 0.559574468 |
| 0.15 | treat_all | 0.072650916 | 0.049795732 | 0.088078165 | 0.343092283 | 0.258912656 | 0.391691018 |
| 0.15 | treat_none | 0.000000000 | 0.000000000 | 0.000000000 | 0.000000000 | 0.000000000 | 0.000000000 |
| 0.16 | model | 0.104465876 | 0.089849441 | 0.118319110 | 0.493337702 | 0.451355662 | 0.529787234 |
| 0.16 | treat_all | 0.061611046 | 0.038483776 | 0.077221952 | 0.290956750 | 0.200096200 | 0.343412527 |
| 0.16 | treat_none | 0.000000000 | 0.000000000 | 0.000000000 | 0.000000000 | 0.000000000 | 0.000000000 |
| 0.17 | model | 0.098854866 | 0.083401113 | 0.116678467 | 0.466839836 | 0.424377714 | 0.520712638 |
| 0.17 | treat_all | 0.050305155 | 0.026899243 | 0.066104145 | 0.237564939 | 0.139862480 | 0.293970699 |
| 0.17 | treat_none | 0.000000000 | 0.000000000 | 0.000000000 | 0.000000000 | 0.000000000 | 0.000000000 |
| 0.18 | model | 0.096767315 | 0.076961348 | 0.109572490 | 0.456981428 | 0.396370638 | 0.493494179 |
| 0.18 | treat_all | 0.038723510 | 0.015032161 | 0.054715171 | 0.182870888 | 0.078159645 | 0.243322973 |
| 0.18 | treat_none | 0.000000000 | 0.000000000 | 0.000000000 | 0.000000000 | 0.000000000 | 0.000000000 |
| 0.19 | model | 0.090652900 | 0.073828240 | 0.106038530 | 0.428106241 | 0.364506173 | 0.470359493 |
| 0.19 | treat_all | 0.026855899 | 0.002872064 | 0.043044988 | 0.126826368 | 0.014933283 | 0.191424686 |
| 0.19 | treat_none | 0.000000000 | 0.000000000 | 0.000000000 | 0.000000000 | 0.000000000 | 0.000000000 |
| 0.2 | model | 0.083535697 | 0.069572608 | 0.102476931 | 0.394495413 | 0.357061503 | 0.448936170 |
| 0.2 | treat_all | 0.014691598 | -0.009592035 | 0.031083050 | 0.069380734 | -0.049873737 | 0.138228942 |
| 0.2 | treat_none | 0.000000000 | 0.000000000 | 0.000000000 | 0.000000000 | 0.000000000 | 0.000000000 |
| 0.21 | model | 0.079699498 | 0.066789212 | 0.093728675 | 0.376379050 | 0.342151899 | 0.427755147 |
| 0.21 | treat_all | 0.002219340 | -0.022371681 | 0.018818279 | 0.010480780 | -0.116321442 | 0.083686470 |
| 0.21 | treat_none | 0.000000000 | 0.000000000 | 0.000000000 | 0.000000000 | 0.000000000 | 0.000000000 |
| 0.22 | model | 0.075441153 | 0.063785009 | 0.089700004 | 0.356269113 | 0.324159021 | 0.410644072 |
| 0.22 | treat_all | -0.010572720 | -0.035479010 | 0.006239026 | -0.049929428 | -0.184472934 | 0.027745473 |
| 0.22 | treat_none | 0.000000000 | 0.000000000 | 0.000000000 | 0.000000000 | 0.000000000 | 0.000000000 |
| 0.23 | model | 0.073380723 | 0.062134563 | 0.087906751 | 0.346538782 | 0.298879610 | 0.396848096 |
| 0.23 | treat_all | -0.023697041 | -0.048926790 | -0.006666961 | -0.111908733 | -0.254394595 | -0.029648537 |
| 0.23 | treat_none | 0.000000000 | 0.000000000 | 0.000000000 | 0.000000000 | 0.000000000 | 0.000000000 |
| 0.24 | model | 0.069374505 | 0.058076225 | 0.086373048 | 0.327619507 | 0.287356322 | 0.387632147 |
| 0.24 | treat_all | -0.037166739 | -0.062728458 | -0.019912579 | -0.175519073 | -0.326156300 | -0.088552916 |
| 0.24 | treat_none | 0.000000000 | 0.000000000 | 0.000000000 | 0.000000000 | 0.000000000 | 0.000000000 |
| 0.25 | model | 0.065727700 | 0.055042901 | 0.082402461 | 0.310397554 | 0.266055046 | 0.370770338 |
| 0.25 | treat_all | -0.050995629 | -0.076898171 | -0.033511413 | -0.240825688 | -0.399831650 | -0.149028078 |
| 0.25 | treat_none | 0.000000000 | 0.000000000 | 0.000000000 | 0.000000000 | 0.000000000 | 0.000000000 |
| 0.26 | model | 0.061207881 | 0.051573186 | 0.078416445 | 0.289052814 | 0.247376789 | 0.352577199 |
| 0.26 | treat_all | -0.065198273 | -0.091450849 | -0.047477784 | -0.307897347 | -0.475498225 | -0.211137704 |
| 0.26 | treat_none | 0.000000000 | 0.000000000 | 0.000000000 | 0.000000000 | 0.000000000 | 0.000000000 |
| 0.27 | model | 0.059358513 | 0.050163998 | 0.073802285 | 0.280319216 | 0.241032908 | 0.334711243 |
| 0.27 | treat_all | -0.079790030 | -0.106402230 | -0.061826794 | -0.376806585 | -0.553237858 | -0.274948963 |
| 0.27 | treat_none | 0.000000000 | 0.000000000 | 0.000000000 | 0.000000000 | 0.000000000 | 0.000000000 |
| 0.28 | model | 0.057875992 | 0.049187847 | 0.070206681 | 0.273318043 | 0.223684211 | 0.325533957 |
| 0.28 | treat_all | -0.094787113 | -0.121768928 | -0.076574389 | -0.447629969 | -0.633136925 | -0.340532757 |
| 0.28 | treat_none | 0.000000000 | 0.000000000 | 0.000000000 | 0.000000000 | 0.000000000 | 0.000000000 |
| 0.29 | model | 0.057740322 | 0.045112833 | 0.067747915 | 0.272677348 | 0.213534078 | 0.323546915 |
| 0.29 | treat_all | -0.110206650 | -0.137568490 | -0.091737408 | -0.520448378 | -0.715286670 | -0.407963983 |
| 0.29 | treat_none | 0.000000000 | 0.000000000 | 0.000000000 | 0.000000000 | 0.000000000 | 0.000000000 |
| 0.3 | model | 0.053007701 | 0.042322903 | 0.066884063 | 0.250327654 | 0.203145478 | 0.305152731 |
| 0.3 | treat_all | -0.126066745 | -0.153819469 | -0.107333657 | -0.595347313 | -0.799783550 | -0.477321814 |
| 0.3 | treat_none | 0.000000000 | 0.000000000 | 0.000000000 | 0.000000000 | 0.000000000 | 0.000000000 |
| 0.31 | model | 0.049714579 | 0.035728615 | 0.062180178 | 0.234775961 | 0.173222912 | 0.291079812 |
| 0.31 | treat_all | -0.142386553 | -0.170541490 | -0.123381971 | -0.672417232 | -0.886729615 | -0.548690018 |
| 0.31 | treat_none | 0.000000000 | 0.000000000 | 0.000000000 | 0.000000000 | 0.000000000 | 0.000000000 |
| 0.32 | model | 0.046967403 | 0.032997172 | 0.058937805 | 0.221802482 | 0.156186613 | 0.275695591 |
| 0.32 | treat_all | -0.159186355 | -0.187755335 | -0.139902294 | -0.751753913 | -0.976232917 | -0.622157286 |
| 0.32 | treat_none | 0.000000000 | 0.000000000 | 0.000000000 | 0.000000000 | 0.000000000 | 0.000000000 |
| 0.33 | model | 0.042253521 | 0.027001950 | 0.058229977 | 0.199541284 | 0.132372620 | 0.267430453 |
| 0.33 | treat_all | -0.176487644 | -0.205483027 | -0.156915761 | -0.833458853 | -1.068407960 | -0.697817607 |
| 0.33 | treat_none | 0.000000000 | 0.000000000 | 0.000000000 | 0.000000000 | 0.000000000 | 0.000000000 |
| 0.34 | model | 0.039884027 | 0.026138019 | 0.054998749 | 0.188351404 | 0.133263671 | 0.252372537 |
| 0.34 | treat_all | -0.194313215 | -0.223747921 | -0.174444788 | -0.917639700 | -1.163376186 | -0.775770666 |
| 0.34 | treat_none | 0.000000000 | 0.000000000 | 0.000000000 | 0.000000000 | 0.000000000 | 0.000000000 |
| 0.35 | model | 0.033660851 | 0.020472970 | 0.050622035 | 0.158962597 | 0.106449106 | 0.235816220 |
| 0.35 | treat_all | -0.212687264 | -0.242574812 | -0.192513169 | -1.004410727 | -1.261266511 | -0.856122279 |
| 0.35 | treat_none | 0.000000000 | 0.000000000 | 0.000000000 | 0.000000000 | 0.000000000 | 0.000000000 |
| 0.36 | model | 0.032752550 | 0.020337542 | 0.050540311 | 0.154673165 | 0.098524306 | 0.227591793 |
| 0.36 | treat_all | -0.231635503 | -0.261990044 | -0.211146187 | -1.093893349 | -1.362215909 | -0.938984881 |
| 0.36 | treat_none | 0.000000000 | 0.000000000 | 0.000000000 | 0.000000000 | 0.000000000 | 0.000000000 |
| 0.37 | model | 0.027559996 | 0.018008434 | 0.043556357 | 0.130151449 | 0.093634760 | 0.201858137 |
| 0.37 | treat_all | -0.251185273 | -0.282021632 | -0.230370730 | -1.186216689 | -1.466370050 | -1.024478042 |
| 0.37 | treat_none | 0.000000000 | 0.000000000 | 0.000000000 | 0.000000000 | 0.000000000 | 0.000000000 |
| 0.38 | model | 0.027088001 | 0.015886196 | 0.040091494 | 0.127922462 | 0.077223851 | 0.181843517 |
| 0.38 | treat_all | -0.271365680 | -0.302699400 | -0.250215419 | -1.281518201 | -1.573884001 | -1.112729046 |
| 0.38 | treat_none | 0.000000000 | 0.000000000 | 0.000000000 | 0.000000000 | 0.000000000 | 0.000000000 |
| 0.39 | model | 0.026098934 | 0.014148202 | 0.039387256 | 0.123251617 | 0.066661665 | 0.176043621 |
| 0.39 | treat_all | -0.292207741 | -0.324055128 | -0.270710754 | -1.379944353 | -1.684923000 | -1.203873526 |
| 0.39 | treat_none | 0.000000000 | 0.000000000 | 0.000000000 | 0.000000000 | 0.000000000 | 0.000000000 |
| 0.4 | model | 0.023636069 | 0.013922616 | 0.035454104 | 0.111620795 | 0.065598780 | 0.163310962 |
| 0.4 | treat_all | -0.313744536 | -0.346122713 | -0.291889267 | -1.481651376 | -1.799663300 | -1.298056156 |
| 0.4 | treat_none | 0.000000000 | 0.000000000 | 0.000000000 | 0.000000000 | 0.000000000 | 0.000000000 |
| 0.41 | model | 0.021797647 | 0.012907368 | 0.034696784 | 0.102938890 | 0.061519146 | 0.159822546 |
| 0.41 | treat_all | -0.336011393 | -0.368938352 | -0.313785695 | -1.586806095 | -1.918293100 | -1.395431416 |
| 0.41 | treat_none | 0.000000000 | 0.000000000 | 0.000000000 | 0.000000000 | 0.000000000 | 0.000000000 |
| 0.42 | model | 0.020582472 | 0.010450336 | 0.033427677 | 0.097200253 | 0.050106526 | 0.153976703 |
| 0.42 | treat_all | -0.359046072 | -0.392540738 | -0.336437172 | -1.695586840 | -2.041013584 | -1.496164445 |
| 0.42 | treat_none | 0.000000000 | 0.000000000 | 0.000000000 | 0.000000000 | 0.000000000 | 0.000000000 |
| 0.43 | model | 0.019060522 | 0.006330786 | 0.032531547 | 0.090012876 | 0.032916888 | 0.151189084 |
| 0.43 | treat_all | -0.382888985 | -0.416971277 | -0.359883439 | -1.808184452 | -2.168040050 | -1.600431965 |
| 0.43 | treat_none | 0.000000000 | 0.000000000 | 0.000000000 | 0.000000000 | 0.000000000 | 0.000000000 |
| 0.44 | model | 0.016963852 | 0.004197599 | 0.029556650 | 0.080111402 | 0.021825397 | 0.137685844 |
| 0.44 | treat_all | -0.407583432 | -0.442274336 | -0.384167071 | -1.924803408 | -2.299603175 | -1.708423326 |
| 0.44 | treat_none | 0.000000000 | 0.000000000 | 0.000000000 | 0.000000000 | 0.000000000 | 0.000000000 |
| 0.45 | model | 0.012892401 | 0.001412866 | 0.029405272 | 0.060884070 | 0.007346189 | 0.134545455 |
| 0.45 | treat_all | -0.433175858 | -0.468497505 | -0.409333745 | -2.045663053 | -2.435950413 | -1.820341645 |
| 0.45 | treat_none | 0.000000000 | 0.000000000 | 0.000000000 | 0.000000000 | 0.000000000 | 0.000000000 |
| 0.46 | model | 0.012447610 | 0.000035976 | 0.028438832 | 0.058783554 | 0.000169895 | 0.127850558 |
| 0.46 | treat_all | -0.459716151 | -0.495691904 | -0.435432518 | -2.170998981 | -2.577347550 | -1.936405088 |
| 0.46 | treat_none | 0.000000000 | 0.000000000 | 0.000000000 | 0.000000000 | 0.000000000 | 0.000000000 |
| 0.47 | model | 0.010070835 | -0.001961018 | 0.026519560 | 0.047559287 | -0.009260862 | 0.121341719 |
| 0.47 | treat_all | -0.487257965 | -0.523912506 | -0.462516151 | -2.301064566 | -2.724080427 | -2.056848282 |
| 0.47 | treat_none | 0.000000000 | 0.000000000 | 0.000000000 | 0.000000000 | 0.000000000 | 0.000000000 |
| 0.48 | model | 0.007509246 | -0.000523032 | 0.022154145 | 0.035462244 | -0.002564103 | 0.101367521 |
| 0.48 | treat_all | -0.515859080 | -0.553218515 | -0.490641462 | -2.436132675 | -2.876456876 | -2.181923908 |
| 0.48 | treat_none | 0.000000000 | 0.000000000 | 0.000000000 | 0.000000000 | 0.000000000 | 0.000000000 |
| 0.49 | model | 0.007332705 | -0.001428449 | 0.022893276 | 0.034628530 | -0.006745818 | 0.106645373 |
| 0.49 | treat_all | -0.545581807 | -0.583673780 | -0.519869725 | -2.576497572 | -3.034808873 | -2.311904459 |
| 0.49 | treat_none | 0.000000000 | 0.000000000 | 0.000000000 | 0.000000000 | 0.000000000 | 0.000000000 |
| 0.5 | model | 0.006799417 | -0.002428363 | 0.019912579 | 0.032110092 | -0.011013216 | 0.091703057 |
| 0.5 | treat_all | -0.576493443 | -0.615347256 | -0.550267120 | -2.722477064 | -3.199494949 | -2.447084233 |
| 0.5 | treat_none | 0.000000000 | 0.000000000 | 0.000000000 | 0.000000000 | 0.000000000 | 0.000000000 |
| 0.51 | model | 0.005471251 | -0.001853485 | 0.016701192 | 0.025837858 | -0.009086492 | 0.076417234 |
| 0.51 | treat_all | -0.608666779 | -0.648313526 | -0.581905224 | -2.874414904 | -3.370902907 | -2.587781549 |
| 0.51 | treat_none | 0.000000000 | 0.000000000 | 0.000000000 | 0.000000000 | 0.000000000 | 0.000000000 |
| 0.52 | model | 0.003763963 | -0.002954509 | 0.014570180 | 0.017775229 | -0.013399413 | 0.065727700 |
| 0.52 | treat_all | -0.642180670 | -0.682653392 | -0.614861583 | -3.032683486 | -3.549452862 | -2.734341253 |
| 0.52 | treat_none | 0.000000000 | 0.000000000 | 0.000000000 | 0.000000000 | 0.000000000 | 0.000000000 |
| 0.53 | model | 0.003503043 | -0.002862369 | 0.014198175 | 0.016543041 | -0.013517470 | 0.066092231 |
| 0.53 | treat_all | -0.677120684 | -0.718454528 | -0.649220340 | -3.197686902 | -3.735600688 | -2.887137540 |
| 0.53 | treat_none | 0.000000000 | 0.000000000 | 0.000000000 | 0.000000000 | 0.000000000 | 0.000000000 |
| 0.54 | model | 0.002386131 | -0.001457018 | 0.012986465 | 0.011268448 | -0.006880734 | 0.060495770 |
| 0.54 | treat_all | -0.713579830 | -0.755812235 | -0.685072956 | -3.369864380 | -3.929841897 | -3.046577143 |
| 0.54 | treat_none | 0.000000000 | 0.000000000 | 0.000000000 | 0.000000000 | 0.000000000 | 0.000000000 |
| 0.55 | model | 0.003021963 | -0.001996654 | 0.011278398 | 0.014271152 | -0.009280161 | 0.052538964 |
| 0.55 | treat_all | -0.751659382 | -0.794830284 | -0.722519022 | -3.549694190 | -4.132716049 | -3.213102952 |
| 0.55 | treat_none | 0.000000000 | 0.000000000 | 0.000000000 | 0.000000000 | 0.000000000 | 0.000000000 |
| 0.56 | model | 0.003355557 | -0.003178948 | 0.011788600 | 0.015846539 | -0.016121809 | 0.052997221 |
| 0.56 | treat_all | -0.791469822 | -0.835621882 | -0.761667182 | -3.737698082 | -4.344811754 | -3.387198115 |
| 0.56 | treat_none | 0.000000000 | 0.000000000 | 0.000000000 | 0.000000000 | 0.000000000 | 0.000000000 |
| 0.57 | model | 0.001784565 | -0.002891447 | 0.009103539 | 0.008427566 | -0.013975325 | 0.044000437 |
| 0.57 | treat_all | -0.833131911 | -0.878310763 | -0.802636186 | -3.934446341 | -4.566772375 | -3.569390728 |
| 0.57 | treat_none | 0.000000000 | 0.000000000 | 0.000000000 | 0.000000000 | 0.000000000 | 0.000000000 |
| 0.58 | model | 0.002012072 | -0.004810472 | 0.010546035 | 0.009501966 | -0.023250615 | 0.050972502 |
| 0.58 | treat_all | -0.876777909 | -0.923032448 | -0.845556095 | -4.140563565 | -4.799302549 | -3.760259179 |
| 0.58 | treat_none | 0.000000000 | 0.000000000 | 0.000000000 | 0.000000000 | 0.000000000 | 0.000000000 |
| 0.59 | model | 0.001338561 | -0.004311826 | 0.010045132 | 0.006321325 | -0.020840490 | 0.046384066 |
| 0.59 | treat_all | -0.922552980 | -0.969935678 | -0.890569658 | -4.356735288 | -5.043175659 | -3.960438287 |
| 0.59 | treat_none | 0.000000000 | 0.000000000 | 0.000000000 | 0.000000000 | 0.000000000 | 0.000000000 |
| 0.6 | model | 0.000485673 | -0.005828072 | 0.010199126 | 0.002293578 | -0.028169014 | 0.046666667 |
| 0.6 | treat_all | -0.970616804 | -1.019184070 | -0.937833900 | -4.583715596 | -5.299242424 | -4.170626350 |
| 0.6 | treat_none | 0.000000000 | 0.000000000 | 0.000000000 | 0.000000000 | 0.000000000 | 0.000000000 |
| 0.61 | model | 0.001494377 | -0.004308788 | 0.008468139 | 0.007057163 | -0.020825810 | 0.040929337 |
| 0.61 | treat_all | -1.021145440 | -1.070958020 | -0.987521949 | -4.822335921 | -5.568440818 | -4.391593288 |
| 0.61 | treat_none | 0.000000000 | 0.000000000 | 0.000000000 | 0.000000000 | 0.000000000 | 0.000000000 |
| 0.62 | model | 0.003195215 | -0.004422177 | 0.010301373 | 0.015089329 | -0.020055646 | 0.048732943 |
| 0.62 | treat_all | -1.074333478 | -1.125456916 | -1.039825158 | -5.073515210 | -5.851807549 | -4.624190065 |
| 0.62 | treat_none | 0.000000000 | 0.000000000 | 0.000000000 | 0.000000000 | 0.000000000 | 0.000000000 |
| 0.63 | model | 0.001667039 | -0.002572752 | 0.010907945 | 0.007872551 | -0.011270845 | 0.053602529 |
| 0.63 | treat_all | -1.130396545 | -1.182901697 | -1.094955568 | -5.338271758 | -6.150491400 | -4.869359640 |
| 0.63 | treat_none | 0.000000000 | 0.000000000 | 0.000000000 | 0.000000000 | 0.000000000 | 0.000000000 |
| 0.64 | model | 0.005234472 | -0.002806109 | 0.009659490 | 0.024719674 | -0.014230980 | 0.047467515 |
| 0.64 | treat_all | -1.189574227 | -1.243537855 | -1.153148778 | -5.617737003 | -6.465768799 | -5.128149748 |
| 0.64 | treat_none | 0.000000000 | 0.000000000 | 0.000000000 | 0.000000000 | 0.000000000 | 0.000000000 |
| 0.65 | model | 0.002983418 | -0.004162908 | 0.010476653 | 0.014089122 | -0.021111893 | 0.048146975 |
| 0.65 | treat_all | -1.252133491 | -1.307638937 | -1.214667314 | -5.913171691 | -6.799062049 | -5.401727862 |
| 0.65 | treat_none | 0.000000000 | 0.000000000 | 0.000000000 | 0.000000000 | 0.000000000 | 0.000000000 |
| 0.66 | model | 0.002828329 | -0.003599691 | 0.009513470 | 0.013356719 | -0.016079325 | 0.044324772 |
| 0.66 | treat_all | -1.318372711 | -1.375510671 | -1.279804588 | -6.225984889 | -7.151960784 | -5.691398806 |
| 0.66 | treat_none | 0.000000000 | 0.000000000 | 0.000000000 | 0.000000000 | 0.000000000 | 0.000000000 |
| 0.67 | model | 0.003738208 | -0.003546882 | 0.010022518 | 0.017653600 | -0.016337875 | 0.046578141 |
| 0.67 | treat_all | -1.388626429 | -1.447495842 | -1.348889576 | -6.557756464 | -7.526247322 | -5.998625565 |
| 0.67 | treat_none | 0.000000000 | 0.000000000 | 0.000000000 | 0.000000000 | 0.000000000 | 0.000000000 |
| 0.68 | model | 0.001942691 | -0.002428363 | 0.009531326 | 0.009174312 | -0.011185682 | 0.042386609 |
| 0.68 | treat_all | -1.463271005 | -1.523980087 | -1.422292375 | -6.910263761 | -7.923926768 | -6.325053996 |
| 0.68 | treat_none | 0.000000000 | 0.000000000 | 0.000000000 | 0.000000000 | 0.000000000 | 0.000000000 |
| 0.69 | model | 0.003227373 | -0.002820035 | 0.008679440 | 0.015241196 | -0.012989825 | 0.040159478 |
| 0.69 | treat_all | -1.542731360 | -1.605398800 | -1.500430839 | -7.285513466 | -8.347262952 | -6.672542326 |
| 0.69 | treat_none | 0.000000000 | 0.000000000 | 0.000000000 | 0.000000000 | 0.000000000 | 0.000000000 |
| 0.7 | model | 0.001942691 | -0.002590254 | 0.009389671 | 0.009174312 | -0.013536379 | 0.041756659 |
| 0.7 | treat_all | -1.627489072 | -1.692245427 | -1.583778533 | -7.685779817 | -8.798821549 | -7.043196544 |
| 0.7 | treat_none | 0.000000000 | 0.000000000 | 0.000000000 | 0.000000000 | 0.000000000 | 0.000000000 |
| 0.71 | model | 0.003500193 | -0.001038335 | 0.010567567 | 0.016529579 | -0.005102461 | 0.047507905 |
| 0.71 | treat_all | -1.718092144 | -1.785081476 | -1.672874345 | -8.113650743 | -9.281522118 | -7.439413123 |
| 0.71 | treat_none | 0.000000000 | 0.000000000 | 0.000000000 | 0.000000000 | 0.000000000 | 0.000000000 |
| 0.72 | model | 0.003260945 | -0.001179491 | 0.008603344 | 0.015399738 | -0.006132756 | 0.038259796 |
| 0.72 | treat_all | -1.815166863 | -1.884548671 | -1.768334143 | -8.572083879 | -9.798701299 | -7.863930886 |
| 0.72 | treat_none | 0.000000000 | 0.000000000 | 0.000000000 | 0.000000000 | 0.000000000 | 0.000000000 |
| 0.73 | model | 0.002032630 | -0.001906715 | 0.010486932 | 0.009599049 | -0.009369752 | 0.046636269 |
| 0.73 | treat_all | -1.919432303 | -1.991383807 | -1.870865037 | -9.064475025 | -10.354190049 | -8.319894408 |
| 0.73 | treat_none | 0.000000000 | 0.000000000 | 0.000000000 | 0.000000000 | 0.000000000 | 0.000000000 |
| 0.74 | model | 0.003063474 | -0.002204207 | 0.009713453 | 0.014467184 | -0.010805861 | 0.044943820 |
| 0.74 | treat_all | -2.031718160 | -2.106437031 | -1.981282923 | -9.594742414 | -10.952408702 | -8.810932049 |
| 0.74 | treat_none | 0.000000000 | 0.000000000 | 0.000000000 | 0.000000000 | 0.000000000 | 0.000000000 |
| 0.75 | model | 0.001942691 | -0.000971345 | 0.008742108 | 0.009174312 | -0.004761905 | 0.040449438 |
| 0.75 | treat_all | -2.152986887 | -2.230694512 | -2.100534240 | -10.167431193 | -11.598484848 | -9.341252700 |
| 0.75 | treat_none | 0.000000000 | 0.000000000 | 0.000000000 | 0.000000000 | 0.000000000 | 0.000000000 |
| 0.76 | model | 0.003399709 | -0.000161891 | 0.006313745 | 0.016055046 | -0.000749064 | 0.028824834 |
| 0.76 | treat_all | -2.284361340 | -2.365306783 | -2.229723167 | -10.787844037 | -12.298400673 | -9.915766739 |
| 0.76 | treat_none | 0.000000000 | 0.000000000 | 0.000000000 | 0.000000000 | 0.000000000 | 0.000000000 |
| 0.77 | model | 0.002428363 | -0.000506789 | 0.005342399 | 0.011467890 | -0.002449479 | 0.024390244 |
| 0.77 | treat_all | -2.427159660 | -2.511624469 | -2.370145913 | -11.462205824 | -13.059178744 | -10.540238520 |
| 0.77 | treat_none | 0.000000000 | 0.000000000 | 0.000000000 | 0.000000000 | 0.000000000 | 0.000000000 |
| 0.78 | model | 0.002428363 | 0.000485673 | 0.005298247 | 0.011467890 | 0.002293578 | 0.024390244 |
| 0.78 | treat_all | -2.582939644 | -2.671243764 | -2.523334364 | -12.197873228 | -13.889118457 | -11.221480463 |
| 0.78 | treat_none | 0.000000000 | 0.000000000 | 0.000000000 | 0.000000000 | 0.000000000 | 0.000000000 |
| 0.79 | model | 0.001942691 | 0.000000000 | 0.005087999 | 0.009174312 | 0.000000000 | 0.024591996 |
| 0.79 | treat_all | -2.753555818 | -2.846064895 | -2.691112190 | -13.003604194 | -14.798100048 | -11.967602592 |
| 0.79 | treat_none | 0.000000000 | 0.000000000 | 0.000000000 | 0.000000000 | 0.000000000 | 0.000000000 |
| 0.8 | model | 0.001942691 | 0.000000000 | 0.004371054 | 0.009174312 | 0.000000000 | 0.019955654 |
| 0.8 | treat_all | -2.941233609 | -3.038368140 | -2.875667800 | -13.889908257 | -15.797979798 | -12.788336933 |
| 0.8 | treat_none | 0.000000000 | 0.000000000 | 0.000000000 | 0.000000000 | 0.000000000 | 0.000000000 |
| 0.81 | model | 0.001457018 | 0.000000000 | 0.002914036 | 0.006880734 | 0.000000000 | 0.013793103 |
| 0.81 | treat_all | -3.148666956 | -3.250913831 | -3.079650316 | -14.869507484 | -16.903110048 | -13.695464363 |
| 0.81 | treat_none | 0.000000000 | 0.000000000 | 0.000000000 | 0.000000000 | 0.000000000 | 0.000000000 |
| 0.82 | model | 0.001457018 | 0.000000000 | 0.002914036 | 0.006880734 | 0.000000000 | 0.013100437 |
| 0.82 | treat_all | -3.379148454 | -3.487075711 | -3.306297555 | -15.957951070 | -18.131032548 | -14.703383729 |
| 0.82 | treat_none | 0.000000000 | 0.000000000 | 0.000000000 | 0.000000000 | 0.000000000 | 0.000000000 |
| 0.83 | model | 0.000485673 | 0.000000000 | 0.002914036 | 0.002293578 | 0.000000000 | 0.014084507 |
| 0.83 | treat_all | -3.636745422 | -3.751021341 | -3.559609176 | -17.174446843 | -19.503416518 | -15.829881845 |
| 0.83 | treat_none | 0.000000000 | 0.000000000 | 0.000000000 | 0.000000000 | 0.000000000 | 0.000000000 |
| 0.84 | model | 0.000485673 | 0.000000000 | 0.002914036 | 0.002293578 | 0.000000000 | 0.014084507 |
| 0.84 | treat_all | -3.926542011 | -4.047960175 | -3.844584750 | -18.543004587 | -21.047348485 | -17.097192225 |
| 0.84 | treat_none | 0.000000000 | 0.000000000 | 0.000000000 | 0.000000000 | 0.000000000 | 0.000000000 |
| 0.85 | model | 0.000485673 | 0.000000000 | 0.001457018 | 0.002293578 | 0.000000000 | 0.006880734 |
| 0.85 | treat_all | -4.254978145 | -4.384490853 | -4.167557067 | -20.094036697 | -22.797138047 | -18.533477322 |
| 0.85 | treat_none | 0.000000000 | 0.000000000 | 0.000000000 | 0.000000000 | 0.000000000 | 0.000000000 |
| 0.86 | model | 0.000000000 | 0.000000000 | 0.000971345 | 0.000000000 | 0.000000000 | 0.004694836 |
| 0.86 | treat_all | -4.630333726 | -4.769097343 | -4.536668286 | -21.866644823 | -24.796897547 | -20.174946004 |
| 0.86 | treat_none | 0.000000000 | 0.000000000 | 0.000000000 | 0.000000000 | 0.000000000 | 0.000000000 |
| 0.87 | model | 0.000000000 | 0.000000000 | 0.000971345 | 0.000000000 | 0.000000000 | 0.004694836 |
| 0.87 | treat_all | -5.063436321 | -5.212874061 | -4.962565846 | -23.911961891 | -27.104312354 | -22.068948330 |
| 0.87 | treat_none | 0.000000000 | 0.000000000 | 0.000000000 | 0.000000000 | 0.000000000 | 0.000000000 |
| 0.88 | model | 0.000000000 | 0.000000000 | 0.000485673 | 0.000000000 | 0.000000000 | 0.002347418 |
| 0.88 | treat_all | -5.568722681 | -5.730613566 | -5.459446333 | -26.298165138 | -29.796296296 | -24.278617711 |
| 0.88 | treat_none | 0.000000000 | 0.000000000 | 0.000000000 | 0.000000000 | 0.000000000 | 0.000000000 |
| 0.89 | model | 0.000000000 | 0.000000000 | 0.000485673 | 0.000000000 | 0.000000000 | 0.002159827 |
| 0.89 | treat_all | -6.165879288 | -6.342487527 | -6.046668727 | -29.118223520 | -32.977731864 | -26.890045160 |
| 0.89 | treat_none | 0.000000000 | 0.000000000 | 0.000000000 | 0.000000000 | 0.000000000 | 0.000000000 |
| 0.9 | model | 0.000000000 | 0.000000000 | 0.000000000 | 0.000000000 | 0.000000000 | 0.000000000 |
| 0.9 | treat_all | -6.882467217 | -7.076736280 | -6.751335600 | -32.502293578 | -36.795454545 | -30.023758099 |
| 0.9 | treat_none | 0.000000000 | 0.000000000 | 0.000000000 | 0.000000000 | 0.000000000 | 0.000000000 |
| 0.91 | model | 0.000000000 | 0.000000000 | 0.000000000 | 0.000000000 | 0.000000000 | 0.000000000 |
| 0.91 | treat_all | -7.758296908 | -7.974151422 | -7.612595111 | -36.638379205 | -41.461560045 | -33.853851692 |
| 0.91 | treat_none | 0.000000000 | 0.000000000 | 0.000000000 | 0.000000000 | 0.000000000 | 0.000000000 |
| 0.92 | model | 0.000000000 | 0.000000000 | 0.000000000 | 0.000000000 | 0.000000000 | 0.000000000 |
| 0.92 | treat_all | -8.853084021 | -9.095920350 | -8.689169500 | -41.808486239 | -47.294191919 | -38.641468683 |
| 0.92 | treat_none | 0.000000000 | 0.000000000 | 0.000000000 | 0.000000000 | 0.000000000 | 0.000000000 |
| 0.93 | model | 0.000000000 | 0.000000000 | 0.000000000 | 0.000000000 | 0.000000000 | 0.000000000 |
| 0.93 | treat_all | -10.260667453 | -10.538194685 | -10.073336571 | -48.455766710 | -54.793290043 | -44.796976242 |
| 0.93 | treat_none | 0.000000000 | 0.000000000 | 0.000000000 | 0.000000000 | 0.000000000 | 0.000000000 |
| 0.94 | model | 0.000000000 | 0.000000000 | 0.000000000 | 0.000000000 | 0.000000000 | 0.000000000 |
| 0.94 | treat_all | -12.137445362 | -12.461227133 | -11.918892666 | -57.318807339 | -64.792087542 | -53.004319654 |
| 0.94 | treat_none | 0.000000000 | 0.000000000 | 0.000000000 | 0.000000000 | 0.000000000 | 0.000000000 |
| 0.95 | model | 0.000000000 | 0.000000000 | 0.000000000 | 0.000000000 | 0.000000000 | 0.000000000 |
| 0.95 | treat_all | -14.764934434 | -15.153472559 | -14.502671200 | -69.727064220 | -78.790404040 | -64.494600432 |
| 0.95 | treat_none | 0.000000000 | 0.000000000 | 0.000000000 | 0.000000000 | 0.000000000 | 0.000000000 |
| 0.96 | model | 0.000000000 | 0.000000000 | 0.000000000 | 0.000000000 | 0.000000000 | 0.000000000 |
| 0.96 | treat_all | -18.706168043 | -19.191840699 | -18.378339000 | -88.339449541 | -99.787878788 | -81.730021598 |
| 0.96 | treat_none | 0.000000000 | 0.000000000 | 0.000000000 | 0.000000000 | 0.000000000 | 0.000000000 |
| 0.97 | model | 0.000000000 | 0.000000000 | 0.000000000 | 0.000000000 | 0.000000000 | 0.000000000 |
| 0.97 | treat_all | -25.274890724 | -25.922454266 | -24.837785333 | -119.360091743 | -134.783670034 | -110.455723542 |
| 0.97 | treat_none | 0.000000000 | 0.000000000 | 0.000000000 | 0.000000000 | 0.000000000 | 0.000000000 |
| 0.98 | model | 0.000000000 | 0.000000000 | 0.000000000 | 0.000000000 | 0.000000000 | 0.000000000 |
| 0.98 | treat_all | -38.412336085 | -39.383681399 | -37.756677999 | -181.401376147 | -204.775252525 | -167.907127430 |
| 0.98 | treat_none | 0.000000000 | 0.000000000 | 0.000000000 | 0.000000000 | 0.000000000 | 0.000000000 |
| 0.99 | model | 0.000000000 | 0.000000000 | 0.000000000 | 0.000000000 | 0.000000000 | 0.000000000 |
| 0.99 | treat_all | -77.824672171 | -79.767362797 | -76.513355998 | -367.525229358 | -414.750000000 | -340.261339093 |
| 0.99 | treat_none | 0.000000000 | 0.000000000 | 0.000000000 | 0.000000000 | 0.000000000 | 0.000000000 |
| 1 | model | 0.000000000 | 0.000000000 | 0.000000000 | 0.000000000 | 0.000000000 | 0.000000000 |
| 1 | treat_all | 0.000000000 | 0.000000000 | 0.000000000 | 0.000000000 | 0.000000000 | 0.000000000 |
| 1 | treat_none | 0.000000000 | 0.000000000 | 0.000000000 | 0.000000000 | 0.000000000 | 0.000000000 |
