# Supplementary material for: Pain as a predictor of incident frailty in middle-aged and older adults with metabolic dysfunction-associated steatotic liver disease: a prospective cohort study using machine learning and external validation
Source: Front Endocrinol (Lausanne). 2026 Sep 28;17:1929755. doi: 10.3389/fendo.2026.1929755 (PMC13654746; doi:10.3389/fendo.2026.1929755)
Supplement: Supplementary file 15 [file DataSheet1.zip › Table S18.docx]

| **Table S18.** Modified Poisson regression and population attributable fraction for the association between pain and risk of frailty (complete datasets) | | | | |
| --- | --- | --- | --- | --- |
| **Cohort** | **Model** | **RR (95% CI)** | **P value** | **PAF (95% CI)** |
| CHARLS | Model 1 | 2.055 (1.791–2.357) | <0.001 | — |
|  | Model 2 | 2.000 (1.743–2.293) | <0.001 | — |
|  | Model 3 | 1.995 (1.740–2.289) | <0.001 | — |
|  | Model 4 | 1.190 (1.030–1.375) | 0.018 | 7.16% (1.20%–13.38%) |
| ELSA | Model 1 | 2.155 (1.765–2.632) | <0.001 | — |
|  | Model 2 | 2.210 (1.823–2.680) | <0.001 | — |
|  | Model 3 | 2.177 (1.796–2.639) | <0.001 | — |
|  | Model 4 | 1.377 (1.125–1.686) | 0.002 | 14.55% (4.75%–24.15%) |
| Model 1: unadjusted. Model 2: adjusted for age, gender, marital status and education. Model 3: additionally adjusted for smoking and drinking. Model 4 (main model): additionally adjusted for baseline frailty index. Complete datasets: participants with complete data on all Model 4 covariates AND all 33 frailty-index items at both waves (no imputation anywhere; CHARLS n = 2,841; ELSA n = 1,579). PAF was estimated by g-computation under Model 4 with 1,000 bootstrap resamples.  CHARLS, China Health and Retirement Longitudinal Study; CI, confidence interval; ELSA, English Longitudinal Study of Ageing; PAF, population attributable fraction; RR, risk ratio. | | | | |
