# Supplementary material for: Pain as a predictor of incident frailty in middle-aged and older adults with metabolic dysfunction-associated steatotic liver disease: a prospective cohort study using machine learning and external validation
Source: Front Endocrinol (Lausanne). 2026 Sep 28;17:1929755. doi: 10.3389/fendo.2026.1929755 (PMC13654746; doi:10.3389/fendo.2026.1929755)
Supplement: Supplementary file 15 [file DataSheet1.zip › Table S19.docx]

| **Table S19.** Sensitivity analyses for the association between pain and incident frailty. | | | | |
| --- | --- | --- | --- | --- |
| **Cohort** | **Sensitivity analysis** | **N** | **RR (95% CI)** | **P value** |
| CHARLS | TyG-WC (M>=800/F>=600) | 3829 | 1.204 (1.078–1.345) | <0.001 |
| CHARLS | Exclude baseline drinkers | 2681 | 1.218 (1.074–1.381) | 0.002 |
| CHARLS | FI-29 outcome | 3622 | 1.224 (1.097–1.366) | <0.001 |
| CHARLS | Complete case | 2841 | 1.190  (1.030–1.375) | 0.018 |
| ELSA | FI-29 outcome | 2059 | 1.236 (1.053–1.451) | 0.010 |
| ELSA | Complete case | 1579 | 1.377 (1.125–1.686) | 0.002 |
| Sensitivity analyses used Model 4. MASLD redefined by the TyG‑WC with sex-specific cut-offs (men ≥800, women ≥600) in CHARLS. Baseline drinkers (CHARLS: any alcohol in the past year) were excluded; ELSA was not evaluable for this exclusion because only 149 non-drinkers remained. FI-29: frailty index restricted to the 29 harmonized items. Complete case: participants with complete data on all Model 4 covariates.  CI, confidence interval; FI, frailty index; MASLD, metabolic dysfunction-associated steatotic liver disease; RR, risk ratio; TyG-WC, triglyceride-glucose-waist circumference product. | | | | |
