# Supplementary material for: Pain as a predictor of incident frailty in middle-aged and older adults with metabolic dysfunction-associated steatotic liver disease: a prospective cohort study using machine learning and external validation
Source: Front Endocrinol (Lausanne). 2026 Sep 28;17:1929755. doi: 10.3389/fendo.2026.1929755 (PMC13654746; doi:10.3389/fendo.2026.1929755)
Supplement: Supplementary file 15 [file DataSheet1.zip › Table S15.docx]

| **Table S15.** Logistic regression coefficients for the prediction model in the ELSA cohort | | | | |
| --- | --- | --- | --- | --- |
| **Variable** | **Coefficient** | **OR** | **Lower_CI** | **Upper_CI** |
| Intercept | -8.388386667 | 0.0002274940061 | 6.738264281e-06 | 0.007680542149 |
| Agey | 0.06864674366 | 1.071057784 | 1.050723424 | 1.09178567 |
| Grip | -0.04212695775 | 0.9587480523 | 0.9433096707 | 0.9744391014 |
| Hearing_Problem=1 | 0.5340170836 | 1.705770788 | 1.273435899 | 2.284884526 |
| Hearte=1 | 0.411999196 | 1.509833222 | 1.07605055 | 2.118484451 |
| Itearn | -8.233923316e-06 | 0.9999917661 | 0.9999773509 | 1.000006182 |
| Mbmi | 0.04989935618 | 1.051165298 | 1.000757563 | 1.104112049 |
| Mheight | -0.04770401708 | 0.9534159401 | 0.1232804104 | 7.373450104 |
| Mstat=1 | -0.3892146551 | 0.6775888065 | 0.5178319956 | 0.8866323337 |
| Mwaist | 0.01189904284 | 1.011970118 | 0.9914625253 | 1.032901894 |
| Pain_Problem=1 | 0.836765154 | 2.308885993 | 1.811872009 | 2.942235711 |
| Rxhibp_c=1 | 0.3046217048 | 1.356111895 | 1.056349517 | 1.740938433 |
| Sleeprl=1 | 0.3702523943 | 1.448100061 | 1.125071492 | 1.86387603 |
| Stroke=1 | 0.6226494517 | 1.863859712 | 0.8023099243 | 4.329963922 |
| Systo | 0.00693796427 | 1.006962088 | 0.9997658318 | 1.014210142 |
| Agey, age (years); Grip, grip strength (kg); Hearing_Problem, self-reported hearing problem; Hearte, heart disease; Itearn, individual earnings after tax; Mbmi, body mass index (kg/m²); Mheight, height (metres); Mstat, marital status; Mwaist, waist circumference (cm); Pain_Problem, self-reported bodily pain; Rxhibp_c, antihypertensive medication use; Sleeprl, restless sleep; Stroke, stroke; Systo, systolic blood pressure (mmHg).  CI, confidence interval; Coefficient, regression coefficient from the logistic regression model (log-odds scale); OR, odds ratio. | | | | |
